# Correcting palindromes in long reads after whole-genome amplification

**DOI:** 10.1186/s12864-018-5164-1

**Published:** 2018-11-06

**Authors:** Sven Warris, Elio Schijlen, Henri van de Geest, Rahulsimham Vegesna, Thamara Hesselink, Bas te Lintel Hekkert, Gabino Sanchez Perez, Paul Medvedev, Kateryna D. Makova, Dick de Ridder

**Affiliations:** 1 Applied Bioinformatics, Wageningen University and Research, Wageningen, The Netherlands; 2 Bioinformatics Group, Wageningen University and Research, Wageningen, The Netherlands; 3 Bioinformatics and Genomics Graduate Program, Pennsylvania State University, University Park, State College, PA 16802 USA; 4 Computation, Bioinformatics, Statistics Graduate Training Program, Pennsylvania State University, University Park, State College, PA 16802 USA; 5 The Center for Computational Biology and Bioinformatics, Pennsylvania State University, University Park, State College, PA 16802 USA; 6 Department of Computer Science and Engineering, Pennsylvania State University, University Park, State College, PA 16802 USA; 7 Department of Biochemistry and Molecular Biology, Pennsylvania State University, University Park, State College, PA 16802 USA; 8 The Center for Medical Genomics, Pennsylvania State University, University Park, State College, PA 16802 USA; 9 Department of Biology, Pennsylvania State University, University Park, State College, PA 16802 USA; 10 Present address Genetwister Technologies BV, Wageningen, The Netherlands

**Keywords:** Whole-genome amplification, High molecular weight DNA, Long read sequencing, Palindromes, Chimeric reads, de novo assembly, Read mapping

## Abstract

**Background:**

Next-generation sequencing requires sufficient DNA to be available. If limited, whole-genome amplification is applied to generate additional amounts of DNA. Such amplification often results in many chimeric DNA fragments, in particular artificial palindromic sequences, which limit the usefulness of long sequencing reads.

**Results:**

Here, we present Pacasus, a tool for correcting such errors. Two datasets show that it markedly improves read mapping and de novo assembly, yielding results similar to these that would be obtained with non-amplified DNA.

**Conclusions:**

With Pacasus long-read technologies become available for sequencing targets with very small amounts of DNA, such as single cells or even single chromosomes.

**Electronic supplementary material:**

The online version of this article (10.1186/s12864-018-5164-1) contains supplementary material, which is available to authorized users.

## Background

Modern sequencers require sufficient material to work with: the Illumina and Pacific Bioscience (PacBio) platforms prescribe at least three micrograms, but recommend at least five [1] micrograms. Long-read sequencing technologies such as those offered by PacBio and Oxford Nanopore Technology (ONT) additionally require high molecular weight (HMW) DNA as a starting material, i.e. material in which individual DNA stretches are long. In many biological settings, obtaining sufficient amounts of DNA of the required quality and length is problematic, such as in studies on single cells [2, 3] or single selected chromosomes [4]. To overcome this limitation DNA is amplified, starting from as little as picograms, in a process called whole-genome amplification (WGA) [5].

**Fig. 1:**
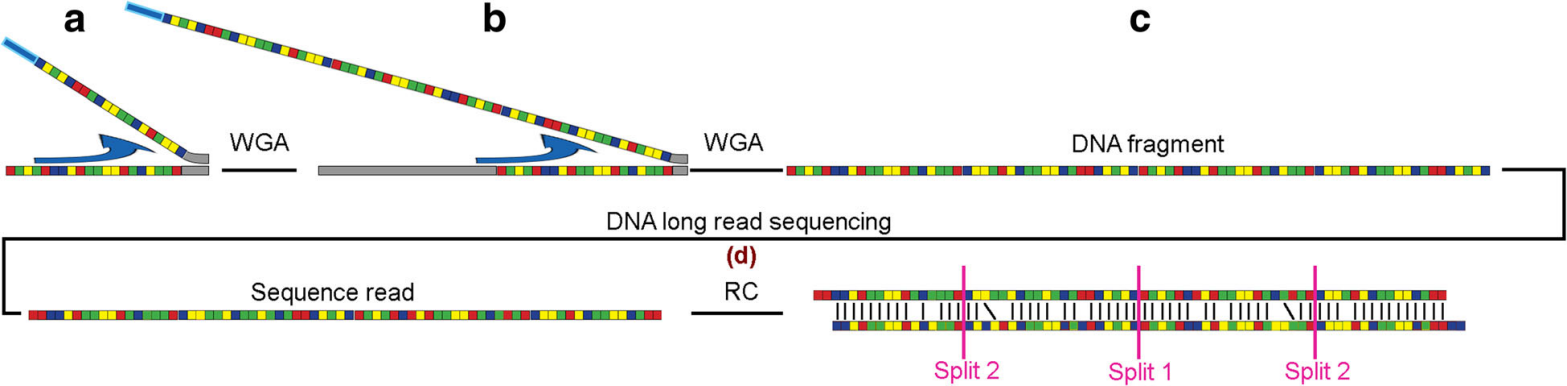
The introduction of palindromes by whole-genome amplification (WGA) and correction of these sequences with Pacasus. The colored squares in this figure indicate the four different nucleotides. In whole-genome amplification a DNA-polymerase binds to the DNA and starts making a copy of that strand (left-side of the image). Palindromes are introduced when during WGA the DNA-polymerase continues with elongation (indicated by the arrow) along an already created WGA product (**a**), generating a palindrome. In this example this incorrect elongation occurs several times (**b**), resulting in a DNA fragment containing four copies of the original fragment (**c**), which is sequenced. Pacasus detects the palindrome sequence by aligning the read’s reverse complement to itself (**d**) and splits the read in two smaller reads at the center of the alignment (split 1). This process is repeated and splits the two resulting reads again (split 2), yielding four separate, ‘clean’, reads. The full set of reads, corrected and left intact, is then used in, for example, read mapping or de novo assembly

A major issue with the WGA process is that it introduces specific chimeric fragments [6, 7] consisting of one or more inverted repeats (Fig. 1), so-called palindromes. This effect is partially alleviated by de-branching, however, chimeric fragments still remain [8]. In Illumina paired-end (PE) sequencing these fragments are then sheared into small sub-fragments before library preparation, which reduces the effect on subsequent analyses of the palindromic fragments as they will occur in only few reads. In other approaches to sequencing, however, the full fragments are used. For Illumina mate-pair (MP) sequencing, long palindromic fragments will result in pairs with incorrect directions and unpredictable insert sizes. As a result, short read MP libraries based on WGA are problematic for read mapping and de novo assembly.

Tools specifically aiming to detect and correct chimeric reads have been proposed (e.g. uchime [9]) and work well for paired-end and single-end short-read technologies.

For long reads however, the palindromic nature of the sequence hinders read mapping and renders de novo assembly highly problematic. Due to the high base-calling error rate of the long read technologies (11–38%) [10, 11], finding and correcting these palindromic constructs in long reads cannot be done by exact string matching. Algorithms for improving long-read quality in general are available: Proovread [12], PBcR [13] and ECTools [14] use either Illumina HiSeq reads or assembled contigs based on HiSeq data to increase the quality of base calling. While Proovread can also detect chimeric fragments, it specifically aims at detecting PCR artifacts joining fragments originating from different regions in the genome. This is done by mapping HiSeq reads, assumed to be available, which do not have these chimeras. The location of the chimera in the long read is then detected by finding discrepancies in the short-read mappings. This approach is unfit for solving the chimeras occurring due to WGA: the HiSeq reads are based on the same fragments as the long reads and will therefore contain the same nucleotide sequence information. As a consequence of this lack of suitable methods to correct these chimeras, the use of WGA with long-read technologies was usually not advised [6], which precludes the application of long read technology to answer essential biological questions at the single-chromosome or single-cell level.

Here, we introduce a new method, *Pacasus*, for correcting palindromic, long, error-rich reads without the loss of nucleotide information and with only very limited impact on repeats and palindrome sequences of biological origin. The method relies on a Smith-Waterman alignment implementation called pyPaSWAS [15, 16], which supports fast processing on multicore CPUs, GPUs and Xeon Phis to detect palindromes and iteratively corrects them by splitting up reads. We demonstrate its performance on PacBio sequencing data of *Arabidopsis thaliana* as well as on flow-sorted gorilla Y chromosome data, by using the multiple displacement amplification kit REPLI-g for the amplification process.

The gorilla Y chromosome was selected because primate Y chromosomes are relatively short and contain many repeats, rendering them difficult to sequence and assemble. Even in one of the most complete assemblies, that of *Homo sapiens*, more than half of the sequence of Y is still unknown [17]. To obtain a higher read coverage of the gorilla Y chromosome, a recent paper [4] used flow-sorting and WGA. PacBio long reads, Illumina HiSeq PE and MP-libraries, transcriptome data and PCR sequences were used by the authors as well (Bioproject PRJNA293447). The RecoverY tool [4, 18] presented in same paper was designed to identify short reads originating from the Y chromosome. Based on these data, the authors created a hybrid (PacBio + HiSeq) assembly, here labeled ‘GorY’. The authors also used HGAP [19] and MHAP [20] to create PacBio-only assemblies, but these resulting assemblies were of suboptimal quality. In this manuscript, we used the PacBio data after WGA generated by Tomaszkiewicz and colleagues [4] to show the benefits of correcting palindrome sequences in this data set with an improved quality of the PacBio-only de novo genome assembly.

## Results

### Pacasus corrects many palindromic sequences found in WGA data

To demonstrate the added value of Pacasus in the analysis of PacBio reads generated from WGA DNA samples, we applied it to several data sets of *Arabidopsis thaliana* and a data set of the gorilla Y chromosome [4] (Table 7). Table 1 lists the original number of reads, the number of reads that were found to have chimeras and the number of clean reads after correcting the palindromes. In the Arabidopsis samples, 40–50% of reads contained at least one palindrome, with some reads containing up to 15 (Additional file 1). This demonstrates the extent to which palindromes pose a problem in PacBio WGA data and illustrates that Pacasus effectively detects and corrects these.

**Table 1:** Effect of correcting palindromes

Sample	Before cleaning		Reads with detectable palindromes		After correcting	
	Number of reads	Average length (b)	Number of reads	Number of reads (%)	Number of reads	Average length (b)
Ath-WGA1	462,138	9326	221,001	47.8	869,826	4660
Ath-WGA2	447,364	8544	195,263	43.6	769,027	4721
Ath-Ctrl	940,162	5680	4714	0.5	938,196	5681
GorY-WGA	3,596,236	5468	426,188	11.8	4,546,488	4234

Effect of correcting palindromes on the number reads and average lengths of these reads. Note: the Ath-Ctrl shows a small increase in average read length after correction and a lower number of reads. This is because Pacasus removes very short reads from the output

Table 1 shows that Pacasus decreases the average read length of the *A. thaliana* set by about 48%, i.e. preserving much of the long-range information. In the gorilla read set, 11.8% of the reads contain palindromes, less than in the *A. thaliana* sets. The average length of the gorilla reads before processing with Pacasus is 5468b, i.e. 61.2% of the average length in the total Ath-WGA data set (8934b); after correcting the palindromes this is increased to 90.3%: 4234b compared to 4689b. Pacasus finds palindromes in only in 0.5% of the reads in the non-amplified control data set, Ath-Ctrl. These reads will be a mixture of false-positives and missed/missing SMRTBell adaptors, which also cause palindromic sequences. The low number of palindromes found in the control set by Pacasus means that there is no need to perform subsequent analyses on ‘Ath-Ctrl-Clean’: the resulting de novo assembly for example will not be different from the one based on the original ‘Ath-Ctrl’ set.

**Fig. 2:**
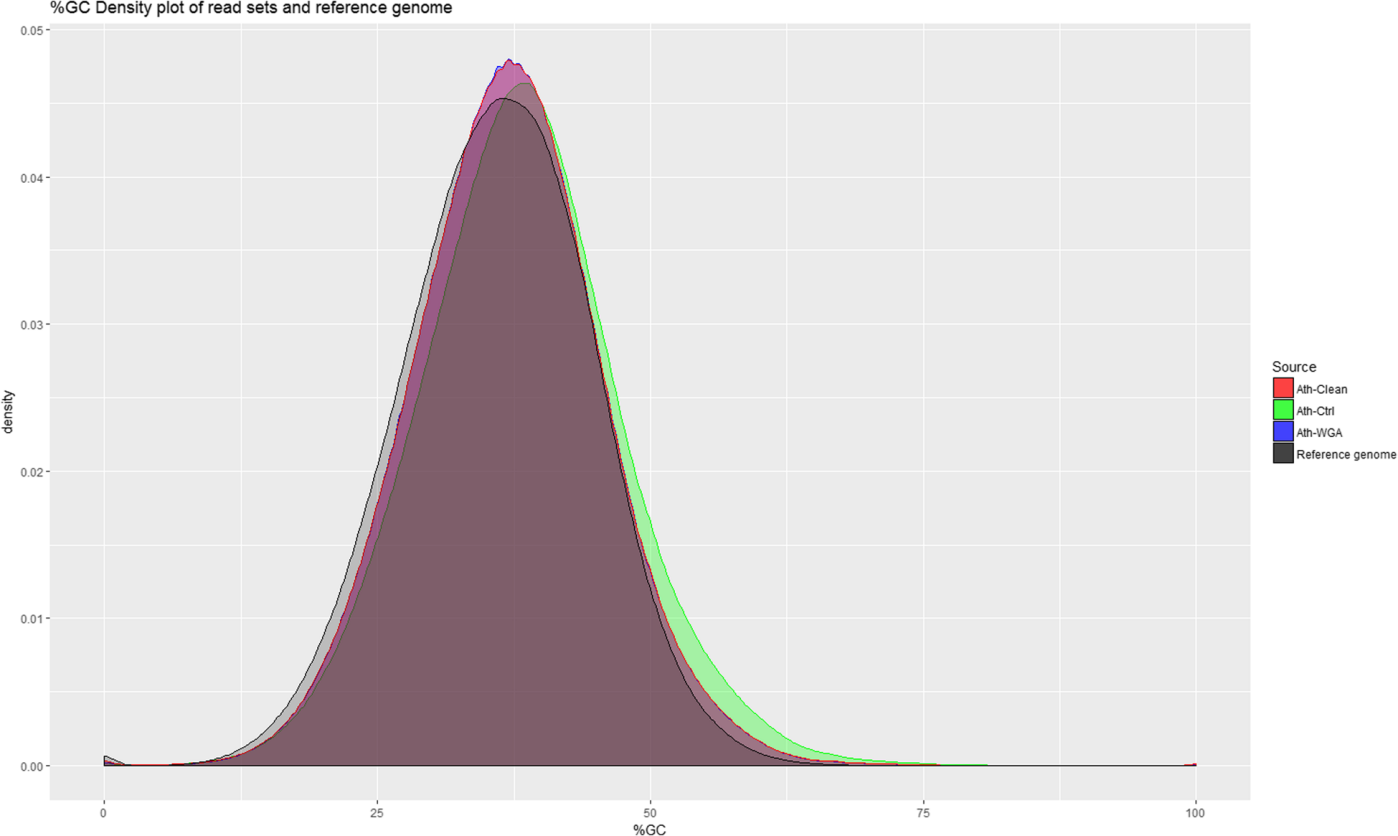
%GC density plot of Ath-Ctrl (green), Ath-WGA (blue), Ath-Clean (red) and the *A. thaliana* reference genome (black). The curves for Ath-WGA and Ath-Clean overlap completely. All three read sets do no show biases towards a certain GC-content when compared to the reference genome

The GC contents of the read sets were compared to that of the *A. thaliana* reference genome and no biases were observed for both the amplification process and the palindrome detection by Pacasus (Fig. 2).

### Correcting palindromes improves read mapping

Using the BLASR default settings and an additional filter of at least 80% nucleotide identity between read and reference, both the raw and clean read sets map well (Table 2). Palindromic reads map partially, leaving a (potentially large) proportion of the reads unmapped. This becomes clear when only mappings are considered where 80% and 95% of the complete read can be aligned: mapping efficiency for the raw read set drops from 99 to 44% and finally to 34%. For the clean reads, the mapping rates are 99%, 81% and 66% respectively, higher than for the noWGA read set (95%, 72% and 57%). Average coverages show similar effects. These mappings statistics indicate that the clean reads map more accurately and with higher read coverage than the raw reads do. The complete mapping reports are presented in Additional files 2, 3, 4.

**Table 2:** Read mapping statistics

	Reads mapped (%)			Avg. coverage			Avg. read length		
Alignment filter	–	80%	95%	–	80%	95%	–	80%	95%
Ath-WGA	99	44	34	40.5	17.4	13.4	8987	5568	5397
Ath-Clean	99	81	66	55.1	48.1	41.2	4690	4532	4697
Ath-Ctrl	95	72	57	34.2	29.2	24.8	5799	5583	5852

Statistics of read mappings with BLASR to the TAIR10 reference genome, calculated without filtering for a minimum read alignment length (‘-’) and after filtering for reads aligned with at least 80% or 95% nucleotide identity

To verify the palindromic nature of the reads, the locations of the clean reads were also investigated. If the raw reads indeed contain palindromic sequences, the parts of the clean reads should map to the same region in the genome (in contrast to chimeric reads, where the parts originate from different regions in the genome). To verify this, the longest distance between the mapping locations of each part of the corrected reads was calculated and related to the length of the original raw read. 96.5% of these mapping distances are within the read length of the original read, showing that most of the clean reads map to the same region in the genome and that the original raw reads indeed contain palindromes, not other types of chimeras.

### Assembly quality of corrected WGA reads approaches that of non-amplified data

To assess whether correcting palindromes also benefits assembly, we investigated two realistic scenarios using the *A. thaliana* data (Ath-WGA, Ath-Clean, Ath-Ctrl): PacBio-only assembly using Canu and a hybrid assembly, combining PacBio and Illumina HiSeq data, using DBG2OLC/Sparse. On the control data set Ath-Ctrl, this results in assemblies with overall good assembly statistics, with DBG2OLC yielding the best results (Table 3). Repeating the process with the original WGA data gives far worse results; the DBG2OLC assembly has, for example, an N50 value about 26-fold smaller than the N50 value of the control data and the assembly covers only about half (49.7%) of the reference genome.

**Table 3:** Statistics on the de novo assemblies

	PacBio-only (Canu)			Hybrid (DBG2OLC/Sparse)		
Read set	Ath-Ctrl (C1)	Ath-WGA (C2)	Ath-Clean (C3)	Ath-Ctrl (D1)	Ath-WGA (D2)	Ath-Clean (D3)
No. contigs	852	2128	1015	476	4818	1753
Ass. length (Mbp)	115.6	116.8	123.9	110.9	108.9	131.0
Longest contig (Kbp)	1181	655	3402	5667	246	2239
GC (%)	36.0	36.2	36.12	35.97	36.57	36.21
N50 (Kbp)	293	73	302	823	32	278
L50	117	479	109	33	951	113
Covered (%)	86.6	91.2	97.3	85.1	49.7	96.3
Dupl. ratio	1.09	1.07	1.06	1.08	1.34	1.13

Statistics on the PacBio-only and hybrid assemblies of the various datasets. Note that the TAIR10 reference genome is 119.7 Mb, with the full genome thought to be approximately 135 Mb [21]

Correcting the palindromic reads improves the hybrid assembly: although the N50 is lower than that of the Ath-Ctrl assembly, the assembly length and genome coverage are higher.

The Ath-Clean PacBio-only assembly is even better than the assembly based on the Ath-Ctrl data, with a higher N50 and genome coverage (Table 3). This is also reflected by the contig length distribution (Additional file 5). Apparently, the removal of conflicting information outweighs the loss of long-range information.

The hybrid assembly and the PacBio-only assembly based on Ath-Clean are longer than the TAIR10 reference genome (119.7 Mb), being 123.9 Mb and 131.0 Mb respectively. The full genome is thought to be approximately 135 Mb [21], so this additional sequence information could be new genomic data. No further testing has been done to verify this.

### De novo assembly based solely on long reads of flow-sorted chromosomes is now possible

**Fig. 3:**
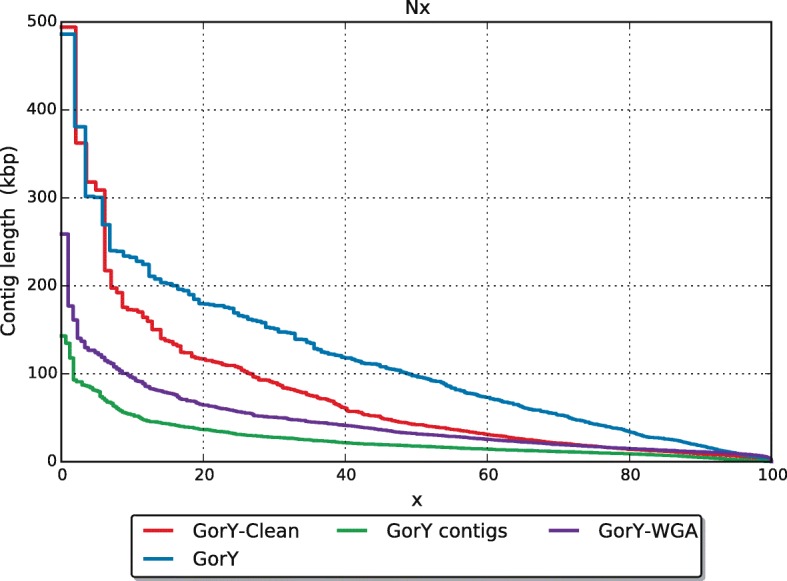
Contig length distributions. Contig length (y-axis) distribution of the published gorilla Y chromosome (GorY), the contigs underlying this assembly (GorY contigs), the de novo assembly based on raw PacBio data set (GorY-WGA) and of the de novo assembly of the cleaned reads (GorY-Clean). The x-axis shows the fraction of the assembly (e.g. the N20, N50, etcetera)

After correcting the palindromes in the original gorilla PacBio reads (see section “Discussion”.1) we were able to create two PacBio-only assemblies: GorY-WGA based on the raw data set and GorY-Clean, based on the clean reads. The GorY-WGA assembly was added to the comparison to stay in line with the *Arabidopsis thaliana* analyses described in the previous section and also to verify that the increase in quality is not only due to a better performing software application. Figure 3 shows the length distributions of both the contigs and the scaffolds in the previously published GorY hybrid assembly and the contigs in the new GorY-Clean / Gory-WGA PacBio-only assemblies. The GorY scaffolds were created by using long-range information to connect the contigs [4]. The scaffolds contain no additional information, except sequence contiguity. Gaps between contigs in the scaffolds are filled with Ns. The top-10 longest contigs of GorY-Clean are as long as the top-10 longest GorY scaffolds, showing that the new contigs already contain the same contiguity except that the gaps are filled with sequence information. The scaffolded GorY assembly seems larger than the GorY-Clean one (Table 4, Additional file 6). However, this is misleading as it contains 2.4 Mbp of Ns; the actual nucleotide content of the GorY assembly is 1.3 Mbp less than that of GorY-Clean. This is corroborated by further assembly statistics (Table 4). In terms of structure, the GorY-Clean assembly resembles the human Y chromosome assembly more than the original assembly (Additional file 7). The GorY-WGA assembly is also of higher quality compared to the GorY contigs, but not as good as the GorY-Clean assembly. We attribute the quality increase of GorY-WGA compared to the GorY assembly to the use of Canu [22]; the improvement of GorY-Clean over GorY-WGA is most likely due to correcting the palindromic reads with Pacasus.

**Table 4:** Human and gorilla Y-chromosome assembly statistics

	Assembly size (Mbp)	Ns (Mbp)	Non-Ns (Mbp)	No. sequences	N50 (Kbp)	Longest seq. (Kbp)
HumY	57.2	30.4	26.8	1		57,227
GorY, contigs	23.0	0	23.0	3001	18	143
GorY, scaffolds	25.4	2.4	23.0	697	98	486
GorY-WGA, contigs	26.5	0	26.5	1128	32	256
GorY-Clean, contigs	24.3	0	24.3	1062	42	494

Assembly statistics for the published human and gorilla Y chromosome assemblies and the new assemblies

The accuracy of the newly constructed GorY-Clean contigs becomes more apparent when looking at the read mapping statistics (Table 5). To calculate these, only contigs are used as these contain sequence information: reads will not map to Ns in the scaffolds. The gorilla Illumina HiSeq reads map better to the *human* reference genome (HumY) than to the original GorY assembly (in terms of genome coverage) and overall mapping accuracy is highest for both newly created assemblies. The GorY-Clean assembly is better covered by the read data than the GorY-WGA assembly is, regardless of whether corrected or non-corrected reads are used for evaluation. These results indicate that, apparently, currently available assemblers (in our case Canu) are better at handling chimeric reads than previous software and that the newly created assemblies (GorY-WGA and GorY-Clean) are more accurate than GorY.

**Table 5:** Read mapping statistics on the human and different GorY assemblies

		HiSeq			GorY-WGA			GorY-Clean		
Assembly	Length (Mbp)	Genome coverage		Read cov.	Genome coverage		Read cov.	Genome coverage		Read cov.
		(Mbp)	(%)		(Mbp)	(%)		(Mbp)	(%)	
HumY	26.8	22.3	83	1897	18.2	68	58.87	18.5	69	74.84
GorY	23.0	18.3	80	1169	21.1	92	71.33	21.1	92	99.15
GorY-WGA	26.5	24.9	94	1353	26.5	100	73.15	26.5	100	97.08
GorY-Clean	24.3	22.4	92	1586	24.3	100	83.21	24.3	100	109.67

Mapping of HiSeq, PacBio WGA and PacBio cleaned reads on the human Y chromosome (HumY), the gorilla Y chromosome (GorY) and the newly created gorilla Y assemblies (GorY-WGA, GorY-Clean). The read coverage is the average number of reads that a nucleotide has aligned to it

The average coverage when using the raw reads increased from 73.15x to 83.21x for the GorY-WGA and GorY-Clean respectively and with the corrected reads from 97.08x to 109.67x. These results show that the de novo GorY-Clean assembly fits the read data best and, as seen with the *Arabidopsis thaliana* data, mapping accuracy increases after correcting the palindromic reads.

### Resolving artificial duplications provides a higher coverage of genes on the Gorilla Y chromosome

The gorilla Y chromosome contains 12 single-copy X-degenerate genes [23]. To evaluate completeness of these genes in the assemblies, their corresponding transcript sequences were mapped to the GorY and GorY-Clean assemblies using the mRNA aligner GMAP [24]. The resulting alignments were subsequently used to identify the contigs/scaffolds that harbor these genes. For these 12 genes, the transcript coverage was on average higher in GorY-Clean contigs (84.9%) than in GorY scaffolds (74.9%), while sequence identity was similar (Additional files 8, 9). Additionally, the complete (exons and introns) sequences of the orthologous genes in the human genome (GRCh38) were aligned to the contigs/scaffolds harboring these genes in the GorY and GorY-Clean assemblies. Visual inspection of the dotplots (Additional files 10, 11, 12, 13, 14, 15) identified fewer inversions and duplications in the GorY-Clean contigs than in the GorY scaffolds (Additional file 16). In the alignment of the contigs from the GorY-Clean to the GorY scaffolds containing the same genes (Additional files 17, 18, 19, 20, 21, 22), no inverted duplications were detected in the GorY-Clean sequences. In contrast, numerous inverted duplications were visible in GorY sequences (Additional file 23). Thus, many inverted duplications were resolved in the assembly generated from the sequencing reads corrected by Pacasus, suggesting that such duplications indeed are an artefact of WGA.

### Effects on repeats and palindromic sequences of biological origin

DNA sequences are known to contain many different types and families of repeat sequences [25], including palindrome sequences [26]. These repeat sequences are present in the long reads after sequencing and can be detected by Pacasus as false positive palindromic sequences. When all repeats of biological origin are split, the subsequent de novo assembly will contain only collapsed regions effectively removing the repeats from the assembly. However, we speculate that most if not all of the true repetitive sequence are contained in sufficiently long reads to cover the repeats and therefore will not be considered by Pacasus for correction. In real data sets it is not known a priori which reads contain the true repetitive sequences, but evaluation is possible after performing the de novo assemblies: for both the *A. thaliana* and the gorilla Y chromosome the repeat content is known.

Table 6 shows the by RepeatMasker [27] detected repeat content of the *A. thaliana* assembly, including the reference assembly TAIR10. The repeat content of the assembly based on Ath-Clean (C3) is close to that of the reference and is higher than found in the assemblies based on Ath-Ctrl (C1) and Ath-WGA (C2), which is in line with the overall genome assembly statistics (Table 3).

**Table 6:** Repeat content

Assembly	Overall repeat content (% of assembly)
Canu Ath-Ctrl (C1)	15.73
Canu Ath-WGA (C2)	15.49
Canu Ath-Clean (C3)	16.76
TAIR10	16.88

Repeat content found by RepeatMasker in the different *A. thaliana* assemblies

**Fig. 4:**
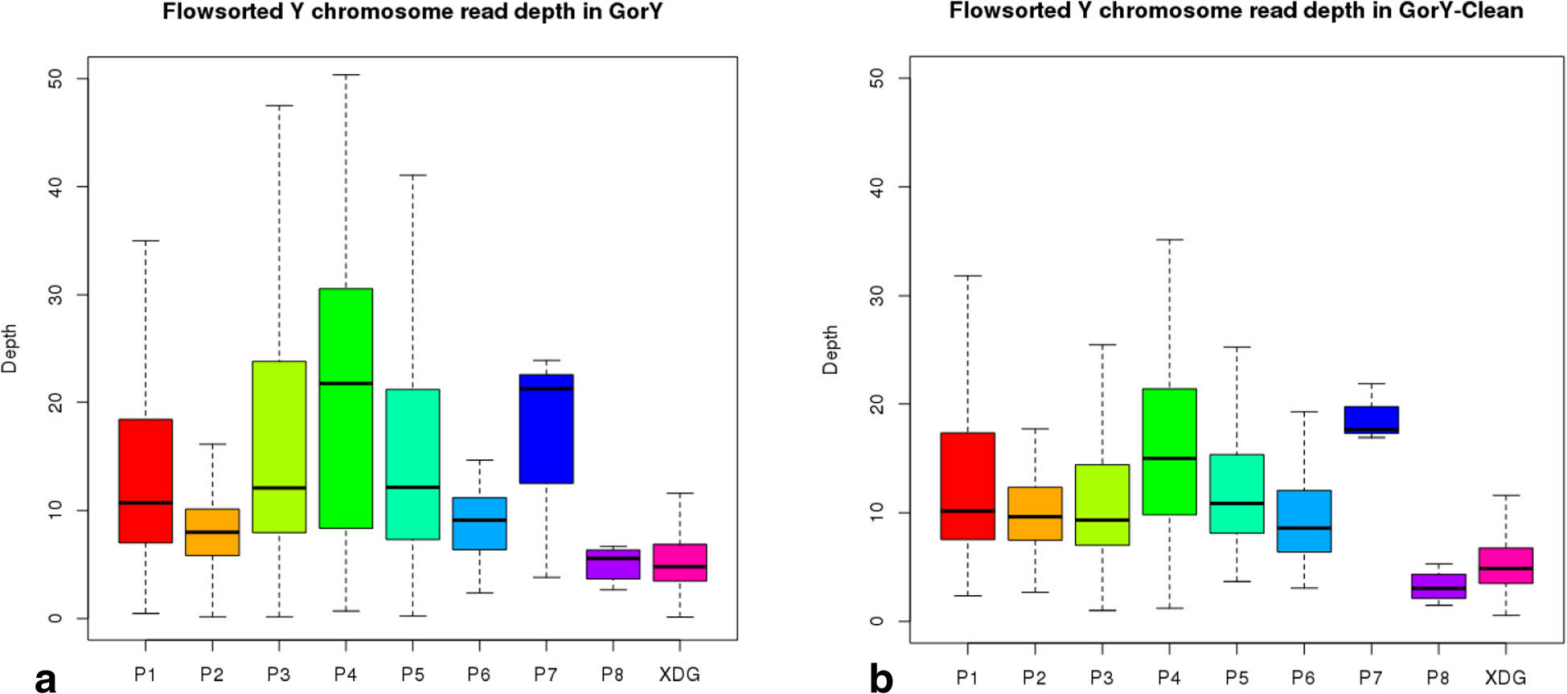
Illumina read depth of known palindromes. Illumina read depth of the known palindrome sequences P1-P8 and the X-degenerated gene (XDG) region in the GorY assembly (**a**) and GorY-Clean (**b**). Overal read depth is decrease in GorY-Clean, however in both assemblies the median read depths for P1-P7 are twice of that of XDG

To study the effect of Pacasus on actual biological palindromes present on the Y chromosome, 1-kb non-overlapping windows in GorY and GorY-Clean assemblies were identified which have high identity (> 80%) to well-defined human chromosome Y palindromes P1-P8 (arm lengths 8.7–1450 kb) [28] and X-degenerate gene (XDG) regions. Since the two arms of each palindrome have high identity, the windows representing the palindrome arms should have twice the read depth compared to the windows overlapping with the single-copy XDG regions on the Y chromosome. In order to obtain the read depth of these regions, Illumina-based flow-sorted gorilla Y paired end reads were aligned to GorY and GorY-clean assembly, and the read depth for the windows specific to palindrome regions and XDG regions were extracted. Figure 4a and b show that each palindrome is represented separately and Pacasus decreases the read depth for several palindromes, e.g., P3, P4, and P7. Nevertheless, the median read depth for each palindrome (except for P8) is still higher than XDG for both GorY and GorY-clean. This indicates that the biological palindromes are preserved by Pacasus.

## Discussion

After processing of the long reads, Pacasus has limited effect on the number of nucleotides in the read set and decreases the average read length by less than 50%. A downside of this process is that inverted repeats present in the genome will be treated as chimera, so that the repeat will be split into its separate elements, if the read does not span the full repeat. However, as shown in this manuscript, not all long reads suffer from chimeras. In most cases there will be sufficient reads long enough to cover the inverted repeat and palindrome sequences that are not split by Pacasus as shown in the assessment of the *A. thaliana* assemblies. It should be noted that Y chromosomes naturally possess non-artificial palindromes [28] and our analyses show that these palindromic sequences are also present in the de novo assembly after processing the reads by Pacasus.

Flow-sorted chromosomal DNA is usually contaminated with DNA from other chromosomes. Also with the gorilla sample, the original estimate is that approximately a third of the reads originate from the Y chromosome [4]. This is supported by our results, with 1,742,887 PacBio reads out of 4,546,488 reads mapping to the GorY-Clean assembly (38%). Consequently, some of the assembled contigs are not part of the gorilla Y chromosome but are from other chromosomes. Further analyses need to be performed to verify which contigs indeed originate from the Y chromosome. One suggestion is to look at read coverage: high coverage could point to Y chromosome sequences (see Additional file 7b). The next step to further improve the quality of the assembly could be to scaffold the contigs using the RNA-Seq data from the previous study [4] with for example SSPACE [29] and polish the final assembly with the HiSeq paired-end data using Pilon [30].

Tissue-specific analysis at the genome level is becoming more important in, for example, studying cancer genomes, but for genome assembly approaches they are currently limited to short-read sequencing [31] and hence result in more fragmented assemblies than is possible with long reads. For polyploid plant species is possible to select pollen and extract DNA from these cells, effectively decreasing the ploidy by half and therefor also decreasing the complexity of subsequent assembly process. And recent research shows that CRISPR-Cas9 introduces unwanted changes in the genome best detectable by long-read sequencing [32]. By combining WGA and Pacasus on these types of tissues it is now possible to isolate low amounts of DNA and to produce a high-quality genome to find these alterations in the genome.

A possible application not discussed in this paper is the detection of a SMRTbell adapter that is missed by the PacBio software pipeline, producing a raw read with the same structure as created with WGA. These incorrect reads, although perhaps present in low numbers, can have an impact on quality of the de novo assembly. When a non-WGA PacBio dataset with high genome coverage produces a fragmented assembly, it is worthwhile to run Pacasus on this dataset to correct the palindrome sequences created due to the missed SMRTbell adapter.

The detection of the palindrome sequences requires a full Smith-Waterman alignment due to the high error rate of the long-read technologies, which takes a considerable amount of compute power. Using high performance software and several GPUs we were able to process one SMRTcell per day, roughly keeping pace with sequencing speed of the PacBio RSII. The throughput of the PacBio Sequel is higher, hence processing these SMRTcells will require more time or compute resources, but we believe the results presented in this manuscript warrant the investment.

To find the location in the read at which it needs to be split, the backtrace part of the Smith-Waterman alignment algorithm needs to be performed [15]. In the current implementation of the PaSWAS module used for SW, the memory requirements are quadratic in the length of the read. For reads above 100 kb this memory requirement may limit the use of Pacasus. Currently the PacBio platforms generate reads below this length, but we expect the Oxford Nanopore platforms to go beyond this limit for at least some the reads in the near future. We will continue to work on Pacasus to decrease the memory requirements of the software to ensure that future output of sequencing platforms can be handled properly. The presented settings for the SW alignment are based on the error model of the RSII and our in-house experience with PacBio sequencing read qualities. For application on PacBio Sequel and Oxford Nanopore data, their respective error models may warrant minor changes to these settings.

## Conclusions

Whole-genome amplification is required for sequencing when a biological sample contains insufficient DNA for direct use in library preparation, but the process creates chimeric fragments. We have developed a new method, Pacasus, to correct long, error-rich reads containing such chimeras, based on high-speed Smith-Waterman alignment. We demonstrated the performance of Pacasus in terms of read mapping accuracy and assembly quality, showing that the loss in read length is clearly offset by the removal of incorrect contiguity information. On the Arabidopsis data, the hybrid assembly improves markedly in quality; and on the gorilla data, a PacBio-only assembly on clean reads is even of higher quality than a hybrid assembly including the original reads. The differences between the GorY-Clean and GorY-WGA assemblies are, however, not as large as in the *A. thaliana* case. The underlying reason for this is the much lower number of detectable palindromes in the gorilla read set: 11.8% of the reads contain palindromes, compared to 45.8% of the reads in the *A. thaliana* set. Correcting the relatively low number of reads containing palindromes in the gorilla data set already gave an improvement in assembly statistics, which indicates that the impact of these incorrect reads on the assembly quality is high. We expect that longer reads contain more palindromes, as indicated by the differences in average lengths before and after correcting in both examples.

In summary, Pacasus now allows to analyze PacBio data obtained from low amounts of DNA, making it possible to apply the power of long read technology to, for example, the study of single cells (e.g. in cancer research) and the study of single chromosomes (also in polyploid organisms).

## Methods

### The Pacasus algorithm

To detect chimeras created during WGA, raw PacBio reads are aligned to their reverse-complement sequence with Smith-Waterman (Fig. 1) using pyPaSWAS [16, 33]. The parameters used for alignment are: gap score, − 3; match score, 3; mismatch score, − 4. For filtering, the parameters are: filter factor, 0.01; query coverage, 0.01; query identity, 0.01; relative score, 0.01; and base score, 1.0. When no overlap is found in the alignment, the read is left intact and stored in the output file; otherwise the read is split at the center of alignment (see Fig. 1(d)). Both resulting fragments are again processed as if they were original reads, to allow the detection of nested palindrome sequences, until no overlap is detected anymore. If a fragment becomes shorter than a minimum length (default 50 bp) it is discarded. Note that the nucleotide information in the reads is neither removed nor changed. Pacasus is implemented in Python 2.7 and, besides pyPaSWAS (version > = 2.0), depends on Biopython [34] (version > = 1.67), numpy (http://www.numpy.org/) (version > = 1.8.0) and scipy [35] (version > = 0.12.0).

### Data for *Arabidopsis* evaluation

DNA was isolated from two *Arabidopsis thaliana* plants, labeled “Ath-WGA1” and “Ath-WGA2”, and amplified using the REPLI-g Mini Kit (QIAGEN Benelux BV, Venlo, The Netherlands). The *Arabidopsis thaliana* are in-house samples based on low-input plant materials (for more details on DNA isolation, library preparation and sequencing see [36]). Both samples were sequenced on both an Illumina HiSeq2000 sequencer and a PacBio RSII sequencer. A third DNA sample was used to generate PacBio RSII data without WGA (“Ath-Ctrl”). Table 7 shows the number of reads generated by each platform and for each sample. To evaluate mapping performance, PacBio reads were mapped to the TAIR10 *Arabidopsis thaliana Columbia* reference genome (http://www.arabidopsis.org) using BLASR version 1.3.1.124201 [37], and alignments with identity < 80% were filtered out by a Python script. Mapping reports were generated in CLCBio version 8.0.2 (http://www.clcbio.com).

**Table 7:** Datasets used for the performance analysis of Pacasus

Species	Sample	WGA	Illumina HiSeq2000		PacBio RSII	
			reads	length	Reads	avg. Length
*Arabidopsis thaliana*	Ath-WGA1	yes	31,233,196	100	462,138	9326
	Ath-WGA2	yes	43,810,780	100	447,364	8544
	Ath-Ctrl	no			940,162	5680
*Gorilla gorilla*	GorY-WGA	yes	279,601,852	150	3,596,236	5468

PacBio-only de novo assemblies of the *A. thaliana* genome were created using Canu version 1.3 [22]. Hybrid assemblies, combining the HiSeq2000 and RSII data, were created with DBG2OLC (released in 2016) [38]. DBG2OLC requires as input a HiSeq-only assembly; which we created using Sparse (released in 2015) [39], as recommended on the website by the authors of DGB2OLC, based on the HiSeq data from the WGA samples in all cases. The assembly was finalized with the PacBio reads.

We combined Ath-WGA1 and Ath-WGA2 into a single set, Ath-WGA, and created assemblies combining the HiSeq contigs with the raw Ath-WGA reads, with corrected (or ‘clean’) Ath-WGA reads (Ath-Clean) and with Ath-Ctrl reads. To evaluate quality, assemblies were compared to the reference genome using QUAST version 4.3 [40].

### Data for the gorilla Y chromosome evaluation

PacBio RSII data of a flow-sorted and amplified gorilla Y chromosome, GorY-WGA (Table 1), was downloaded from the NCBI Short Read Archive (SRA SRX1161235). The previously published assembly of the gorilla Y chromosome and the publicly available data from the flow-sorted, whole-genome amplified and de-branched gorilla Y chromosome, GorY [4], were downloaded as well (GCA_001484535.2). Canu version 1.3 was used for the assembly of the PacBio reads. For comparison, the human chromosome Y assembly (NC_000024.10), HumY, was downloaded. QUAST [40] was used for assembly comparison and statistics. PacBio reads were mapped to HumY, GorY and the new assemblies using BLASR (> 80% identity and > 80% read coverage); Illumina HiSeq 2500 PE reads (SRA SRR2176191) were mapped using CLCBio version 8.0.2. Statistics for all mapping results were calculated in CLCBio. For calculating the contig length distribution of the GorY assembly, scaffolds were broken up and N’s were removed. The gorilla X-degenerate gene transcripts were retrieved from a previous study [23]. GMAP version 2017-03-17 [24] was used to align the transcripts to the assemblies.

### Repeats and palindrome detection

RepeatMasker [27] was configured with rmblastn (2.2.27+) [41, 42] and RepBase (20140131) [43] for masking the *A. thaliana* assemblies and TAIR10 reference genome.

Following Tomaszkiewicz et al. [4], the gorilla Y contigs were broken into 1-kb windows and each window was aligned to human reference hg38 using lastz [44] (−-scores = human_primate.q, −-seed = match12, −-markend). RepeaMasker was also run on gorilla Y contigs to mask repeats and later for each window the total number of masked sites ‘N’ within a window were calculated. The windows which overlap with human Y chromosome palindromes and XDG genes were identified and filtered to make sure that they have at least 80% match with the human reference and less than 20% of N’s throughout the window.

BWA mem [45] was used to align the flow-sorted gorilla Y paired-end reads (SRX1160374) to the GorY-clean and GorY assemblies (unmasked). The bedtools [46] coverage function was used to calculate the read depth and coverage of each window. If the windows had > 80% coverage they were used to create boxplots within their respective palindromes. The boxplots were generated using R boxplot command with outline = TRUE parameter set.

The Human_primate.q file used for primate to primate alignments in lastz is as below:

$$ {\displaystyle \begin{array}{l}\mathrm{gap}\_\mathrm{open}\_\mathrm{penalty}\kern0.5em =\kern0.5em 500\#\mathrm{O}\\ {}\mathrm{gap}\_\mathrm{extend}\_\mathrm{penalty}=\kern0.75em 30\#\mathrm{E}\\ {}\mathrm{hsp}\_\mathrm{threshold}\kern1.25em =3000\#\mathrm{K}\\ {}\mathrm{gap}\mathrm{ped}\_\mathrm{threshold}\kern0.5em =4500\#\mathrm{L}\\ {}\mathrm{x}\_\mathrm{drop}\kern3em =\kern0.5em 900\#\mathrm{X}\\ {}\mathrm{y}\_\mathrm{drop}\kern3em =15000\#\mathrm{Y}\end{array}} $$

A C G T

$$ {\displaystyle \begin{array}{l}\mathrm{A}\kern0.5em 90\hbox{-} 330\hbox{-} 236\hbox{-} 356\\ {}\mathrm{C}\hbox{-} 330\ 100\hbox{-} 318\hbox{-} 236\\ {}\mathrm{G}\hbox{-} 236\hbox{-} 318\ 100\hbox{-} 330\\ {}\mathrm{T}\hbox{-} 356\hbox{-} 236\hbox{-} 330\kern0.5em 90\end{array}} $$

## Additional files

Additional file 1: Number of Pacasus iterations per read in the cleaned data set. (DOCX 40 kb)

Additional file 2: Mapping Report for Ath-Ctrl BLASR-mapping. (PDF 427 kb)

Additional file 3: Mapping Report for Ath-Clean BLASR-mapping. (PDF 418 kb)

Additional file 4: Mapping Report for Ath-WGA BLASR-mapping. (PDF 416 kb)

Additional file 5: Cumulative lengths of the PacBio-only and hybrid assemblies of Ath-Ctrl, Ath-WGA and Ath-Clean. (DOCX 114 kb)

Additional file 6: Cumulative length distribution of gorilla Y chromosome assemblies. (DOCX 264 kb)

Additional file 7: Mummerplots of HumY against the different GorY assemblies. (DOCX 166 kb)

Additional file 8: Coverage and identity of the X-degenerate gene transcripts in GorY and GorY-Clean assemblies. (DOCX 25 kb)

Additional file 9: Comparison of published GorY assembly to GorY-Clean in X-degenerate genes. (DOCX 34 kb)

Additional file 10: Dotplots mapping Human SRY gene to the GorY scaffold and GorY-Clean contig. (DOCX 39 kb)

Additional file 11: Dotplots mapping Human AMELY gene to the GorY scaffold and GorY-Clean contig. (DOCX 40 kb)

Additional file 12: Dotplots mapping Human NLGN4Y gene to the GorY scaffolds and GorY-Clean contigs. (DOCX 204 kb)

Additional file 13: Dotplots mapping Human PRKY gene to the GorY scaffolds and GorY-Clean contigs. (DOCX 228 kb)

Additional file 14: Dotplots mapping Human USP9Y gene to the GorY scaffolds and GorY-Clean contigs. (DOCX 186 kb)

Additional file 15: Dotplots mapping Human ZFY gene to the GorY scaffold and GorY-Clean contig. (DOCX 46 kb)

Additional file 16: The number of duplication or inversion events that were observed on contigs/scaffolds when aligned to human X-degenerate genes. (DOCX 26 kb)

Additional file 17: Dotplots mapping KDM5D gene containing GorY scaffold to GorY-Clean contig. (DOCX 39 kb)

Additional file 18: Dotplots mapping TBL1Y gene containing GorY scaffolds to GorY-Clean contigs. (DOCX 162 kb)

Additional file 19: Dotplots mapping USP9Y gene containing GorY scaffolds to GorY-Clean contigs. (DOCX 134 kb)

Additional file 20: Dotplots mapping SRY gene containing GorY scaffold to GorY-Clean contig. (DOCX 47 kb)

Additional file 21: Dotplots mapping TMSB4Y gene containing GorY scaffold to GorY-Clean contig. (DOCX 59 kb)

Additional file 22: Dotplots mapping DBY gene containing GorY scaffold to GorY-Clean contig. (DOCX 70 kb)

Additional file 23: The number of inverted duplication events visually observed on XDG-containing contigs/scaffolds when they are aligned to one another in a dotplot. (DOCX 26 kb)

## Abbreviations

Ath: *Arabidopsis thaliana*
GorY: *Gorilla gorilla* Y chromosome
HMW: High Molecular Weight
MP: Mate-pair
PE: Paired-End
WGA: Whole Genome Amplification
XDG: X-Degenerated Gene
