# Supplementary material for: Correcting palindromes in long reads after whole-genome amplification
Source: BMC Genomics. 2018 Nov 6;19:798. doi: 10.1186/s12864-018-5164-1 (PMC6218980; doi:10.1186/s12864-018-5164-1)
Supplement: Supplementary file 1 — Number of Pacasus iterations per read in the cleaned data set. (DOCX 40 kb) [file 12864_2018_5164_MOESM1_ESM.docx]

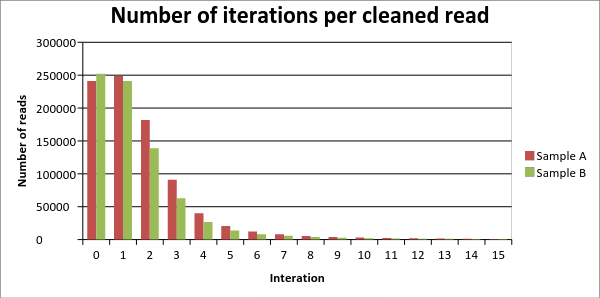


**Suppl. Figure 1.** Number of Pacasus iterations per read in the cleaned data set. Iteration 0 (I0) indicates that the original read had no detectable palindromic sequence. I1 indicates the read was processed only once, etc.
