# Supplementary material for: Correcting palindromes in long reads after whole-genome amplification
Source: BMC Genomics. 2018 Nov 6;19:798. doi: 10.1186/s12864-018-5164-1 (PMC6218980; doi:10.1186/s12864-018-5164-1)

Mapping Report for ath noWGA BLASR-mapping
Type: Read mapping
Reference count: 7
Generated by: warri004
Date: Thu Apr 28 01:08:24 CEST 2016
Software: CLC Genomics Server 7.5.1

Table of contents

1. Summary 3
2. References 3
    2.1 Reference coverage 3
    2.2 Coverage statistics 3
    2.3 Coverage level distribution 4
    2.4 Coverage level distribution (excl. zero coverage regions) 5
    2.5 Zero coverage regions 6
    2.6 Coverage vs GC Content Plot 6
3. Mapped reads 6
    3.1 All mapped reads 6
    3.2 Non-specific matches 7
    3.3 Non-perfect matches 8
    3.4 Read length distribution 11
    3.5 Insertion length distribution 12
    3.6 Deletion length distribution 13
    3.7 Nucleotide differences in reads relative to reference 13
    3.8 Nucleotide Mapping 13
    3.9 Quality for match distribution 17
    3.10 Quality for mismatch distribution 17
    3.11 Mismatch fraction per read position 18
    3.12 Unaligned ends 18

1. Summary

Reference count	7
Type	Read mapping
Total reference length	119,667,750
GC contents in %	36.00
Total read count	892,146
Mean read length	5,799.85
Total read length	5,174,315,491

2. References

2.1 Reference coverage

Total reference length	119,667,750
% GC	36.00
Total consensus length	-
Fraction of reference covered	0.97

2.2 Coverage statistics

Total reference length	119,667,750
Minimum coverage	0
Maximum coverage	12,116
Average coverage	34.24
Standard deviation	269.42
Minimum excl. zero coverage regions	1
Average excl. zero coverage regions	35.42
Standard deviation excl. zero coverage regions	274.06

2.3 Coverage level distribution

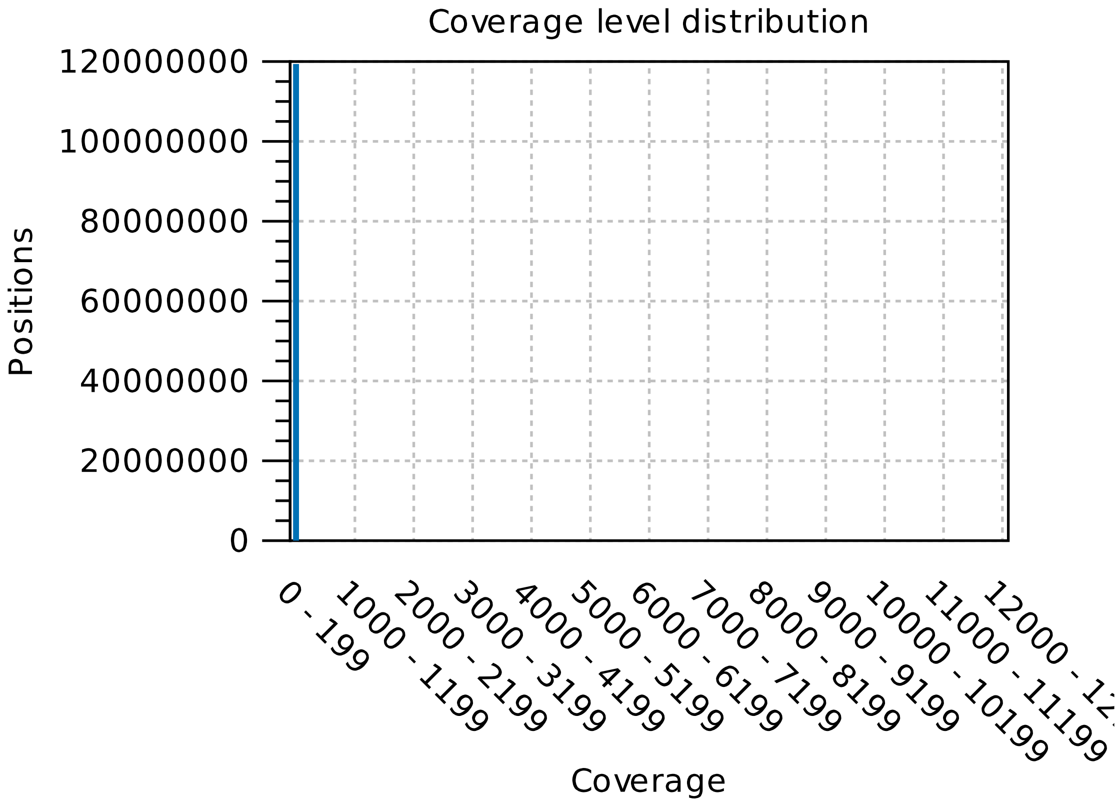

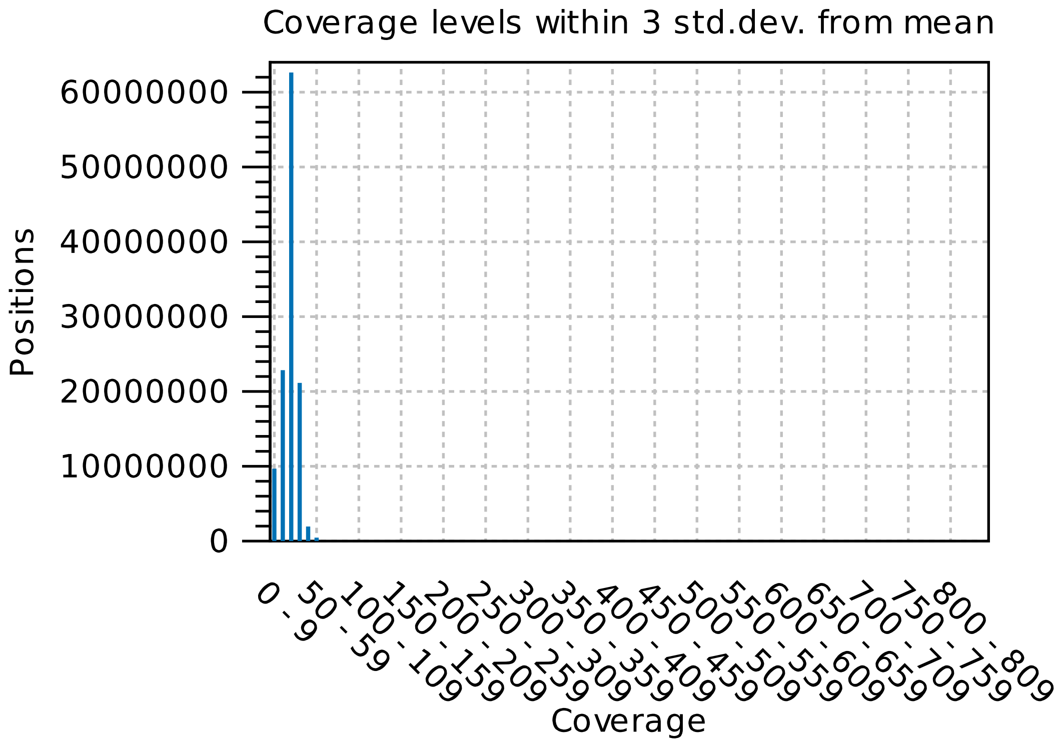


```
119,449,424 positions have coverage between 0 and 842.
218,326 positions have coverage above 842 (not shown in graph).
```

2.4 Coverage level distribution (excl. zero coverage regions)

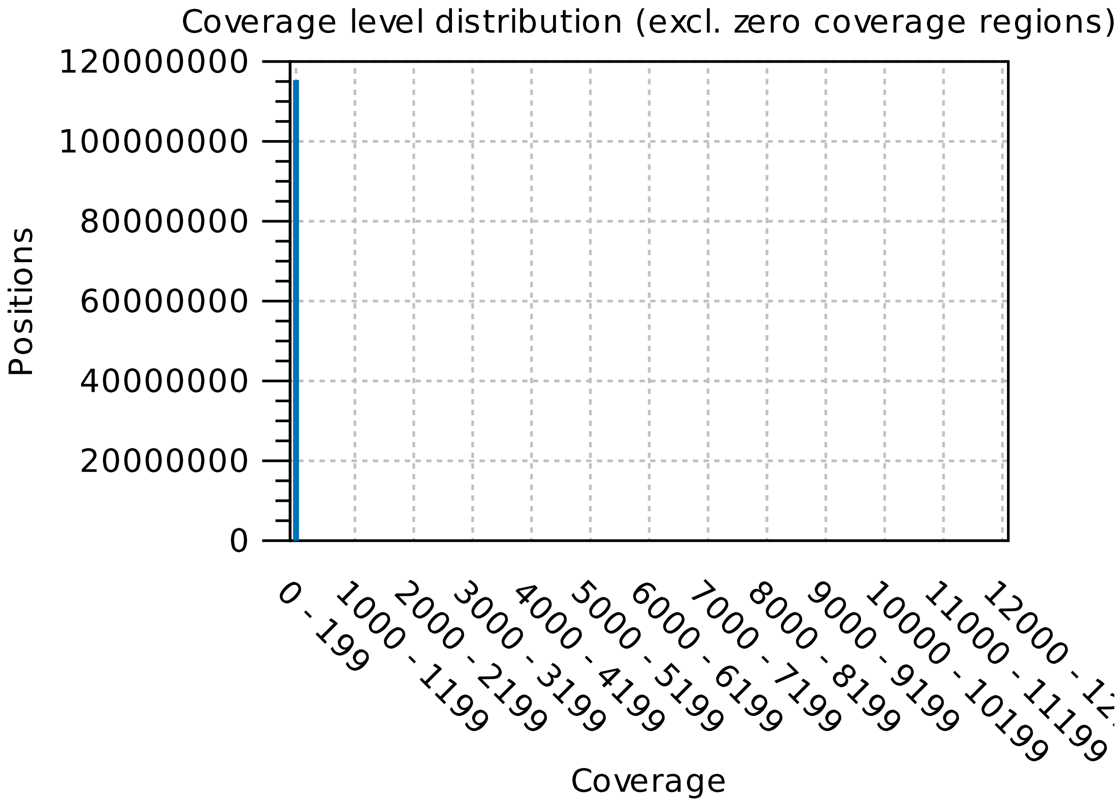


```
4,010,703 positions have zero coverage (not shown in graph).
```

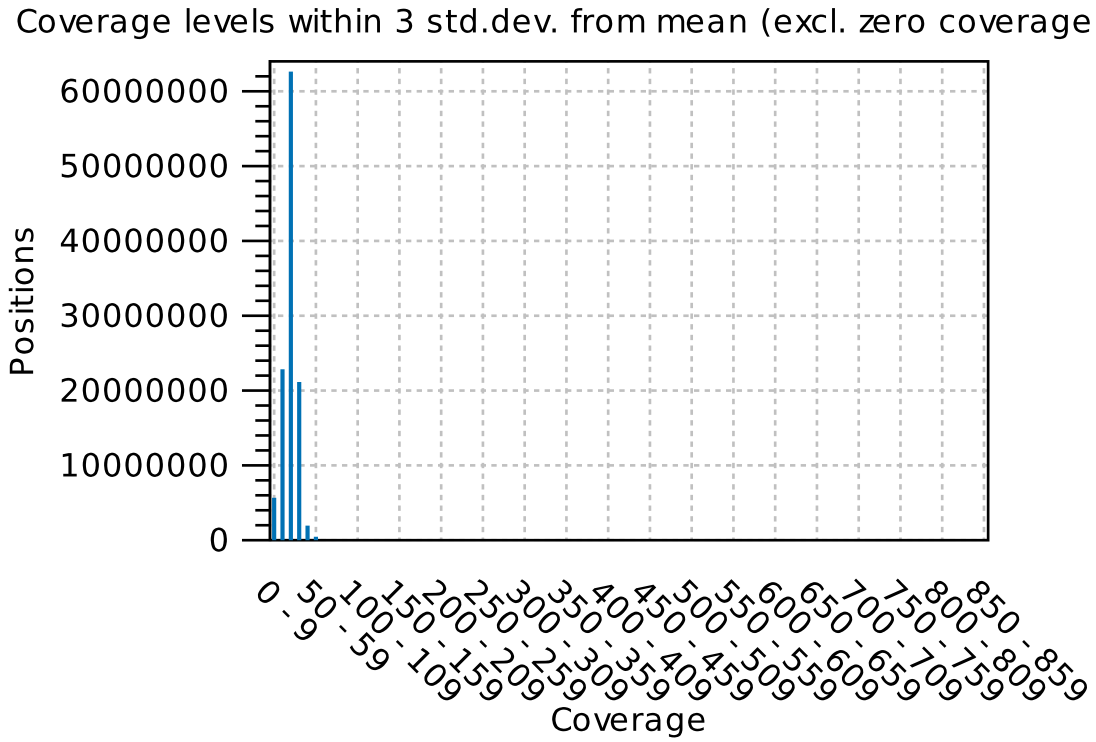


```
4,010,703 positions have coverage below 1 (not shown in graph).
115,439,386 positions have coverage between 1 and 857.
```

217,661 positions have coverage above 857 (not shown in graph).

2.5 Zero coverage regions

Count	2,161
Minimum length	1
Maximum length	53,746
Mean length	1,855.95
Standard deviation	3,237.71
Total length	4,010,703

2.6 Coverage vs GC Content Plot

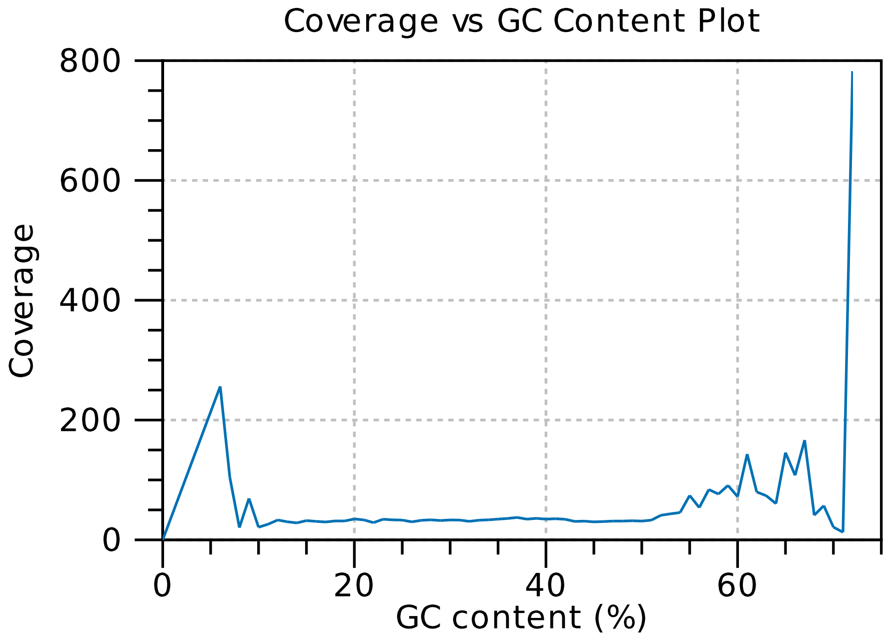

The plot displays, for each GC content level (0-100%), the mean read coverage of 100bp ref erence segments with that GC content.

3. Mapped reads

3.1 All mapped reads

Read count	892,146
Mean read length	5,799.85
Total read length	5,174,315,491

3.2 Non-specific matches

Non-specific matches

Read count	87,149
% of all mapped reads	9.77
Mean read length	5,368.25
Total read length	467,837,994

Distribution of match specificity (all matches)

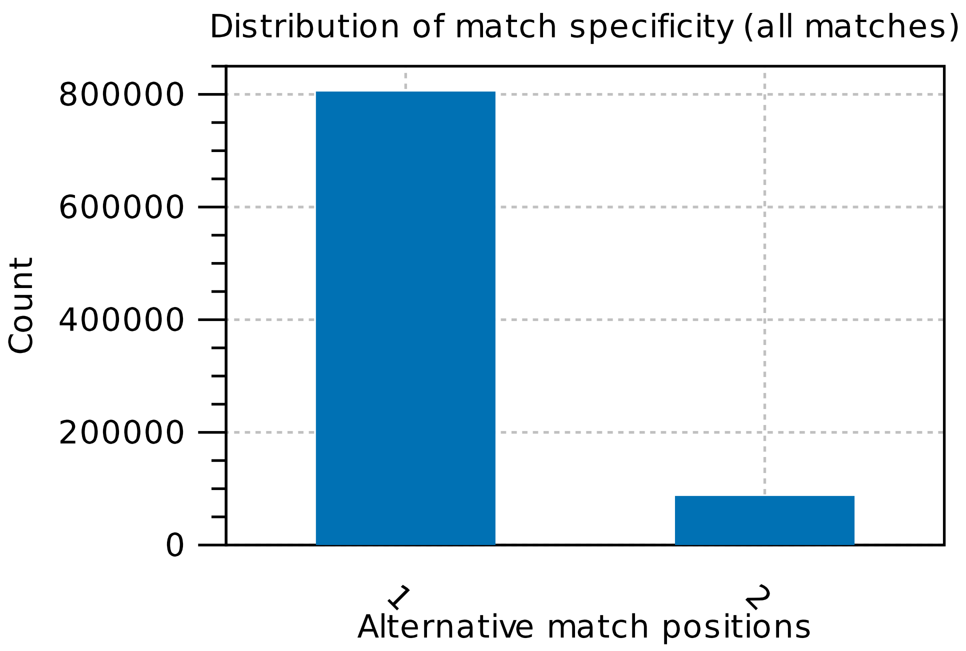

Distribution of match specificity (non-specific matches)

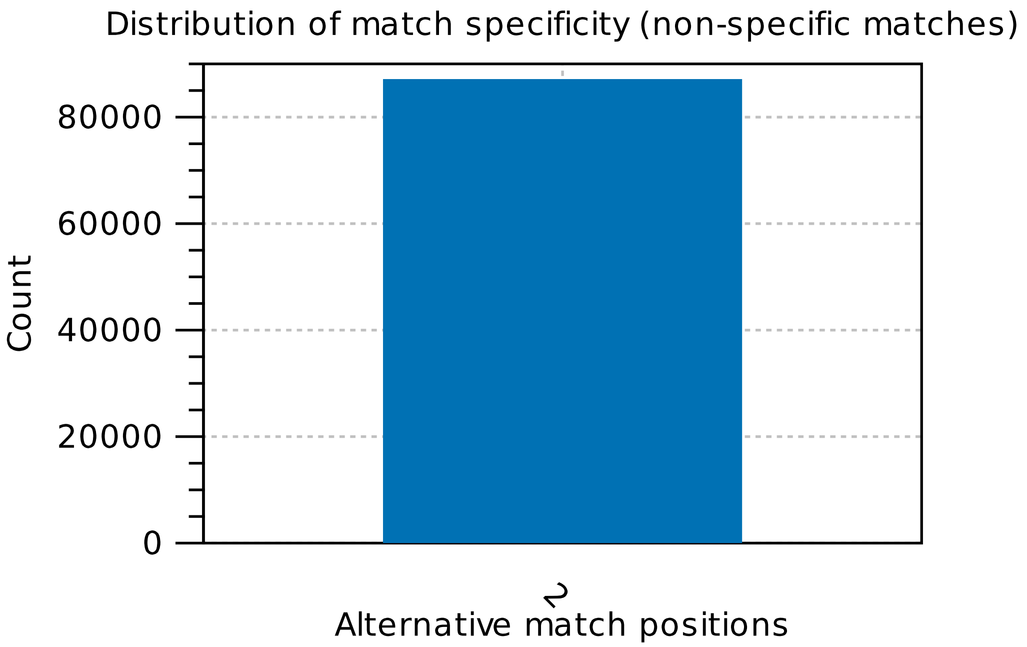

3.3 Non-perfect matches

Non-perfect matches

Read count	892,146
% of all mapped reads	100.00
Mean read length	5,799.85
Total read length	5,174,315,491

Distribution of mismatches (all matches)

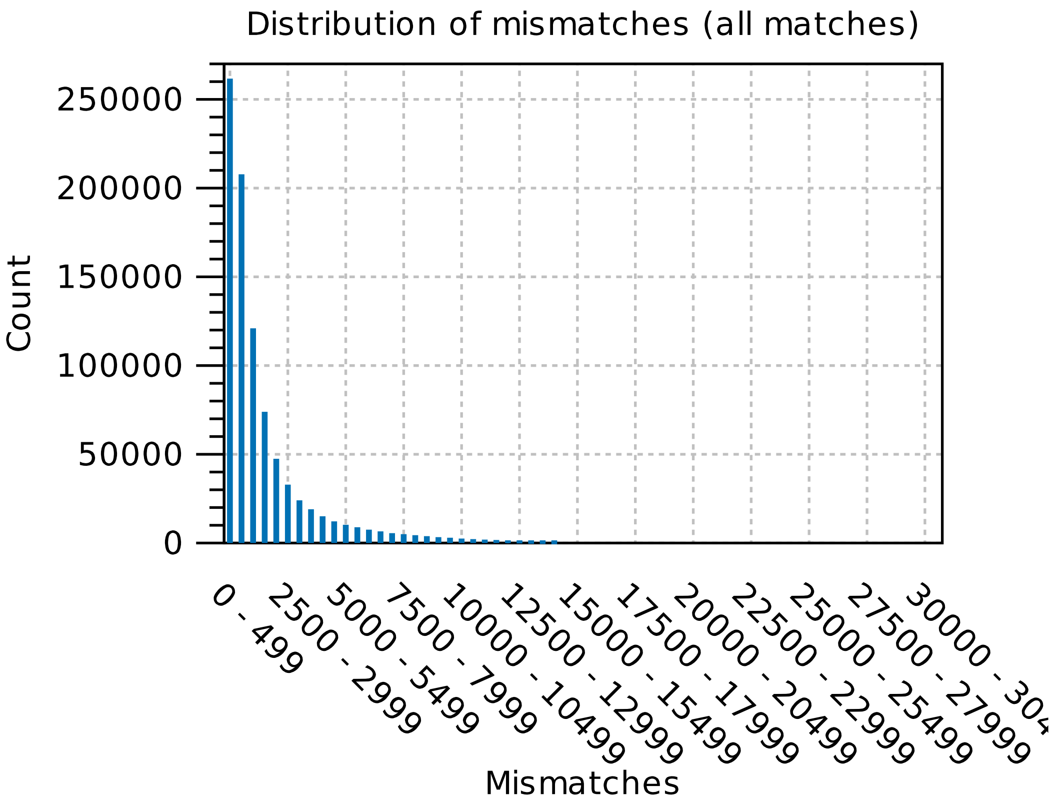

Distribution of mismatches (non-perfect matches)

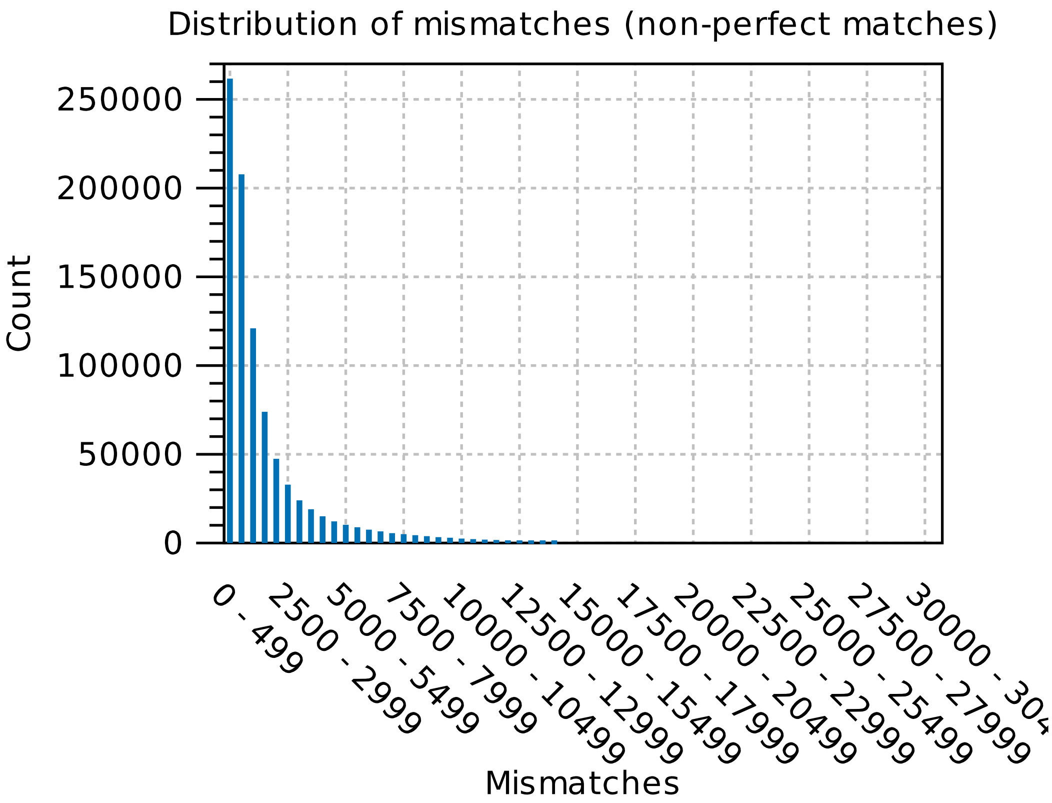

3.4 Read length distribution

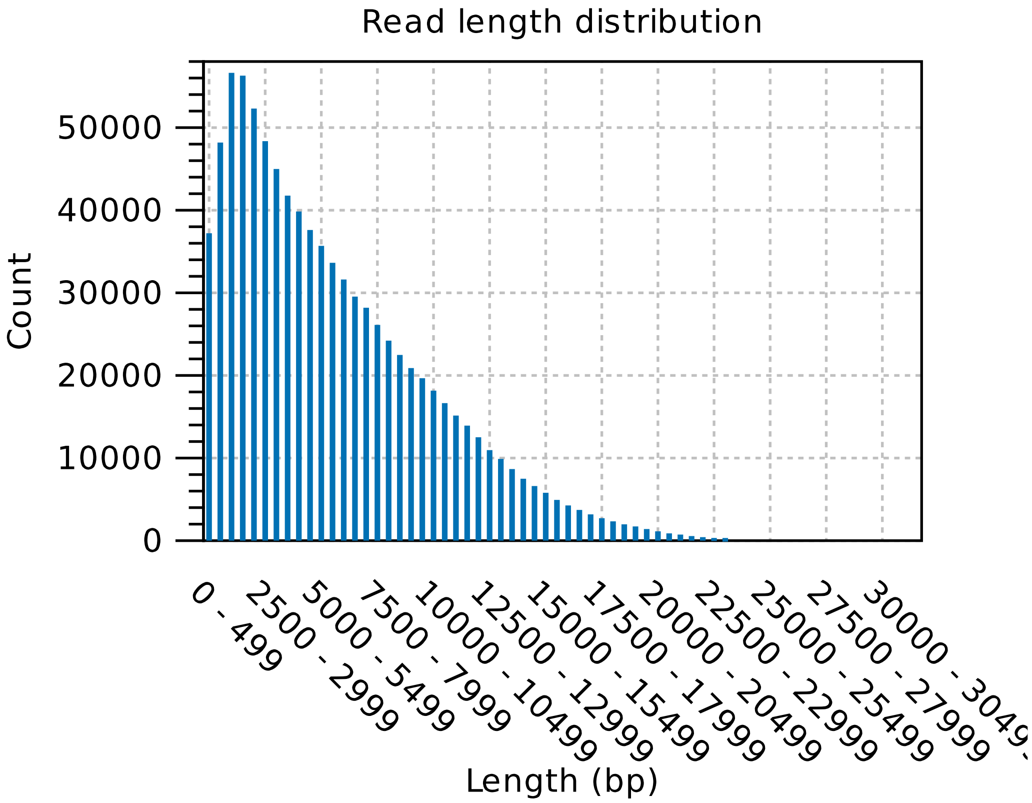

3.5 Insertion length distribution

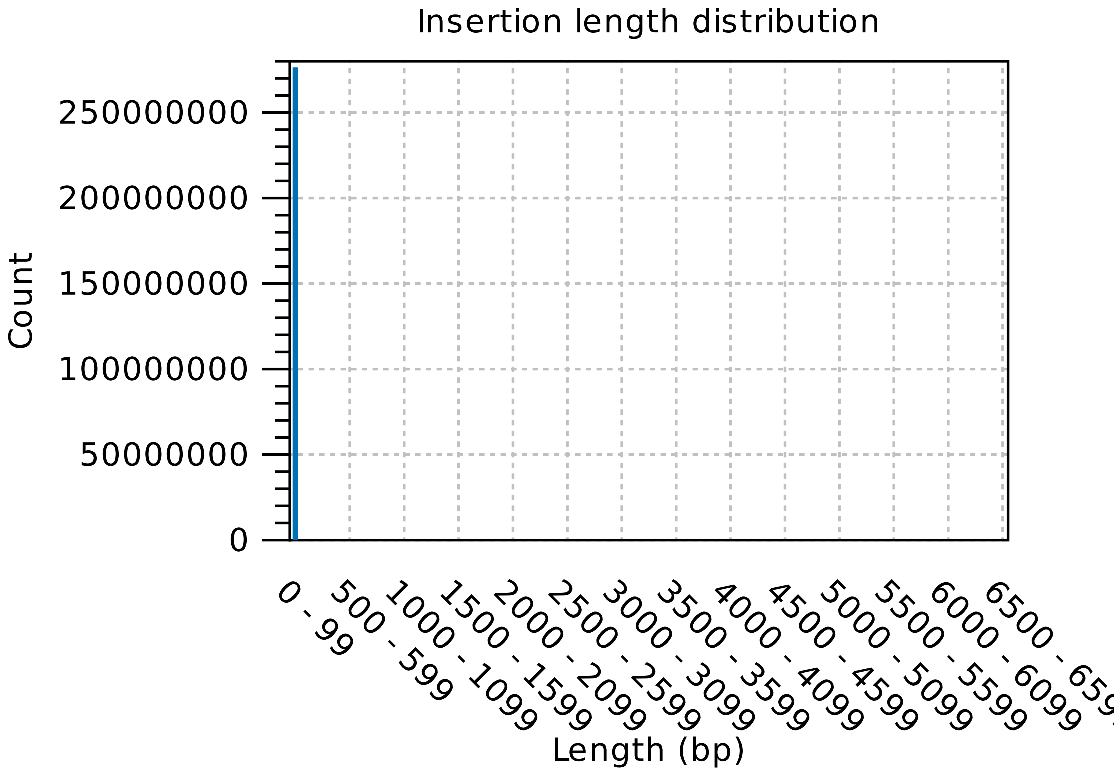

3.6 Deletion length distribution

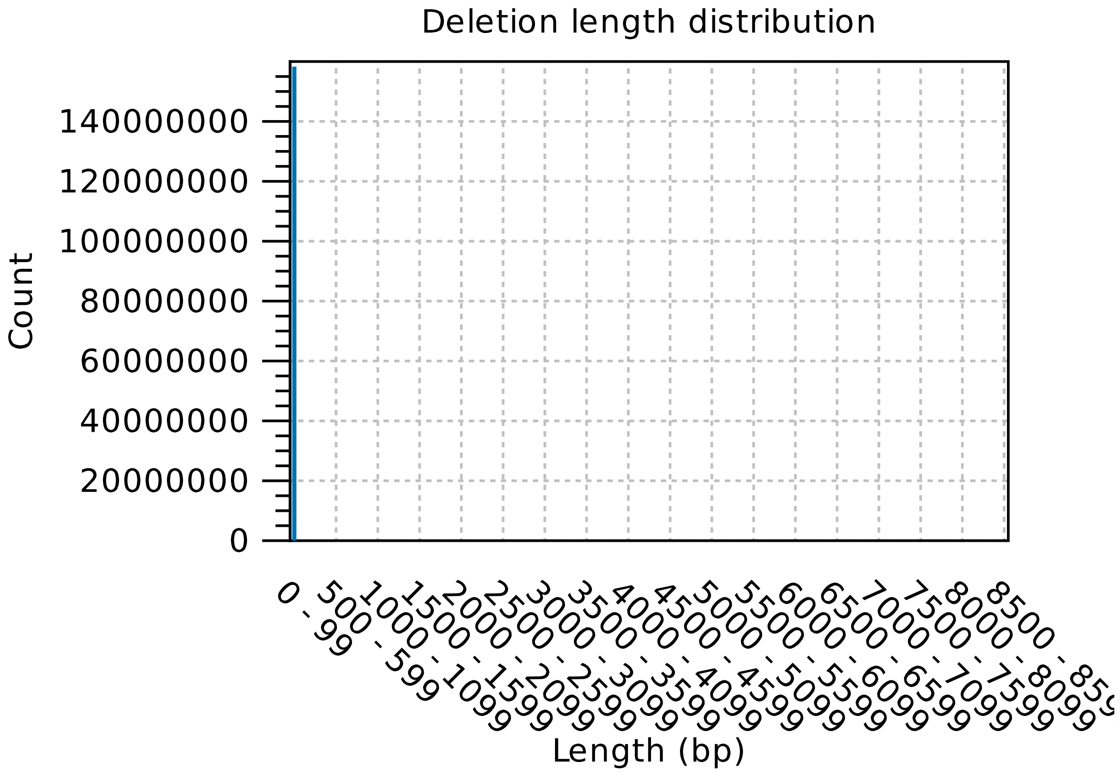

3.7 Nucleotide differences in reads relative to reference

Nucleotide in reference	% read bases that differ
A	7.17
C	8.15
G	8.04
T	7.09
-	0.93
Total	1.47

3.8 Nucleotide Mapping

Counts

Reference: A	1,202,144,018	7,759,241	7,953,231	6,563,728
Reference: C	5,941,656	689,013,261	4,026,505	5,668,535
Reference: G	5,483,486	3,825,558	687,209,514	6,169,006
Reference: T	6,514,541	7,678,559	8,036,654	1,211,970,081
Reference: -	101,516,930	107,320,946	111,431,706	103,650,238

Total	1,321,600,631	815,597,565	818,657,610	1,334,021,588

Read: -	Total
70,620,713	1,295,040,931
45,539,864	750,189,821
44,578,008	747,265,572
70,266,786	1,304,466,621
45,138,265,556	45,562,185,376
45,369,270,927	49,659,148,321

Percentages

Reference: A	2.42	0.02	0.02	0.01
Reference: C	0.01	1.39	0.01	0.01
Reference: G	0.01	0.01	1.38	0.01
Reference: T	0.01	0.02	0.02	2.44
Reference: -	0.20	0.22	0.22	0.21
Total	2.66	1.64	1.65	2.69

Read: -	Total
0.14	2.61
0.09	1.51
0.09	1.50
0.14	2.63
90.90	91.75
91.36	100.00

Relative errors (reference to read)

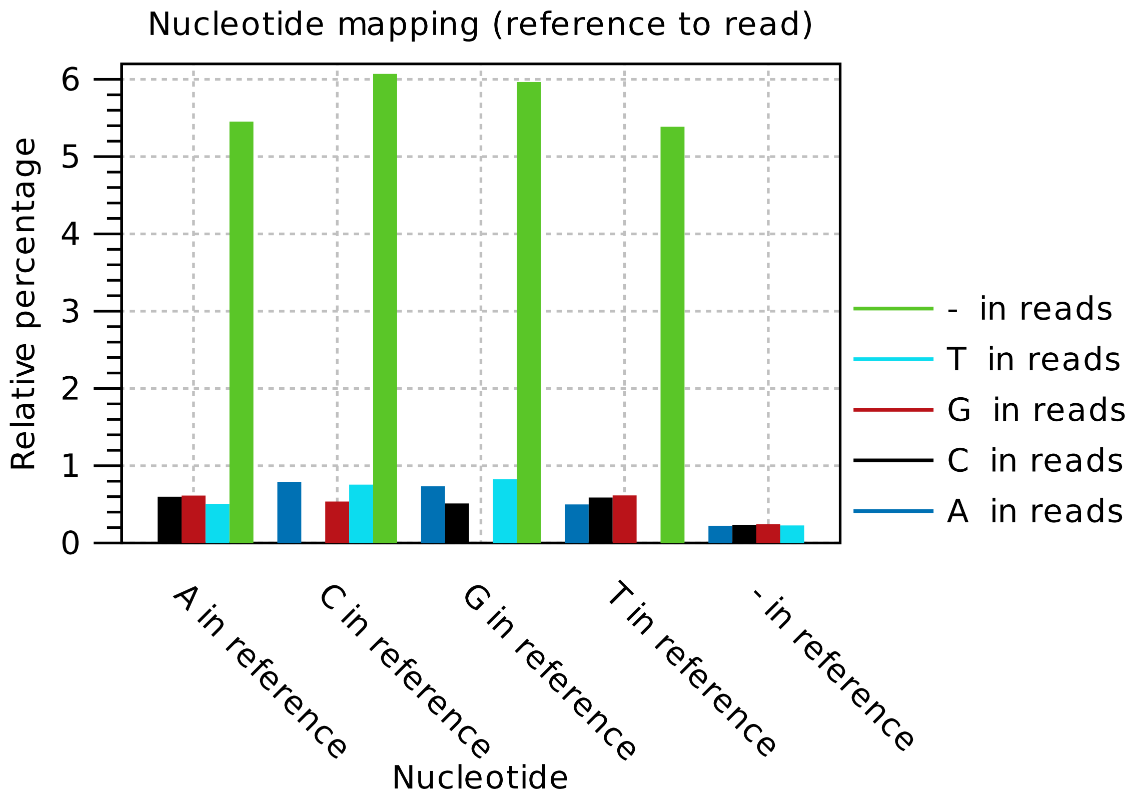

Bars depict off-diagonal element counts divided by counts in the column with header "total ".

Error counts (reference to read)

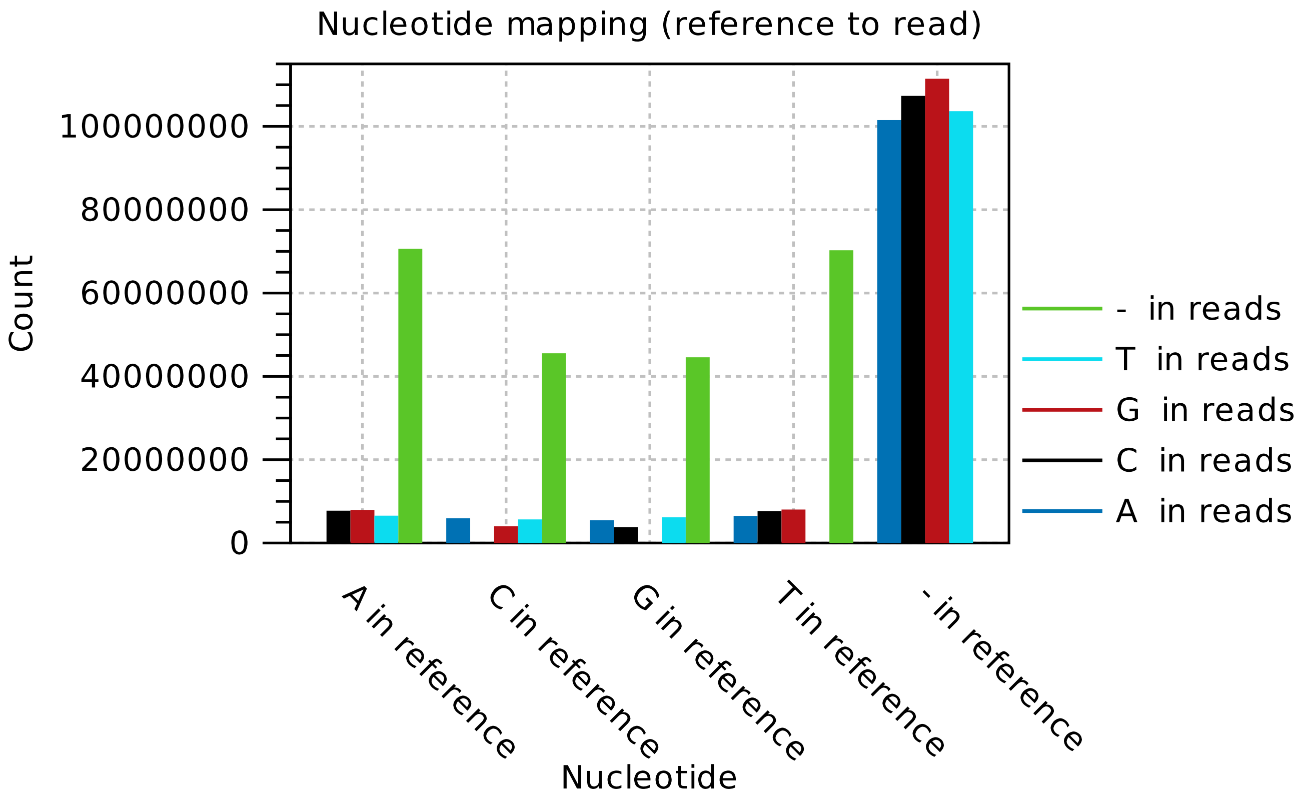

Bars depict off-diagonal element counts.

Relative errors (read to reference)

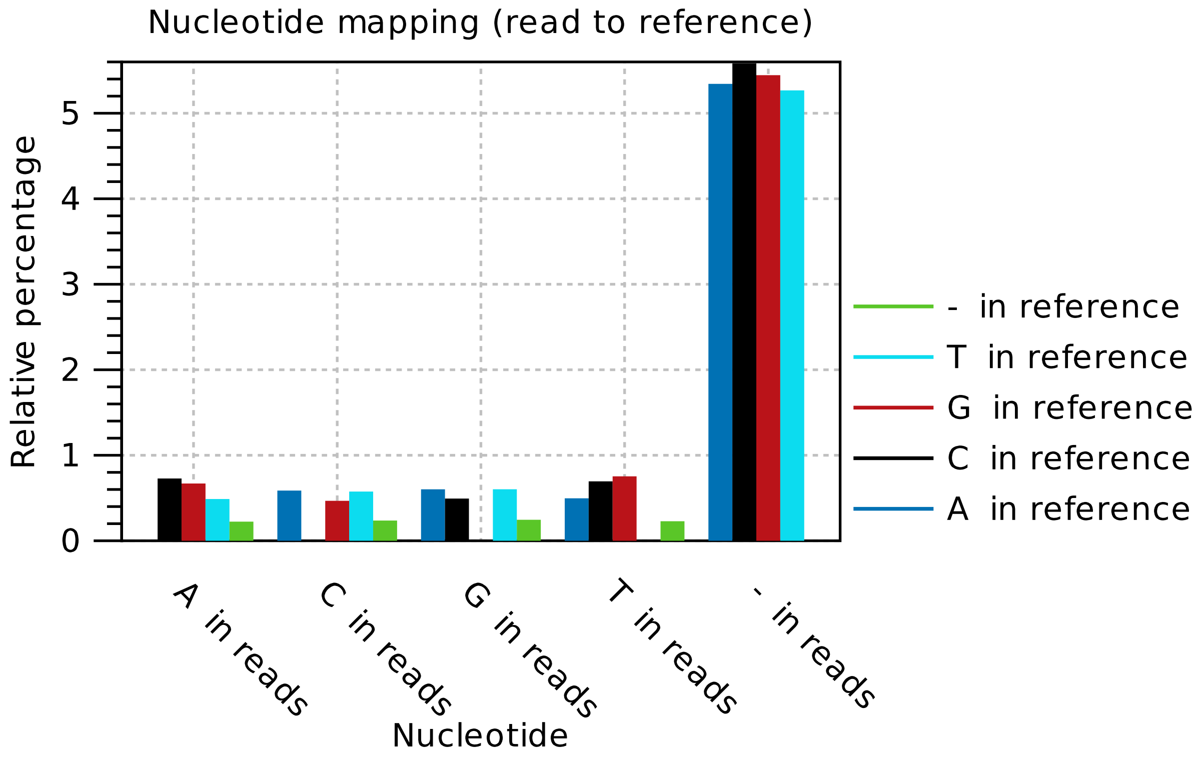

Bars depict off-diagonal element counts divided by counts in the row with header "total".

Error counts (read to reference)

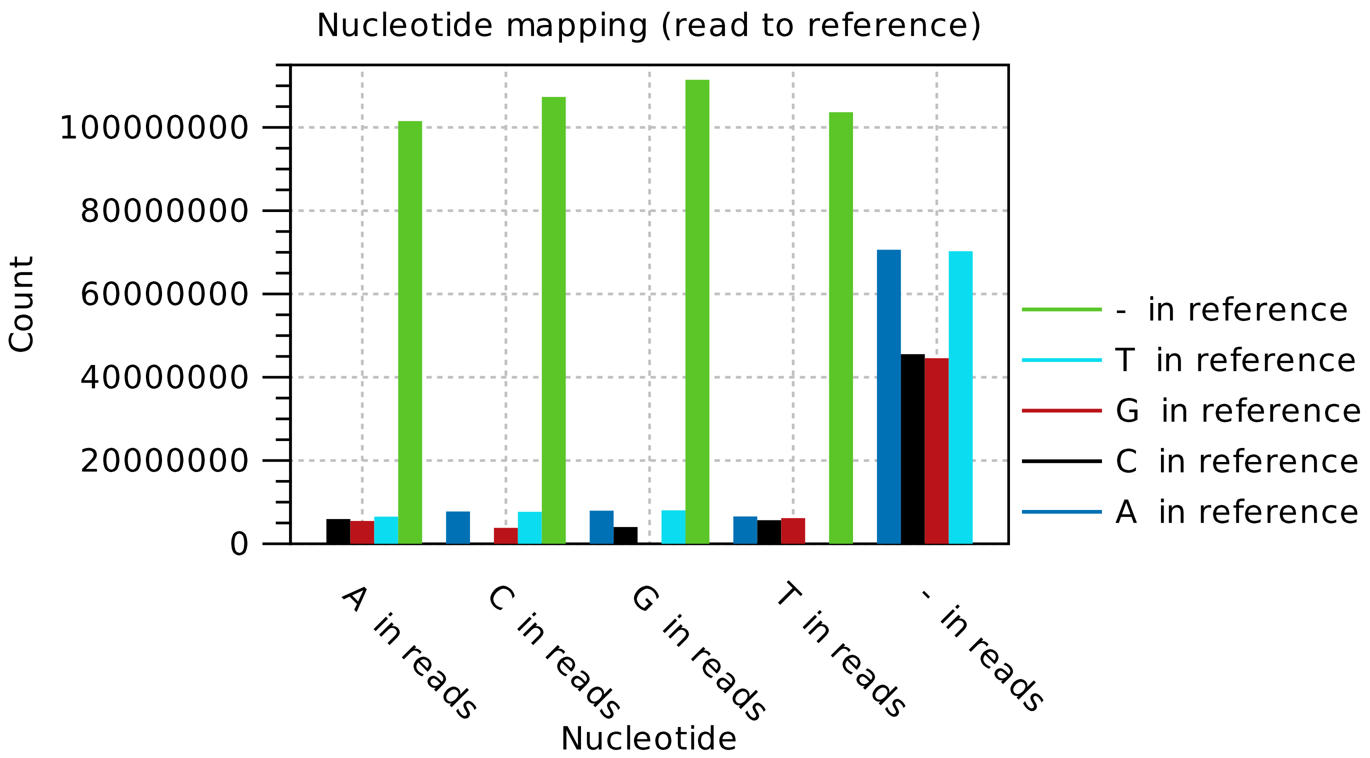

Bars depict off-diagonal element counts.

3.9 Quality for match distribution

There are no qualities.

3.10 Quality for mismatch distribution

There are no qualities.

3.11 Mismatch fraction per read position

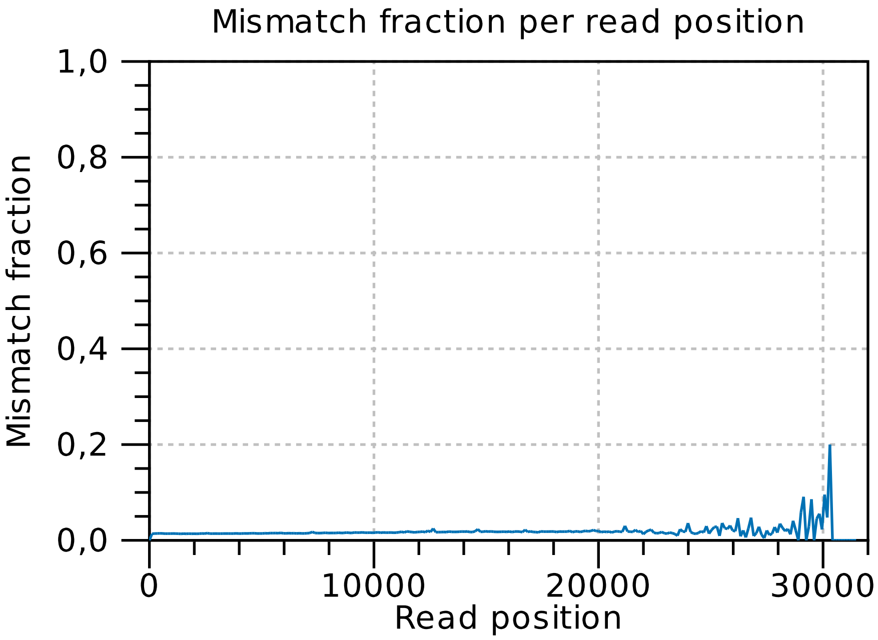

The plot displays the mismatch fraction per read position in the sequenced segments

3.12 Unaligned ends

Read count	1,490,774
% of all mapped reads	167.10
Positions covered	1,167,914
Positions covered in % of reference	0.98
Positions covered in % of bases covered	1.01

Reads with one unaligned end

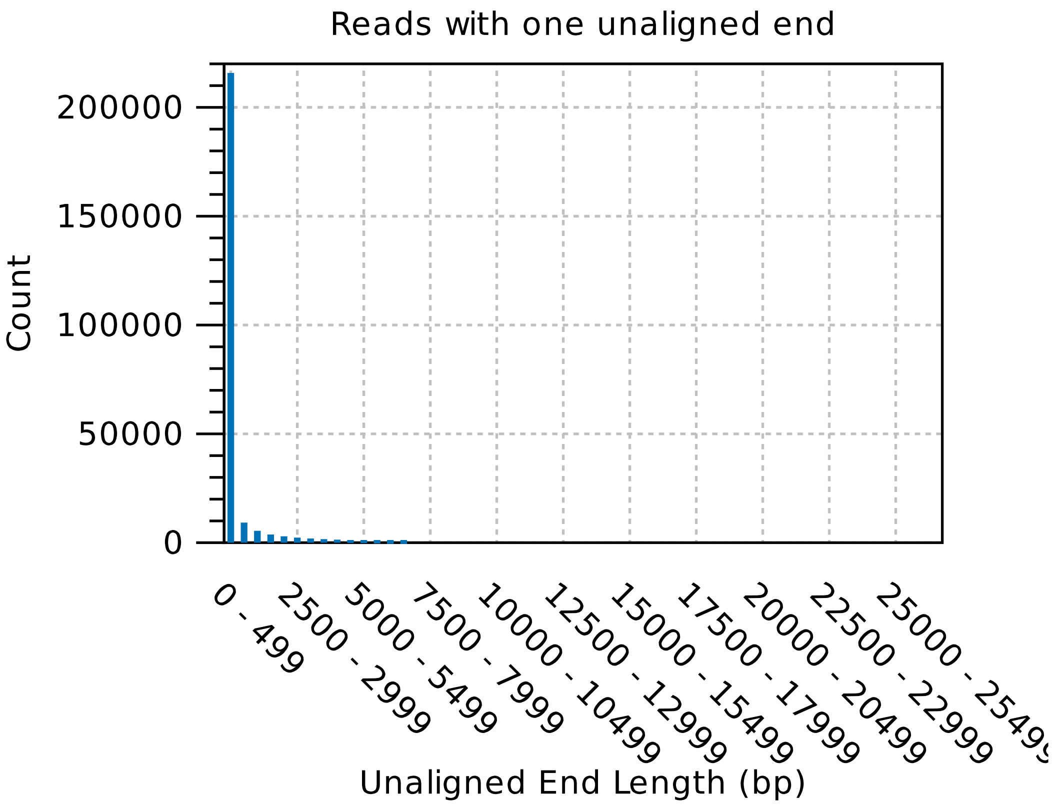

Reads with two unaligned ends

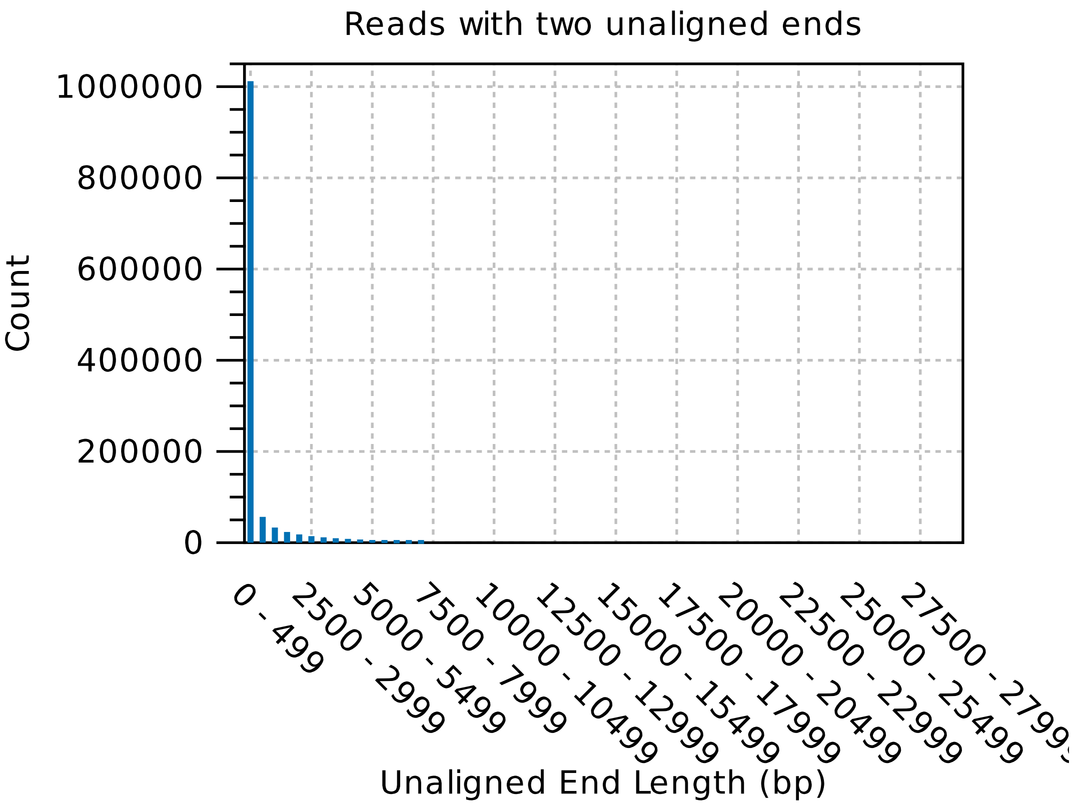

Supplement: Supplementary file 2 — Mapping Report for Ath-Ctrl BLASR-mapping. (PDF 427 kb) [file 12864_2018_5164_MOESM2_ESM.pdf]
