# Supplementary material for: Correcting palindromes in long reads after whole-genome amplification
Source: BMC Genomics. 2018 Nov 6;19:798. doi: 10.1186/s12864-018-5164-1 (PMC6218980; doi:10.1186/s12864-018-5164-1)

Mapping Report for ath.all.cleaned BLASR-mapping
Type: Read mapping
Reference count: 7
Generated by: warri004
Date: Thu Apr 21 15:57:13 CEST 2016
Software: CLC Genomics Server 7.5.1

Table of contents

1. Summary 3
2. References 3
    2.1 Reference coverage 3
    2.2 Coverage statistics 3
    2.3 Coverage level distribution 4
    2.4 Coverage level distribution (excl. zero coverage regions) 5
    2.5 Zero coverage regions 6
    2.6 Coverage vs GC Content Plot 6
3. Mapped reads 6
    3.1 All mapped reads 6
    3.2 Non-specific matches 7
    3.3 Non-perfect matches 8
    3.4 Read length distribution 11
    3.5 Insertion length distribution 12
    3.6 Deletion length distribution 13
    3.7 Nucleotide differences in reads relative to reference 13
    3.8 Nucleotide Mapping 13
    3.9 Quality for match distribution 17
    3.10 Quality for mismatch distribution 17
    3.11 Mismatch fraction per read position 18
    3.12 Unaligned ends 18

1. Summary

Reference count	7
Type	Read mapping
Total reference length	119,667,750
GC contents in %	36.00
Total read count	1,625,293
Mean read length	4,689.63
Total read length	7,622,015,789

2. References

2.1 Reference coverage

Total reference length	119,667,750
% GC	36.00
Total consensus length	-
Fraction of reference covered	1.00

2.2 Coverage statistics

Total reference length	119,667,750
Minimum coverage	0
Maximum coverage	12,626
Average coverage	55.05
Standard deviation	185.62
Minimum excl. zero coverage regions	1
Average excl. zero coverage regions	55.16
Standard deviation excl. zero coverage regions	185.80

2.3 Coverage level distribution

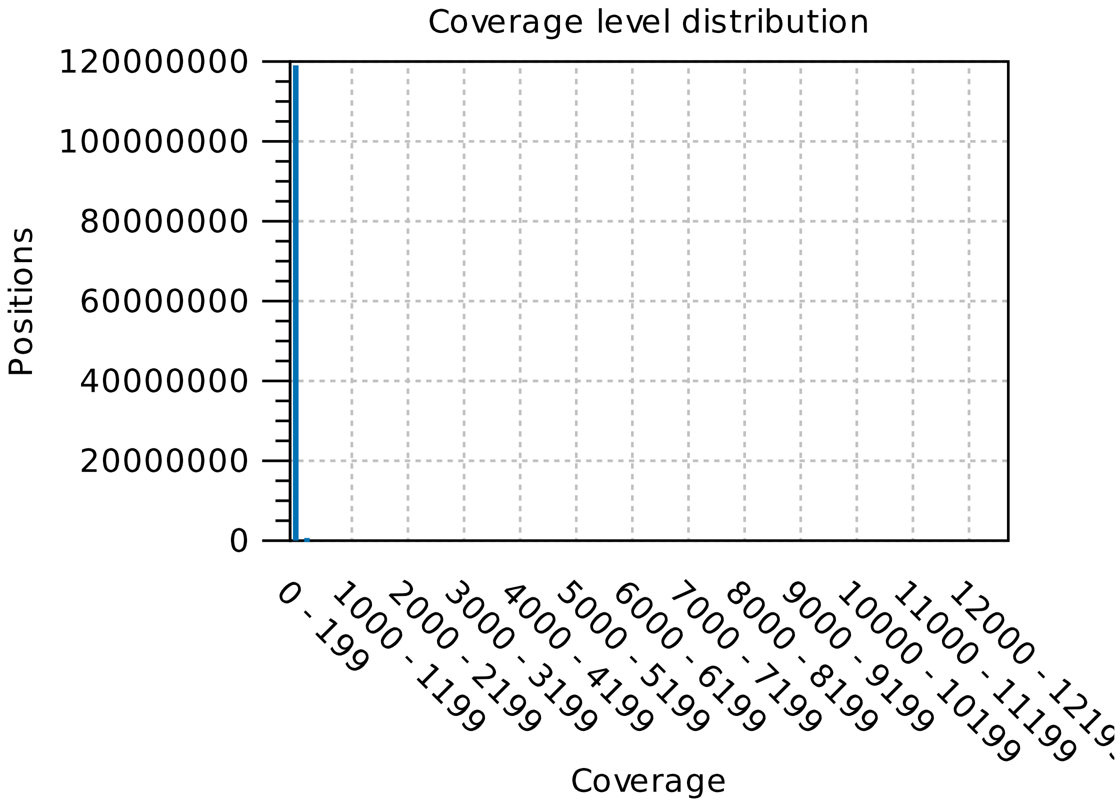

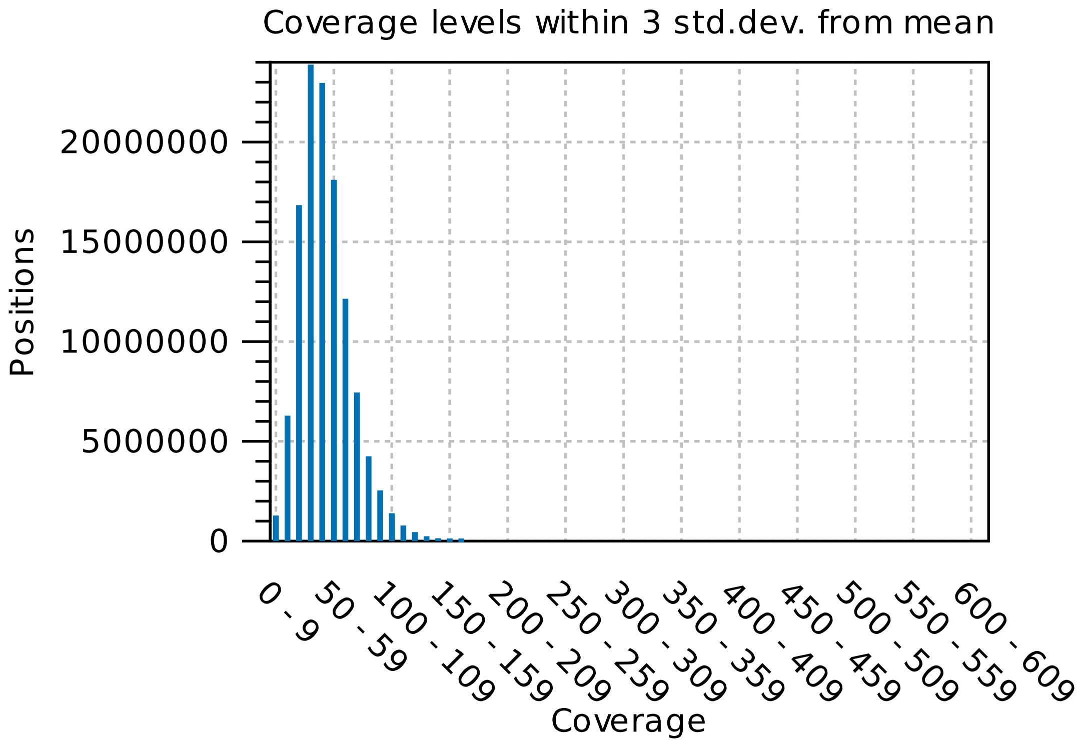


```
119,452,501 positions have coverage between 0 and 611.
215,249 positions have coverage above 611 (not shown in graph).
```

2.4 Coverage level distribution (excl. zero coverage regions)

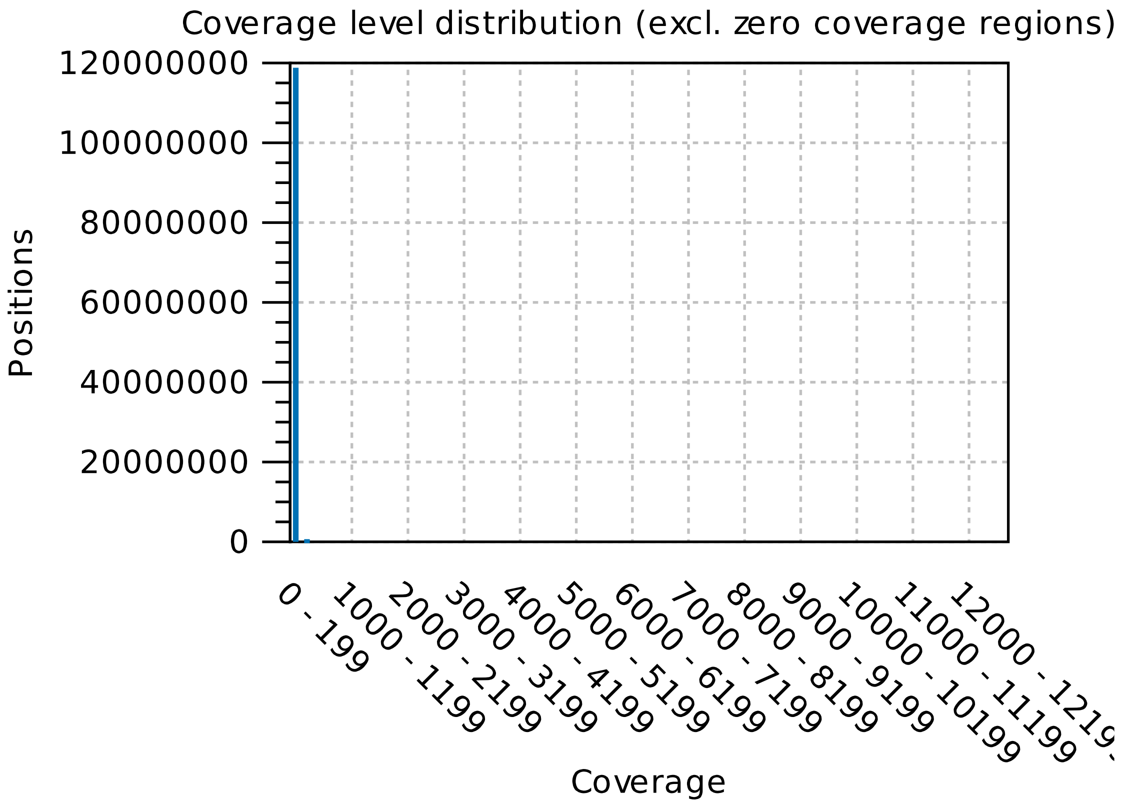

239,005 positions have zero coverage (not shown in graph).

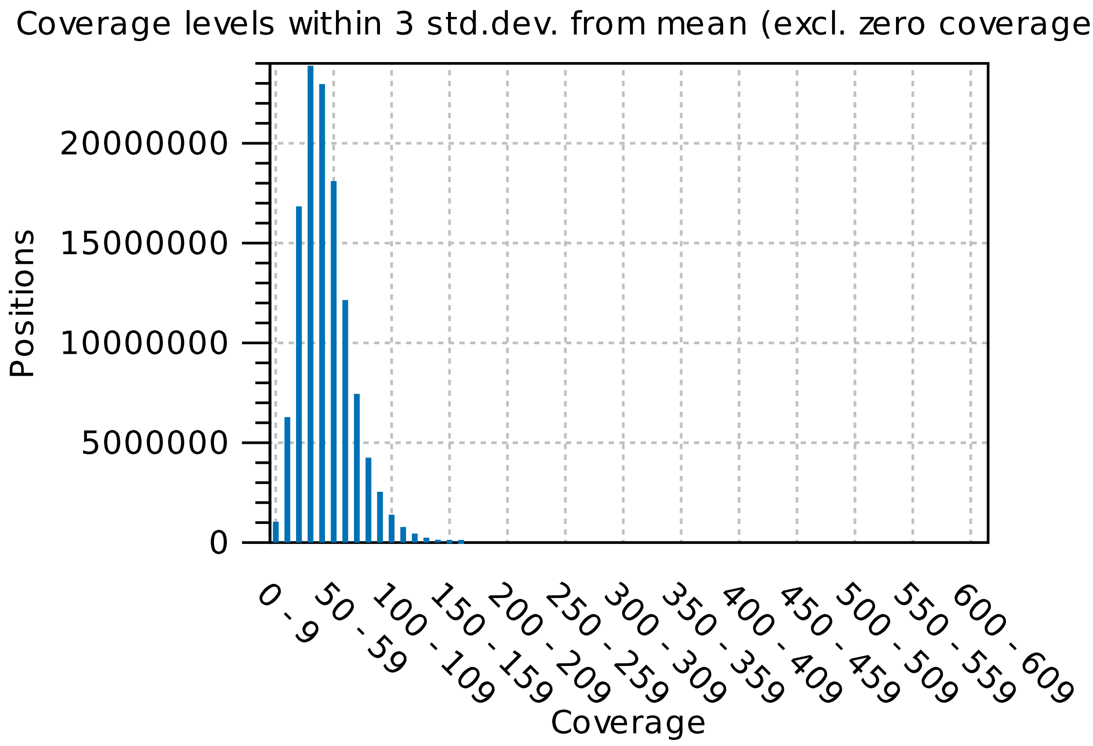

239,005 positions have coverage below 1 (not shown in graph).
119,213,536 positions have coverage between 1 and 612.

215,209 positions have coverage above 612 (not shown in graph).

2.5 Zero coverage regions

Count	350
Minimum length	1
Maximum length	54,395
Mean length	682.87
Standard deviation	4,597.82
Total length	239,005

2.6 Coverage vs GC Content Plot

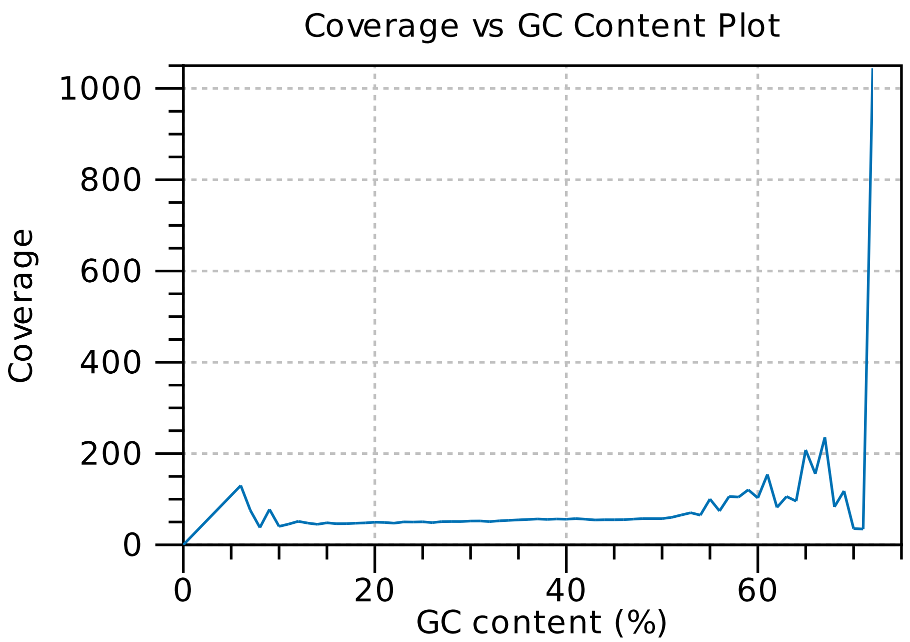

The plot displays, for each GC content level (0-100%), the mean read coverage of 100bp ref erence segments with that GC content.

3. Mapped reads

3.1 All mapped reads

Read count	1,625,293
Mean read length	4,689.63
Total read length	7,622,015,789

3.2 Non-specific matches

Non-specific matches

Read count	57,972
% of all mapped reads	3.57
Mean read length	5,009.90
Total read length	290,433,796

Distribution of match specificity (all matches)

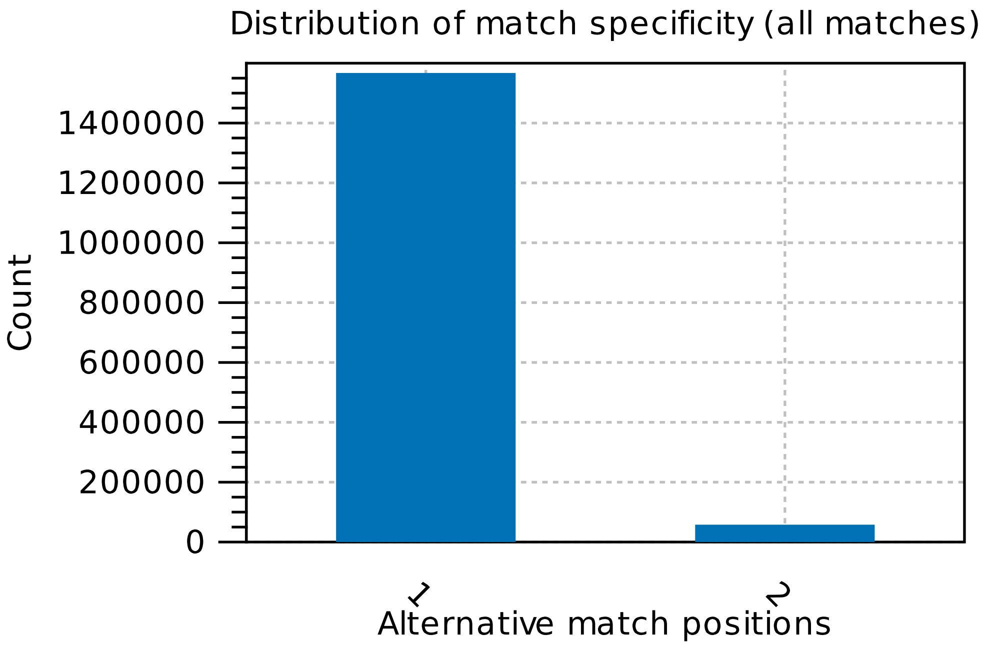

Distribution of match specificity (non-specific matches)

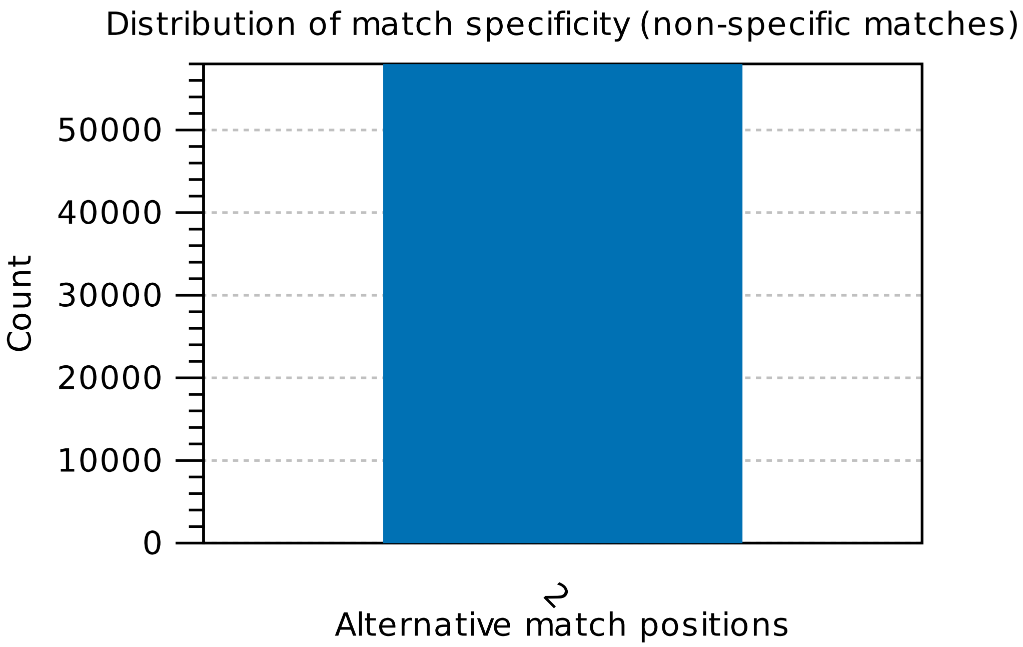

3.3 Non-perfect matches

Non-perfect matches

Read count	1,625,292
% of all mapped reads	100.00
Mean read length	4,689.63
Total read length	7,622,015,728

Distribution of mismatches (all matches)

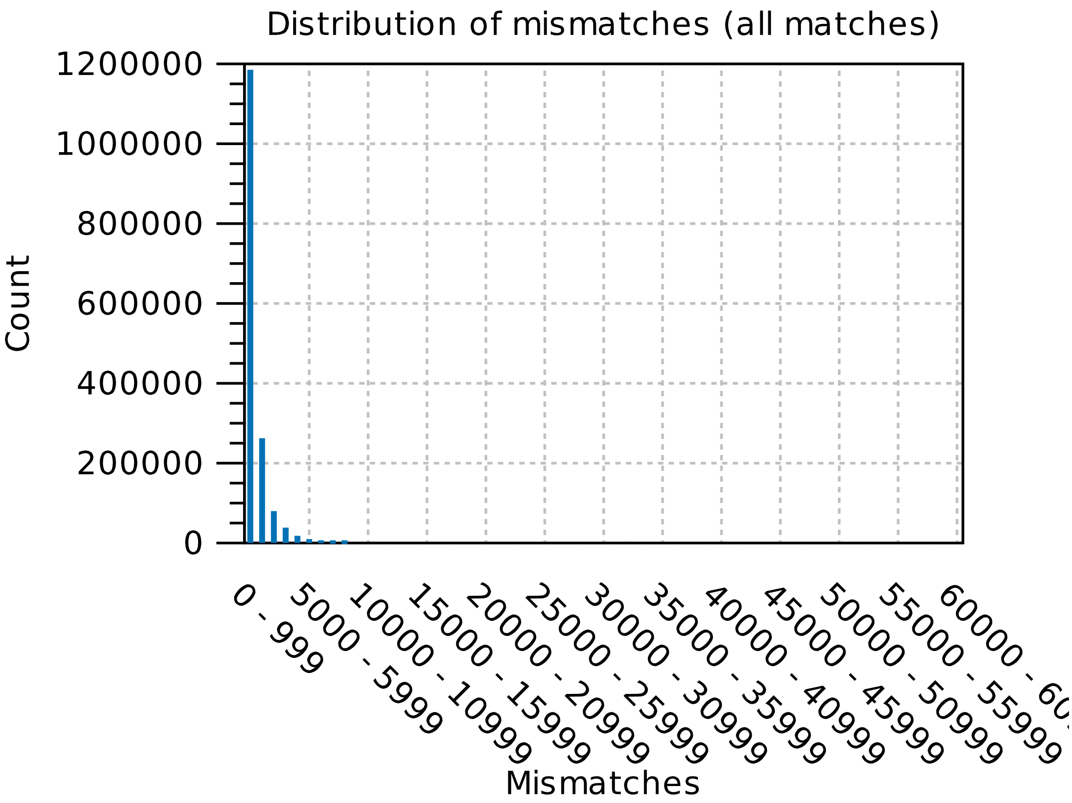

Distribution of mismatches (non-perfect matches)

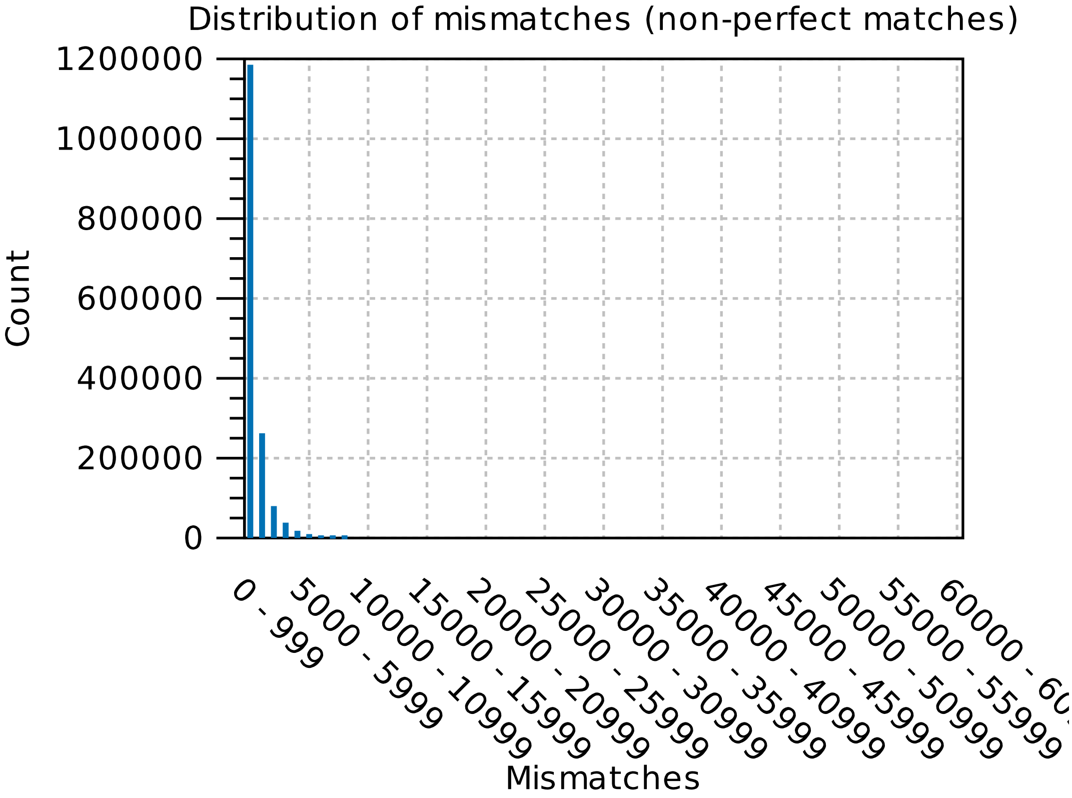

3.4 Read length distribution

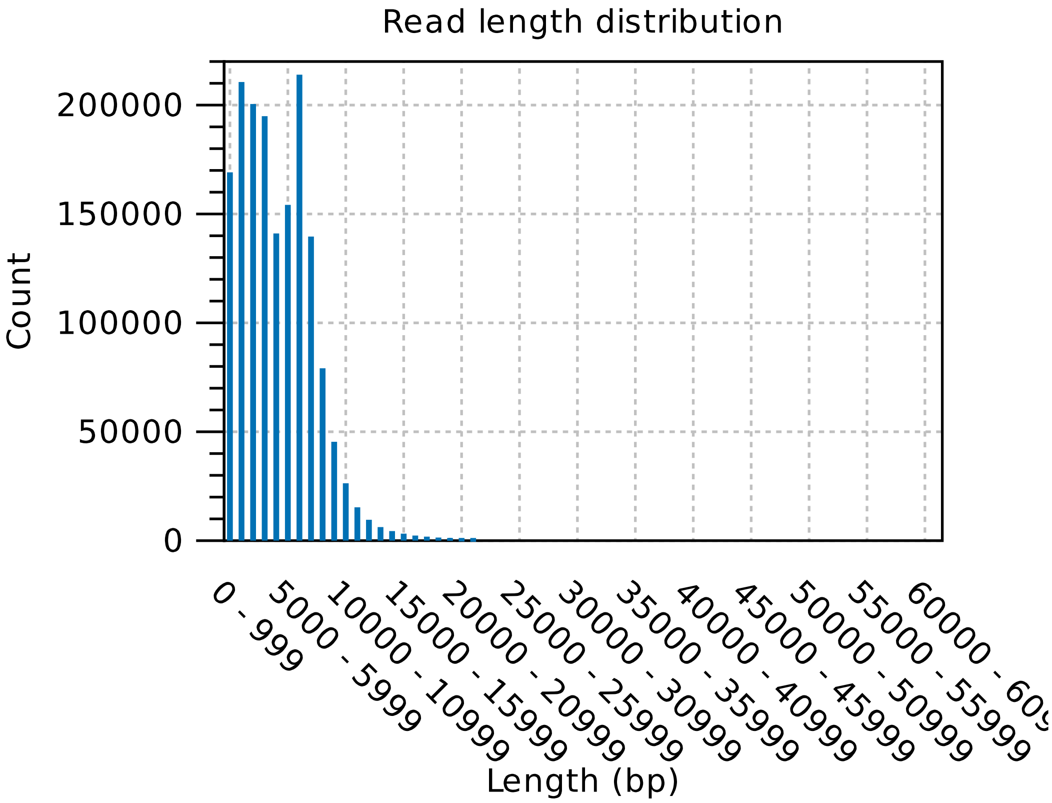

3.5 Insertion length distribution

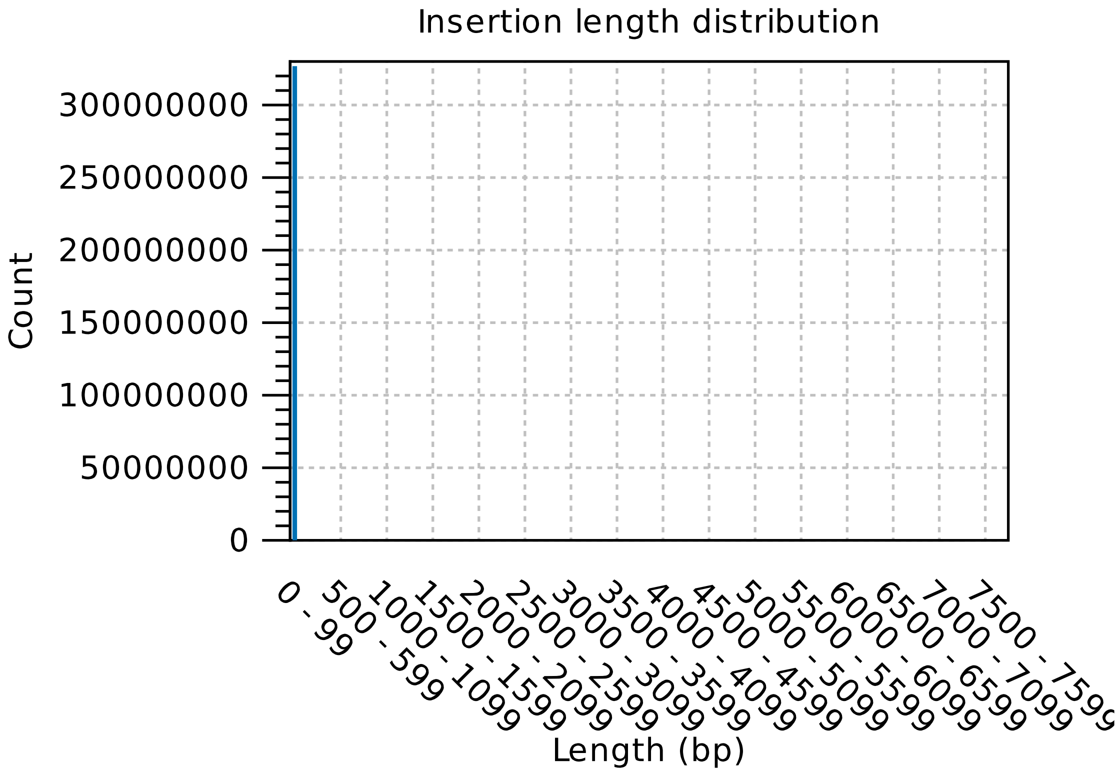

3.6 Deletion length distribution

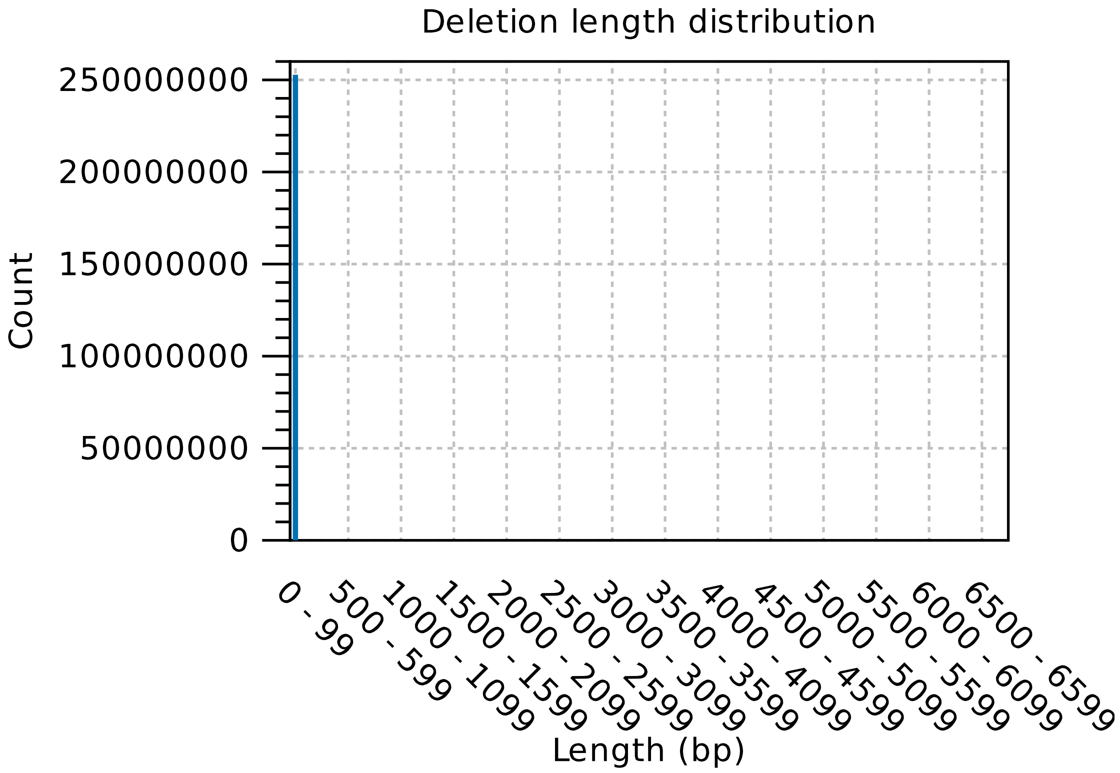

3.7 Nucleotide differences in reads relative to reference

Nucleotide in reference	% read bases that differ
A	5.14
C	6.25
G	6.34
T	5.14
-	1.43
Total	2.16

3.8 Nucleotide Mapping

Counts

Reference: A	1,975,712,470	6,948,852	4,738,593	6,783,385
Reference: C	7,177,666	1,133,537,666	3,086,381	4,850,486
Reference: G	4,890,629	3,116,773	1,135,544,899	7,394,222
Reference: T	6,816,517	4,701,095	7,031,103	1,976,436,386
Reference: -	130,202,251	86,369,132	86,253,001	130,415,861

Total	2,124,799,533	1,234,673,518	1,236,653,977	2,125,880,340

Read: -	Total
88,675,611	2,082,858,911
60,484,751	1,209,136,950
61,517,107	1,212,463,630
88,649,421	2,083,634,522
29,951,817,724	30,385,057,969
30,251,144,614	36,973,151,982

Percentages

Reference: A	5.34	0.02	0.01	0.02
Reference: C	0.02	3.07	0.01	0.01
Reference: G	0.01	0.01	3.07	0.02
Reference: T	0.02	0.01	0.02	5.35
Reference: -	0.35	0.23	0.23	0.35
Total	5.75	3.34	3.34	5.75

Read: -	Total
0.24	5.63
0.16	3.27
0.17	3.28
0.24	5.64
81.01	82.18
81.82	100.00

Relative errors (reference to read)

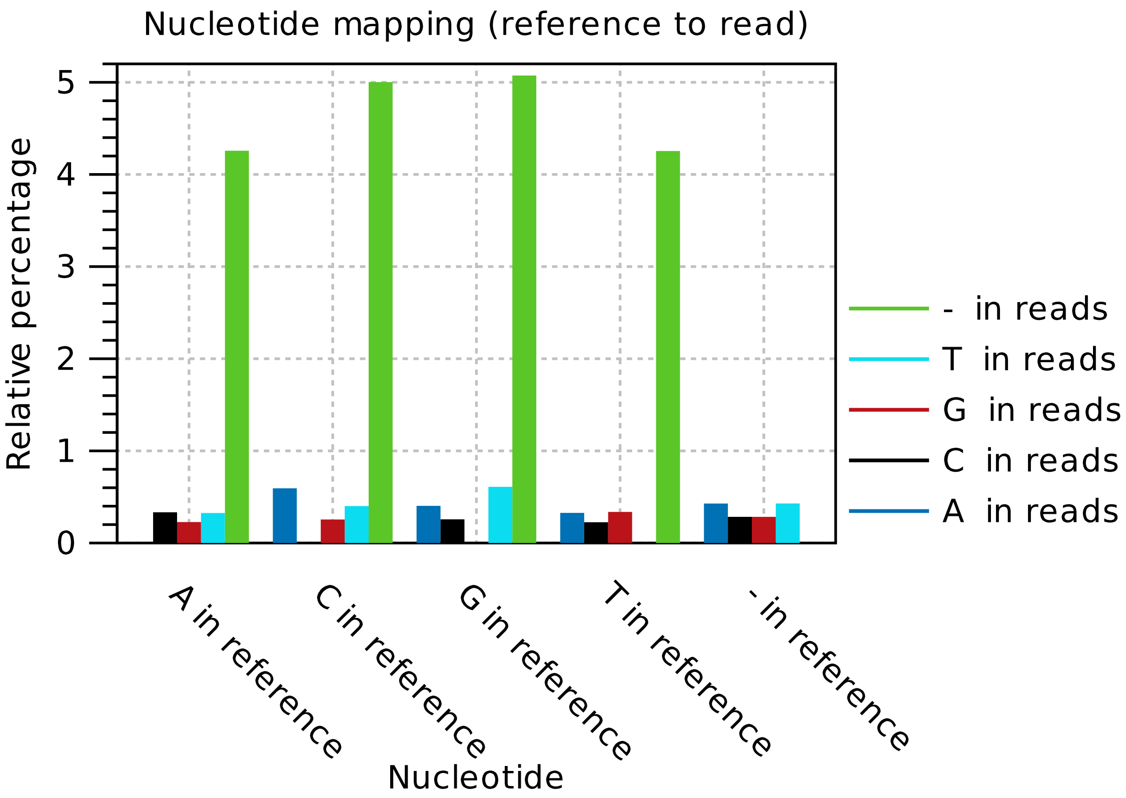

Bars depict off-diagonal element counts divided by counts in the column with header "total ".

Error counts (reference to read)

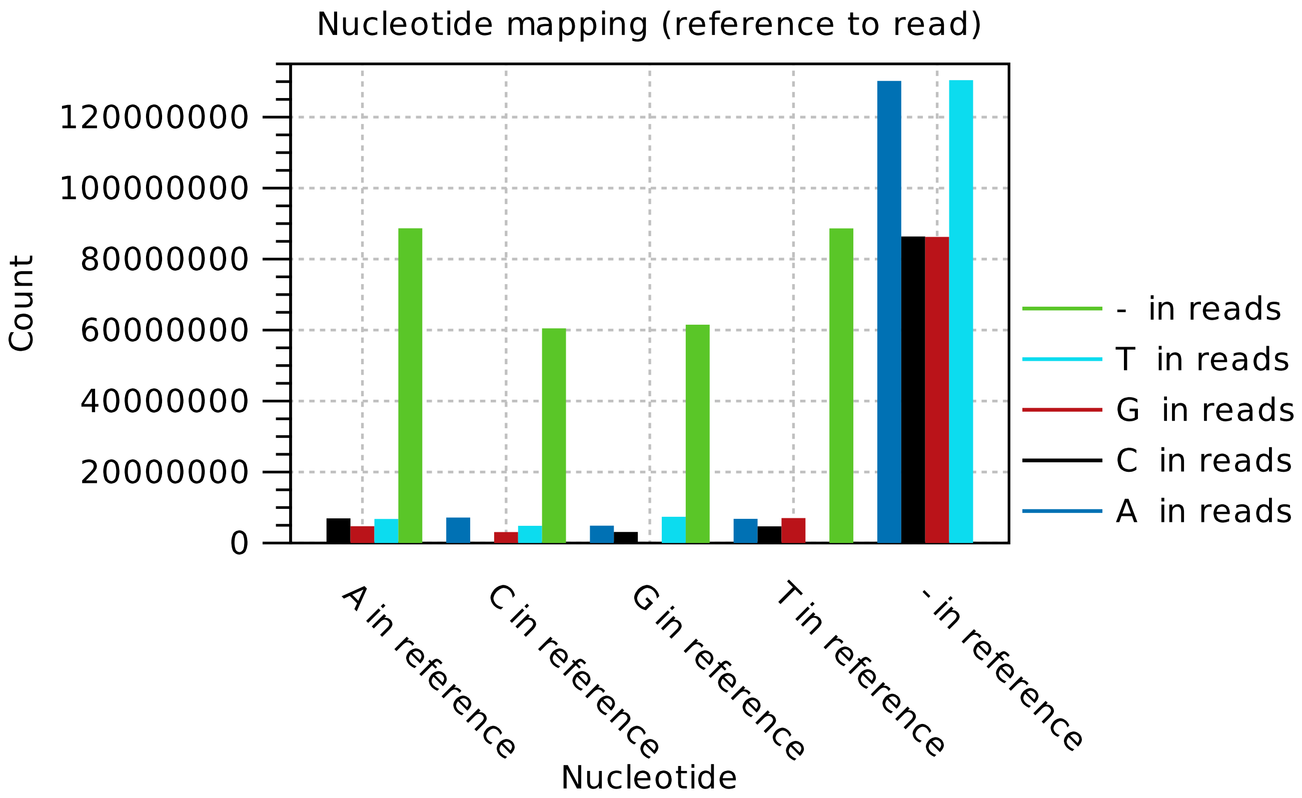

Bars depict off-diagonal element counts.

Relative errors (read to reference)

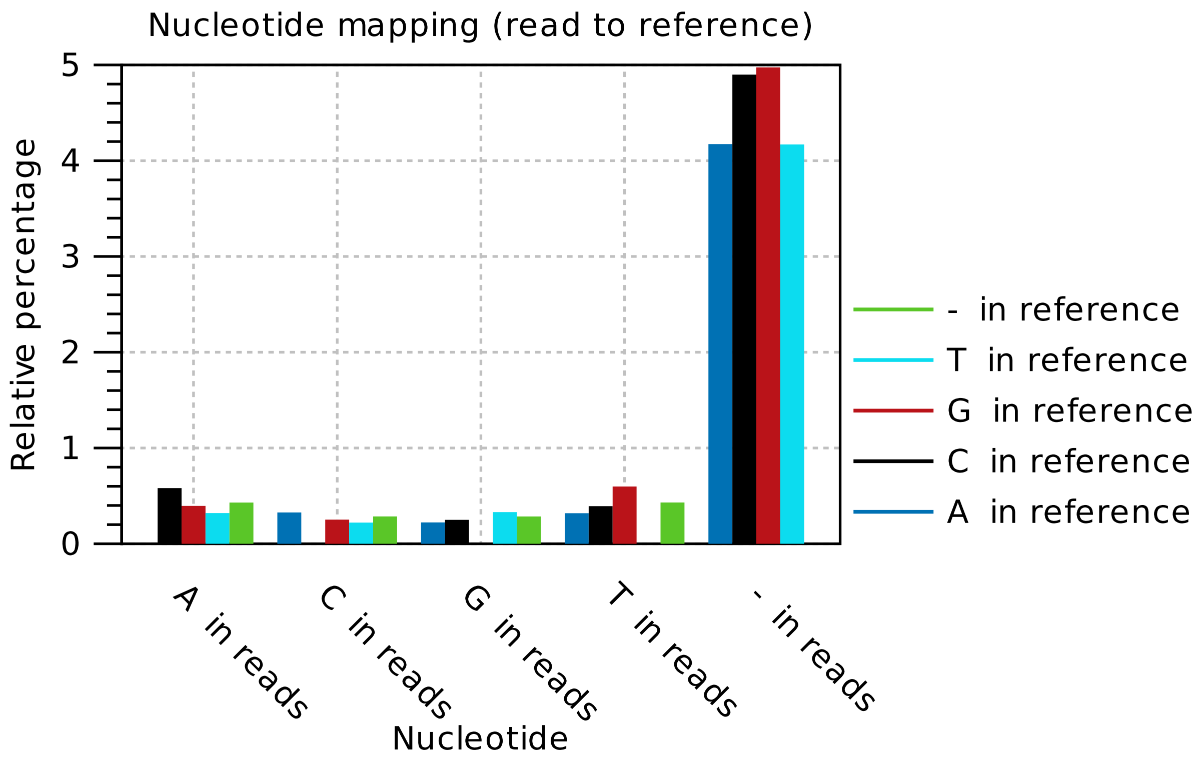

Bars depict off-diagonal element counts divided by counts in the row with header "total".

Error counts (read to reference)

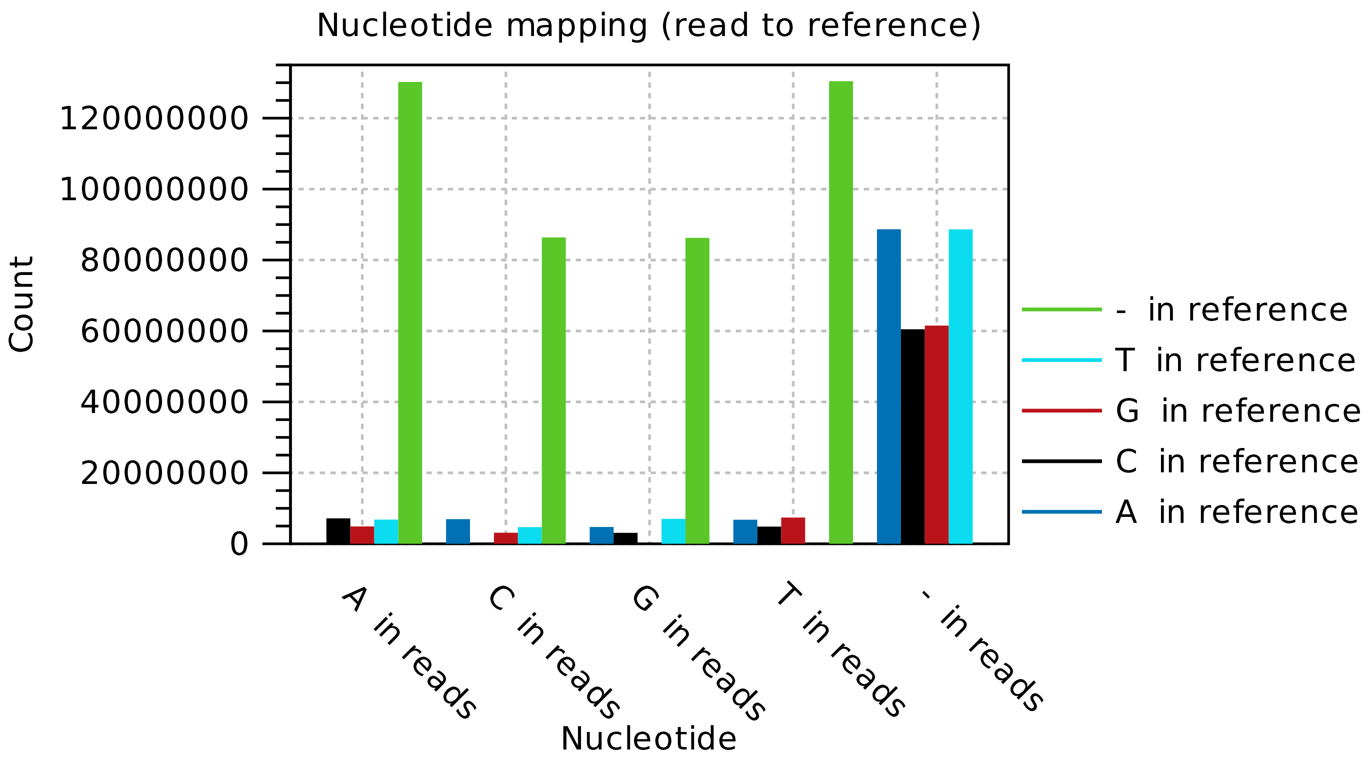

Bars depict off-diagonal element counts.

3.9 Quality for match distribution

There are no qualities.

3.10 Quality for mismatch distribution

There are no qualities.

3.11 Mismatch fraction per read position

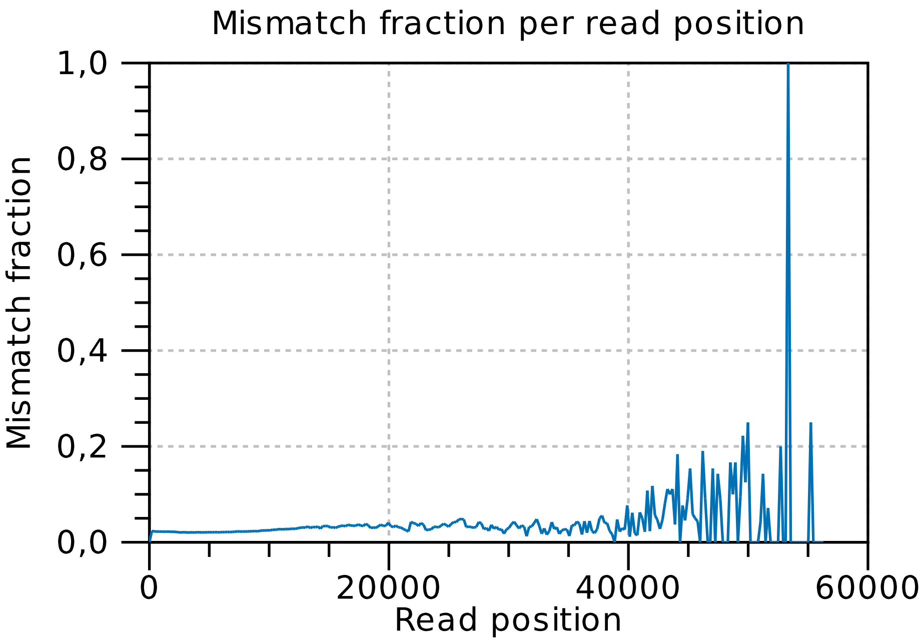

The plot displays the mismatch fraction per read position in the sequenced segments

3.12 Unaligned ends

Read count	2,254,744
% of all mapped reads	138.73
Positions covered	1,965,677
Positions covered in % of reference	1.64
Positions covered in % of bases covered	1.65

Reads with one unaligned end

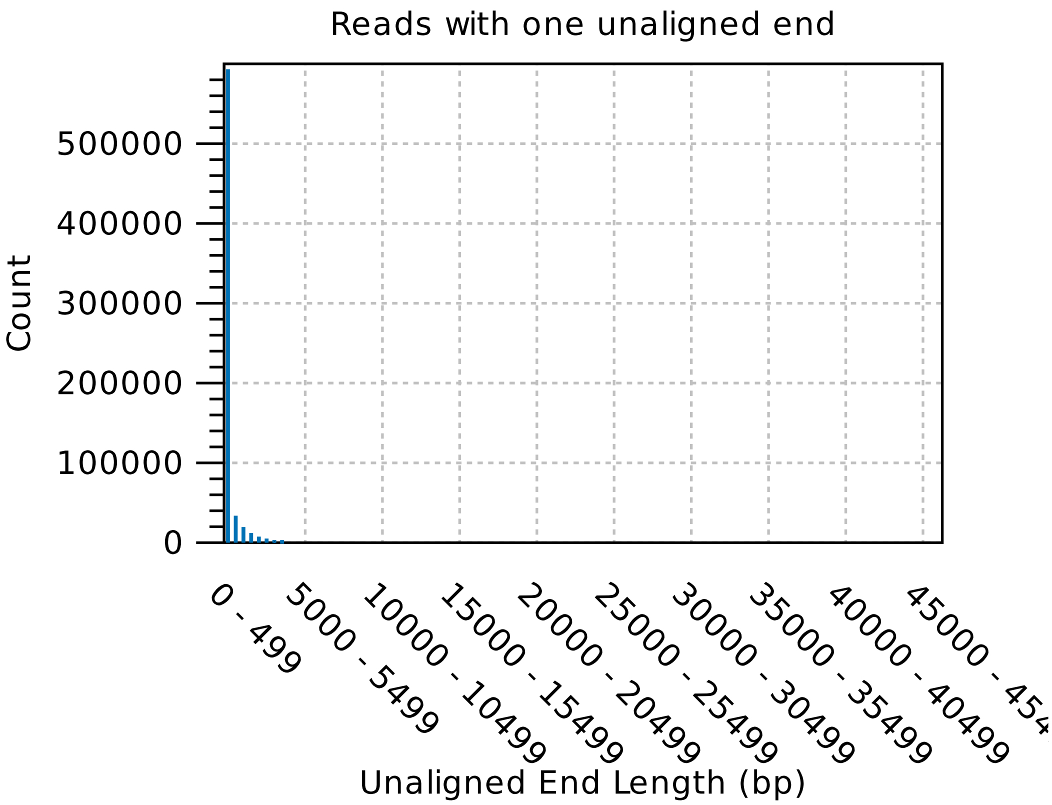

Reads with two unaligned ends

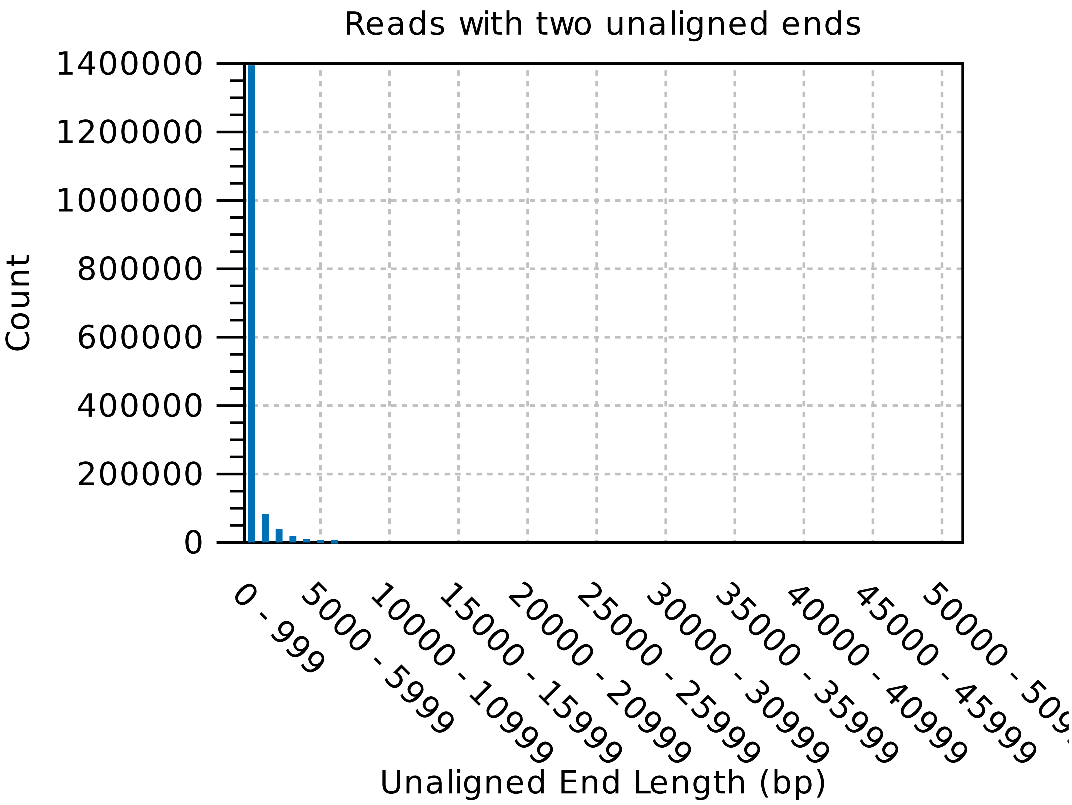

Supplement: Supplementary file 3 — Mapping Report for Ath-Clean BLASR-mapping. (PDF 418 kb) [file 12864_2018_5164_MOESM3_ESM.pdf]
