# Supplementary material for: Correcting palindromes in long reads after whole-genome amplification
Source: BMC Genomics. 2018 Nov 6;19:798. doi: 10.1186/s12864-018-5164-1 (PMC6218980; doi:10.1186/s12864-018-5164-1)

Mapping Report for ath.all.raw BLASR-mapping
Type: Read mapping
Reference count: 7
Generated by: warri004
Date: Sun May 08 19:11:45 CEST 2016
Software: CLC Genomics Server 7.5.1

Table of contents

1. Summary 3
2. References 3
    2.1 Reference coverage 3
    2.2 Coverage statistics 3
    2.3 Coverage level distribution 4
    2.4 Coverage level distribution (excl. zero coverage regions) 5
    2.5 Zero coverage regions 6
    2.6 Coverage vs GC Content Plot 6
3. Mapped reads 6
    3.1 All mapped reads 6
    3.2 Non-specific matches 7
    3.3 Non-perfect matches 8
    3.4 Read length distribution 11
    3.5 Insertion length distribution 12
    3.6 Deletion length distribution 13
    3.7 Nucleotide differences in reads relative to reference 13
    3.8 Nucleotide Mapping 13
    3.9 Quality for match distribution 17
    3.10 Quality for mismatch distribution 17
    3.11 Mismatch fraction per read position 18
    3.12 Unaligned ends 18

1. Summary

Reference count	7
Type	Read mapping
Total reference length	119,667,750
GC contents in %	36.00
Total read count	897,995
Mean read length	8,986.75
Total read length	8,070,059,681

2. References

2.1 Reference coverage

Total reference length	119,667,750
% GC	36.00
Total consensus length	-
Fraction of reference covered	1.00

2.2 Coverage statistics

Total reference length	119,667,750
Minimum coverage	0
Maximum coverage	9,052
Average coverage	40.51
Standard deviation	134.33
Minimum excl. zero coverage regions	1
Average excl. zero coverage regions	40.60
Standard deviation excl. zero coverage regions	134.46

2.3 Coverage level distribution

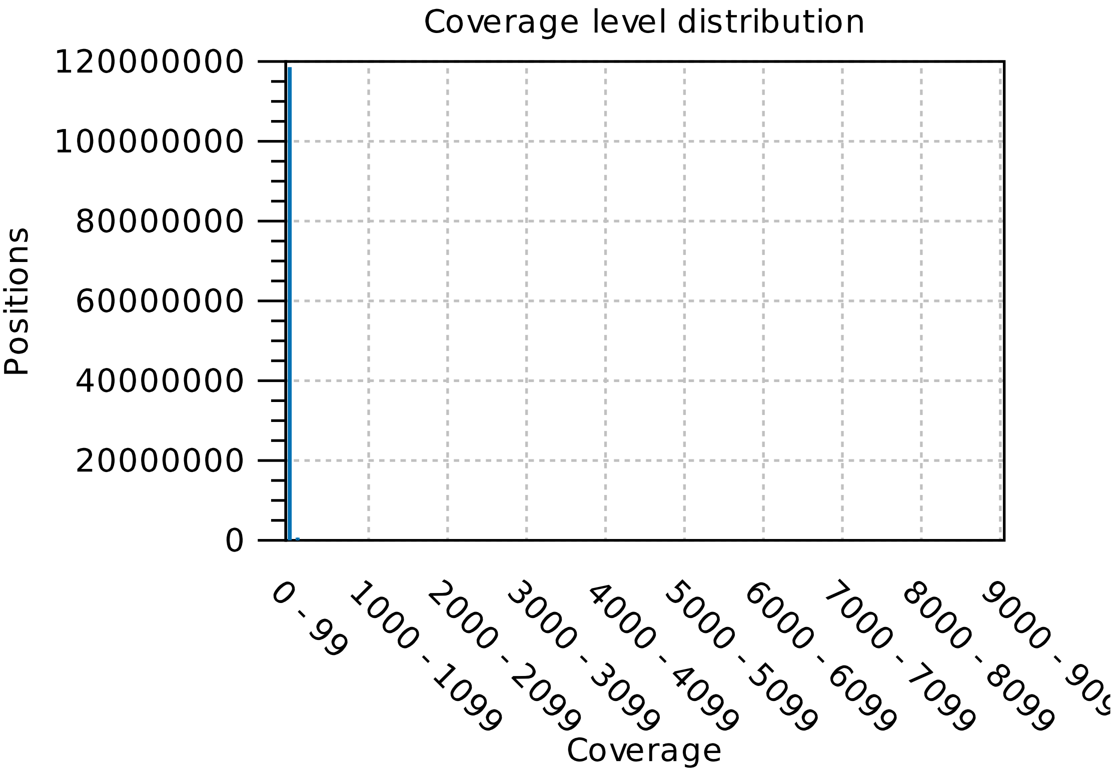

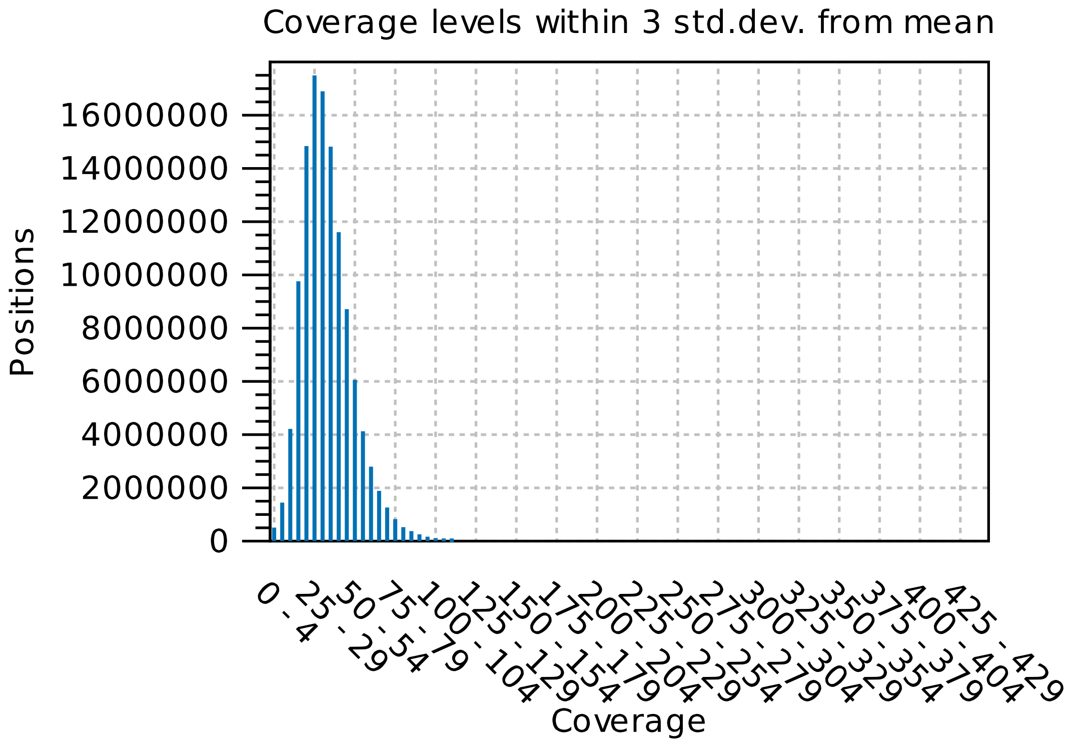


```
119,455,733 positions have coverage between 0 and 443.
212,017 positions have coverage above 443 (not shown in graph).
```

2.4 Coverage level distribution (excl. zero coverage regions)

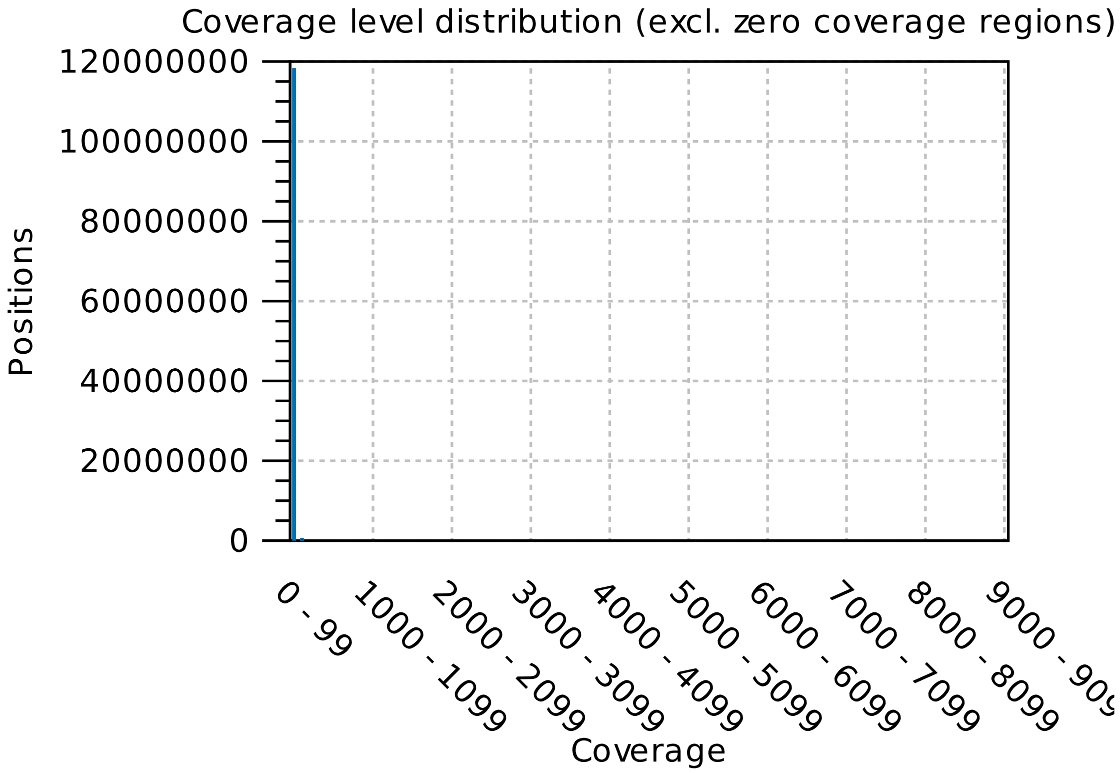

239,624 positions have zero coverage (not shown in graph).

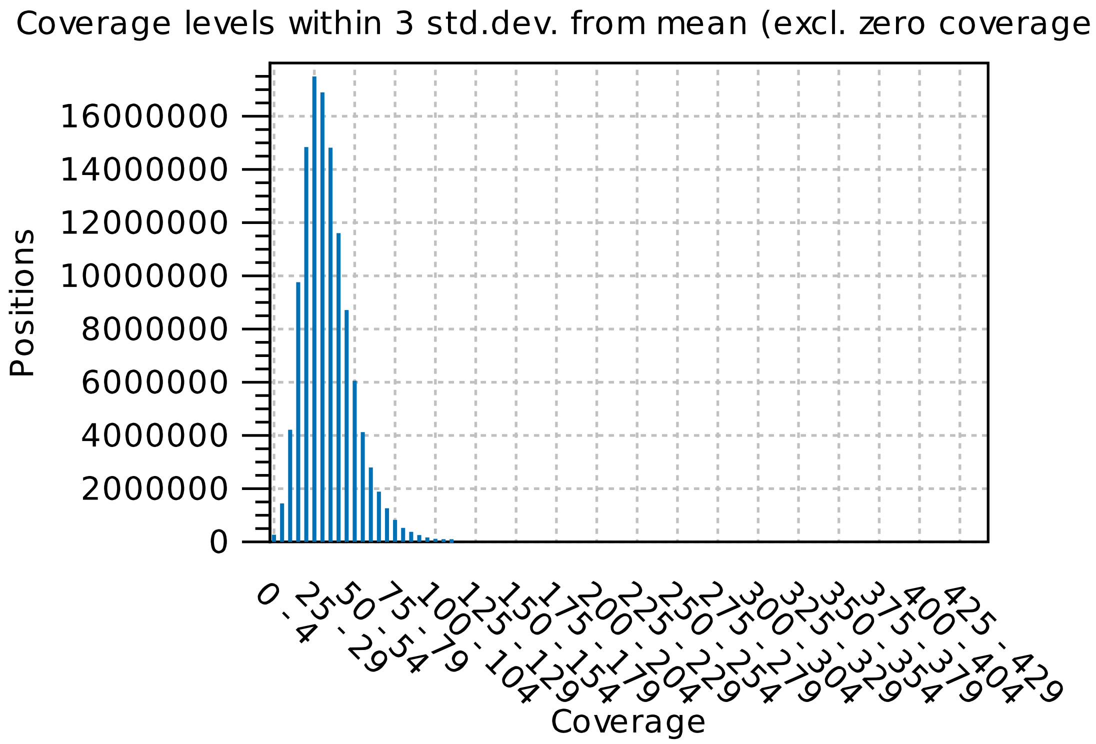

239,624 positions have coverage below 1 (not shown in graph).
119,216,109 positions have coverage between 1 and 443.
212,017 positions have coverage above 443 (not shown in graph).

2.5 Zero coverage regions

Count	342
Minimum length	1
Maximum length	55,751
Mean length	700.65
Standard deviation	4,882.76
Total length	239,624

2.6 Coverage vs GC Content Plot

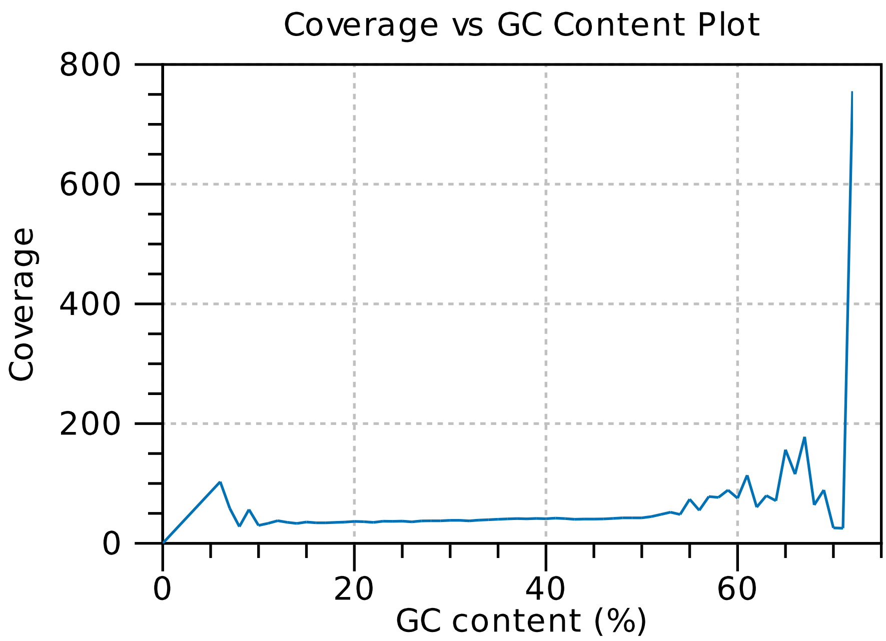

The plot displays, for each GC content level (0-100%), the mean read coverage of 100bp ref erence segments with that GC content.

3. Mapped reads

3.1 All mapped reads

Read count	897,995
Mean read length	8,986.75
Total read length	8,070,059,681

3.2 Non-specific matches

Non-specific matches

Read count	32,658
% of all mapped reads	3.64
Mean read length	8,841.40
Total read length	288,742,282

Distribution of match specificity (all matches)

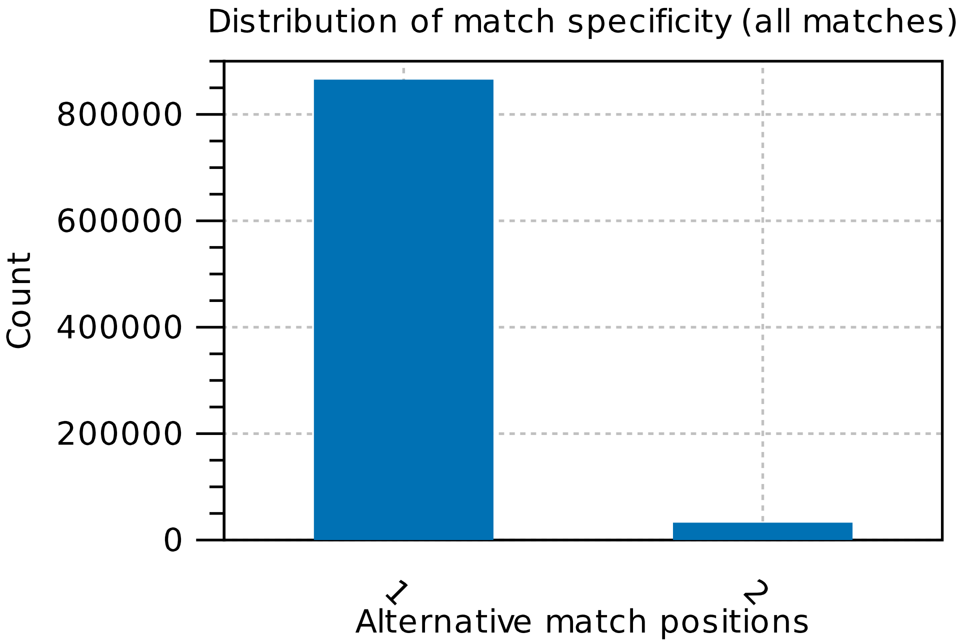

Distribution of match specificity (non-specific matches)

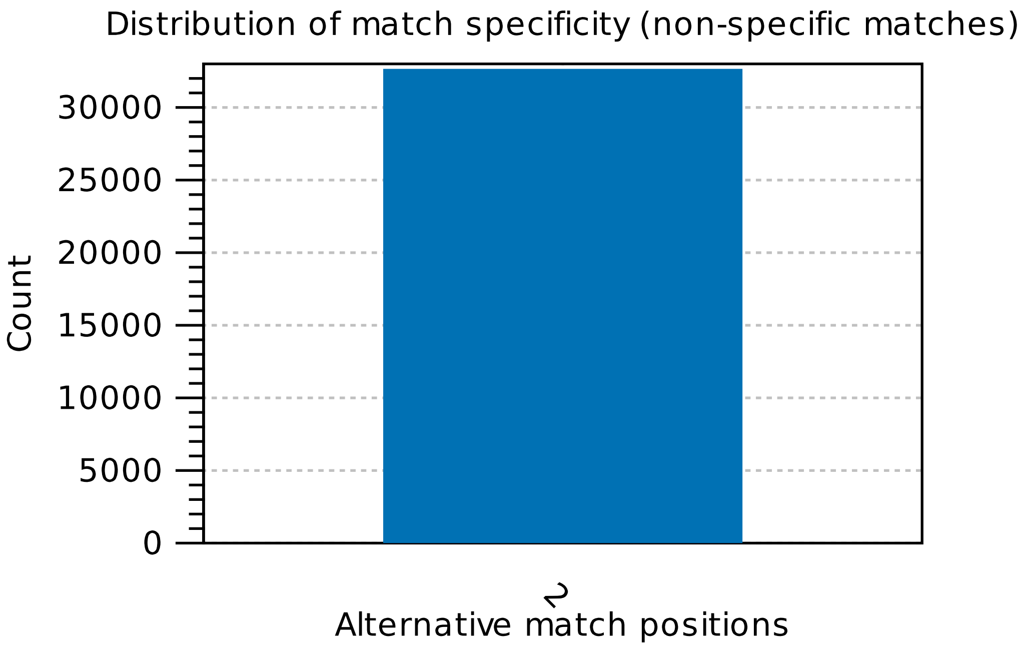

3.3 Non-perfect matches

Non-perfect matches

Read count	897,995
% of all mapped reads	100.00
Mean read length	8,986.75
Total read length	8,070,059,681

Distribution of mismatches (all matches)

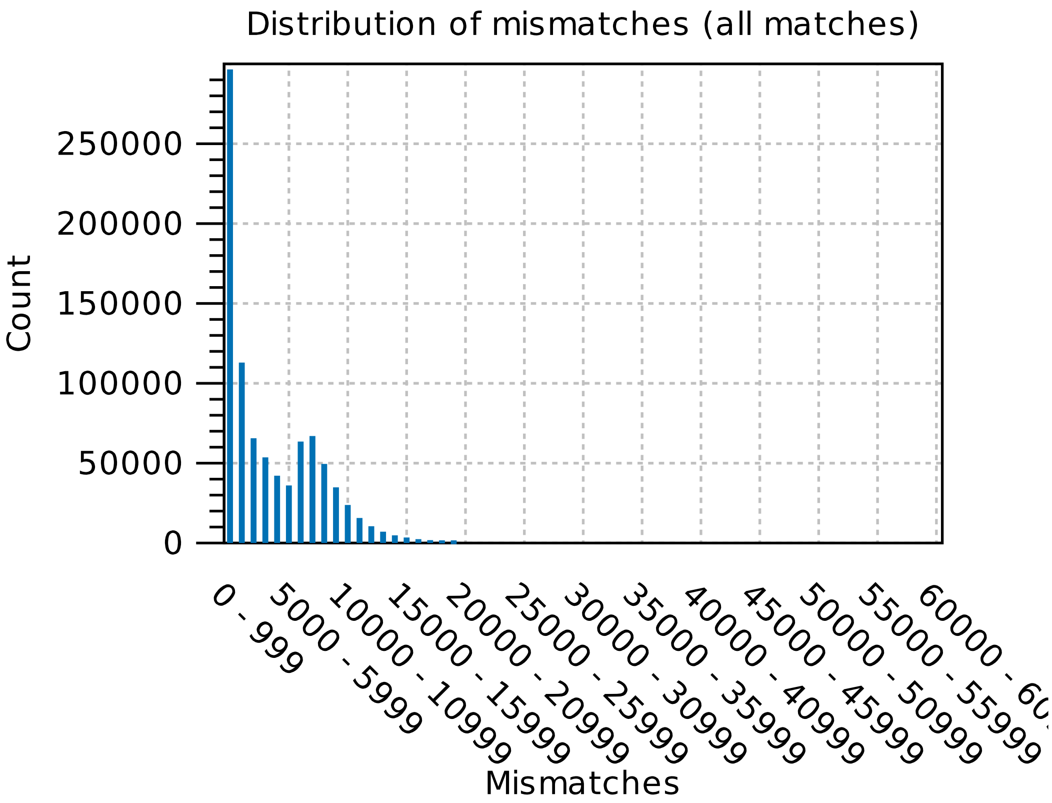

Distribution of mismatches (non-perfect matches)

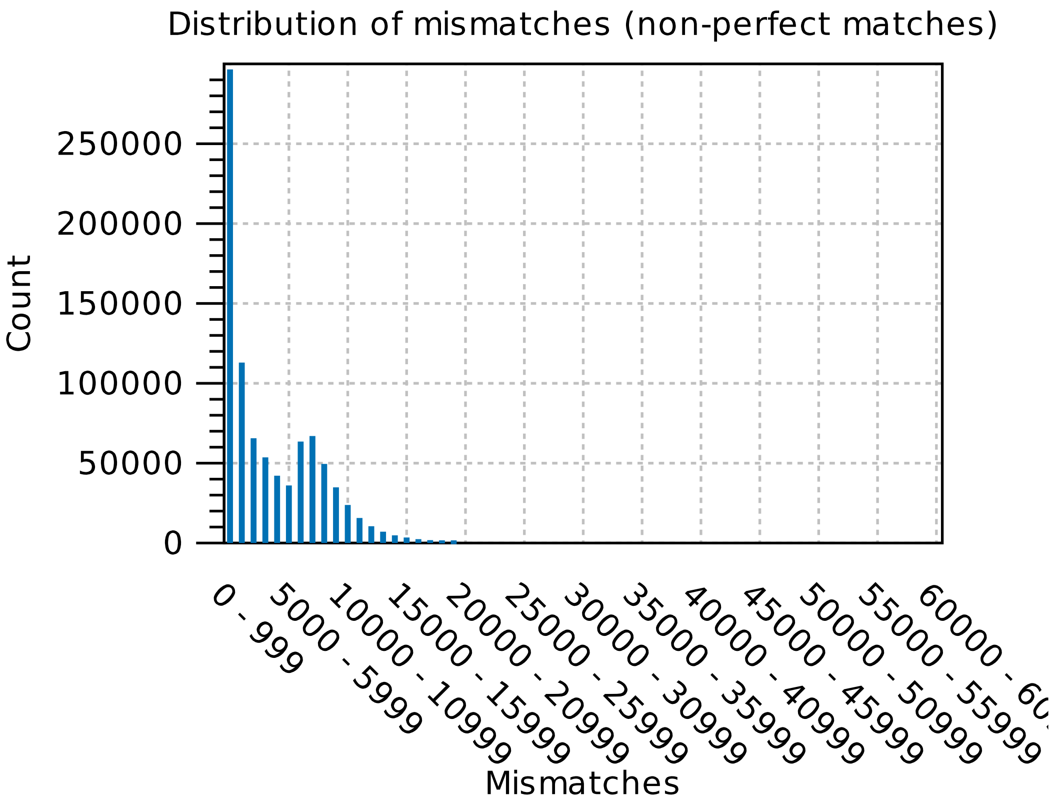

3.4 Read length distribution

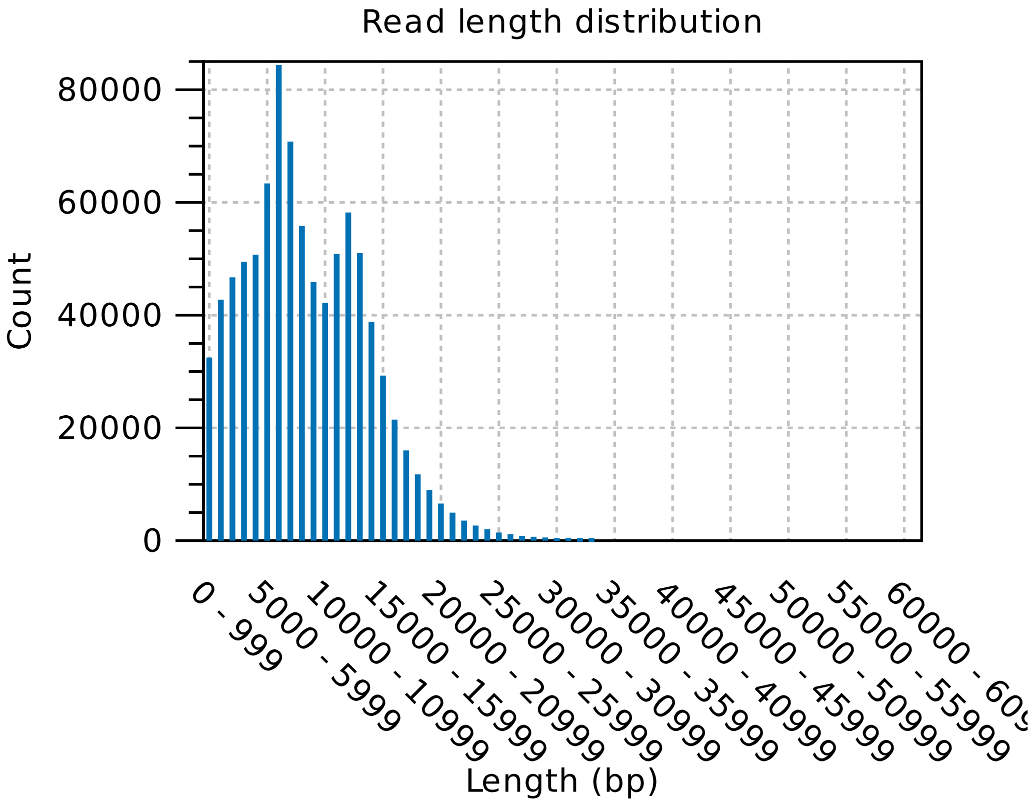

3.5 Insertion length distribution

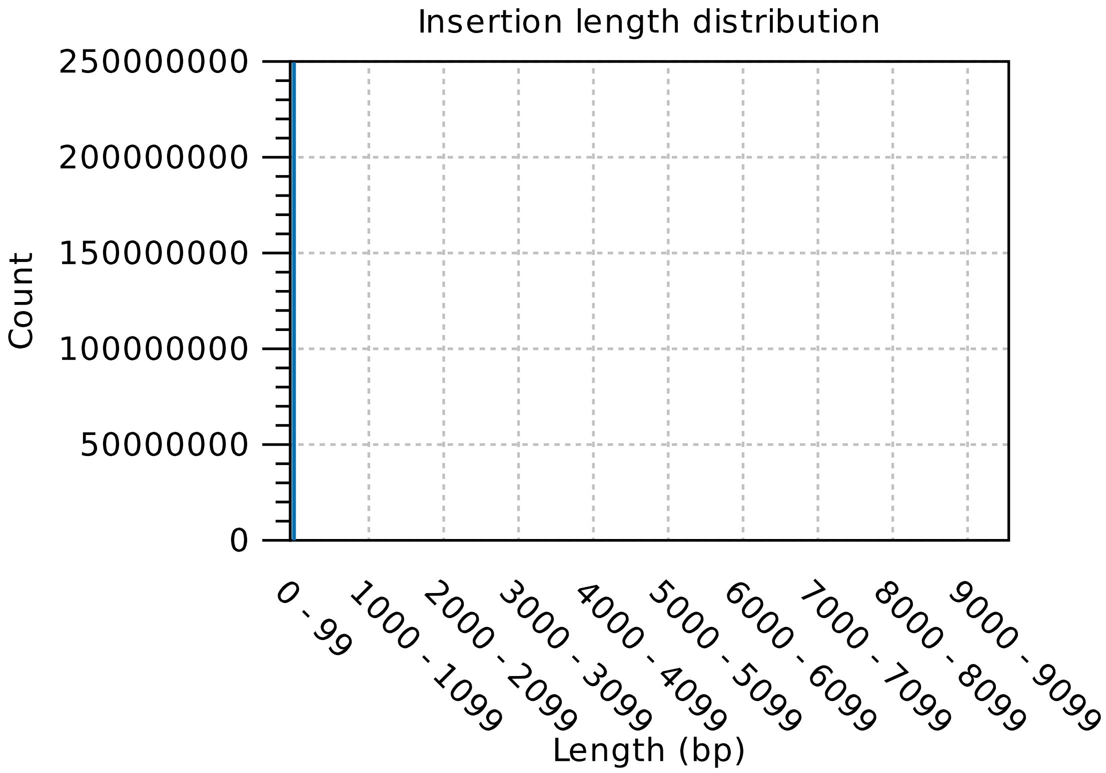

3.6 Deletion length distribution

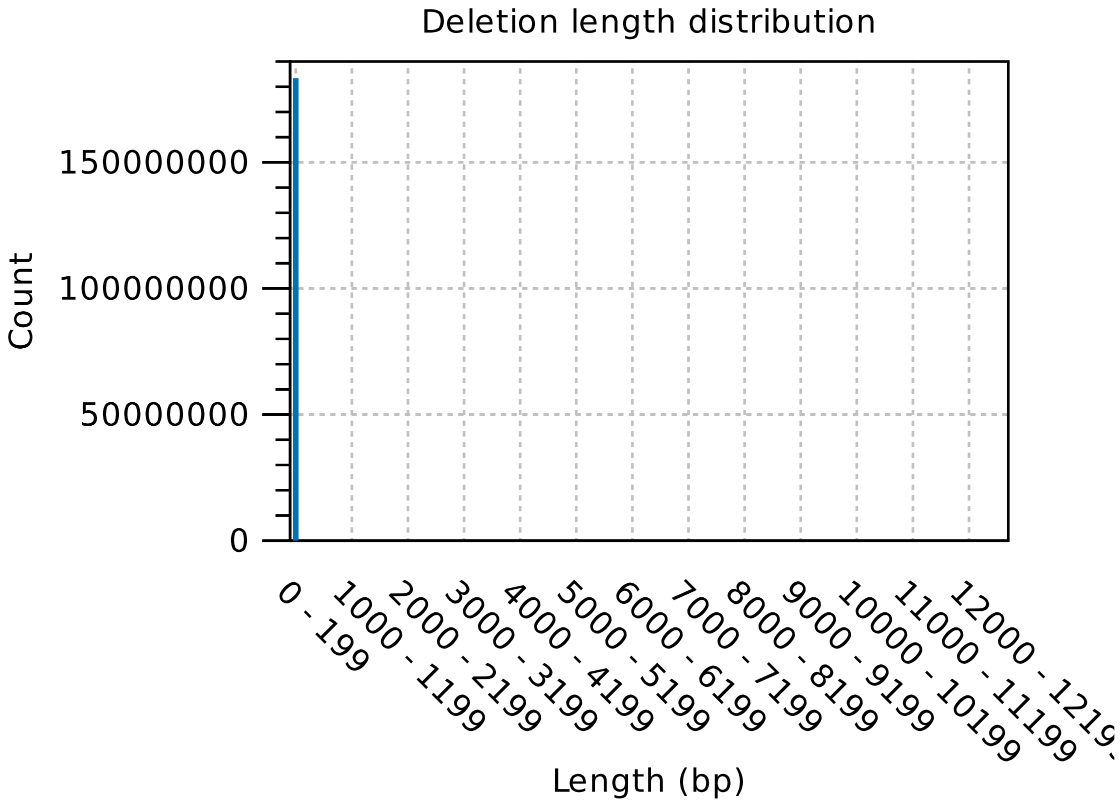

3.7 Nucleotide differences in reads relative to reference

Nucleotide in reference	% read bases that differ
A	5.21
C	6.32
G	6.41
T	5.19
-	1.50
Total	2.21

3.8 Nucleotide Mapping

Counts

Reference: A	1,453,303,550	5,194,493	3,610,864	5,052,653
Reference: C	5,321,675	833,180,529	2,338,108	3,591,947
Reference: G	3,640,546	2,366,955	835,944,332	5,489,773
Reference: T	5,056,101	3,550,412	5,234,665	1,452,956,153
Reference: -	103,461,645	69,868,580	69,771,025	103,615,842

Total	1,570,783,517	914,160,969	916,898,994	1,570,706,368

Read: -	Total
65,956,118	1,533,117,678
44,970,549	889,402,808
45,742,576	893,184,182
65,741,798	1,532,539,129
22,831,701,445	23,178,418,537
23,054,112,486	28,026,662,334

Percentages

Reference: A	5.19	0.02	0.01	0.02
Reference: C	0.02	2.97	0.01	0.01
Reference: G	0.01	0.01	2.98	0.02
Reference: T	0.02	0.01	0.02	5.18
Reference: -	0.37	0.25	0.25	0.37
Total	5.60	3.26	3.27	5.60

Read: -	Total
0.24	5.47
0.16	3.17
0.16	3.19
0.23	5.47
81.46	82.70
82.26	100.00

Relative errors (reference to read)

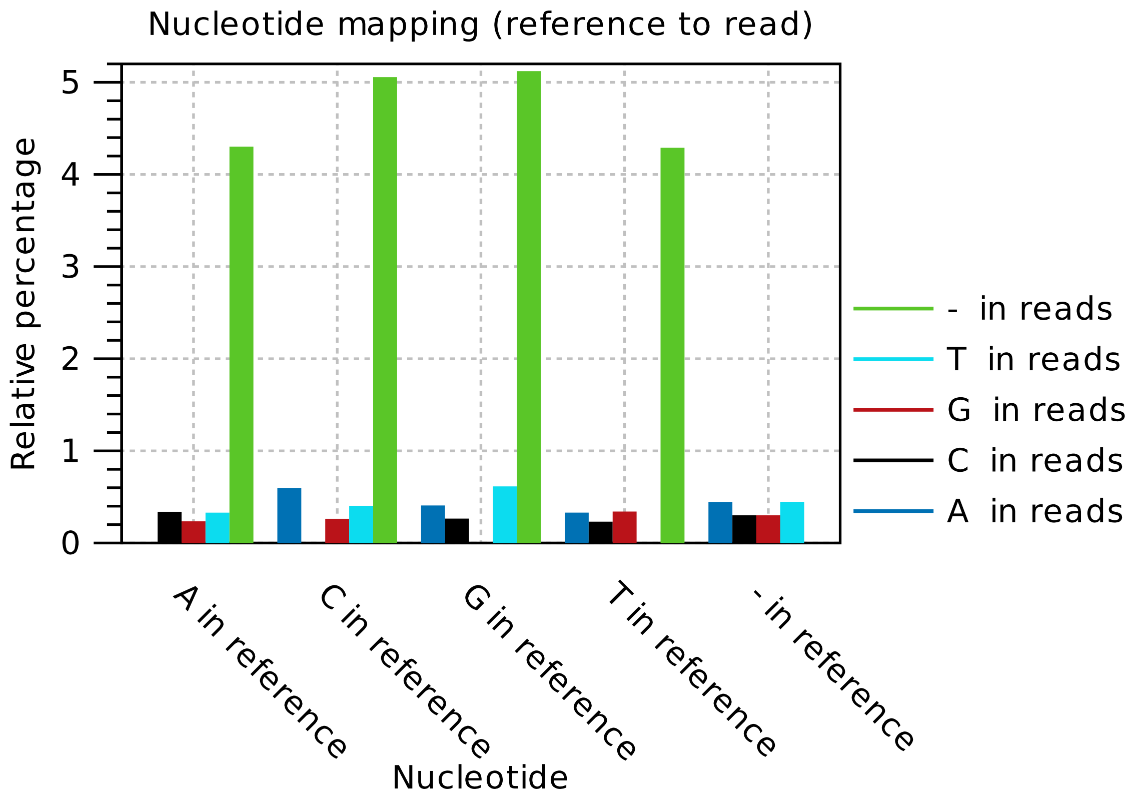

`Bars depict off-diagonal element counts divided by counts in the column with header "total ".`

Error counts (reference to read)

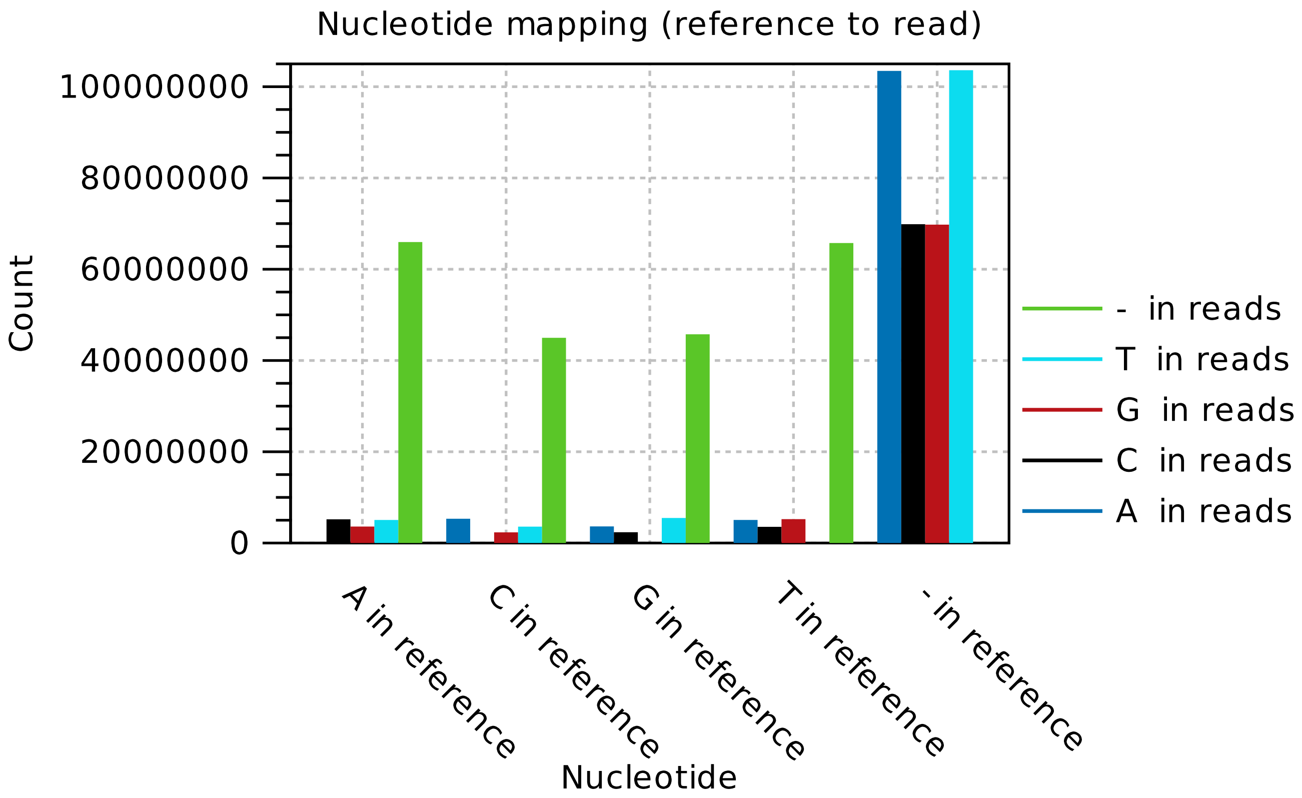


```
Bars depict off-diagonal element counts.
```

Relative errors (read to reference)

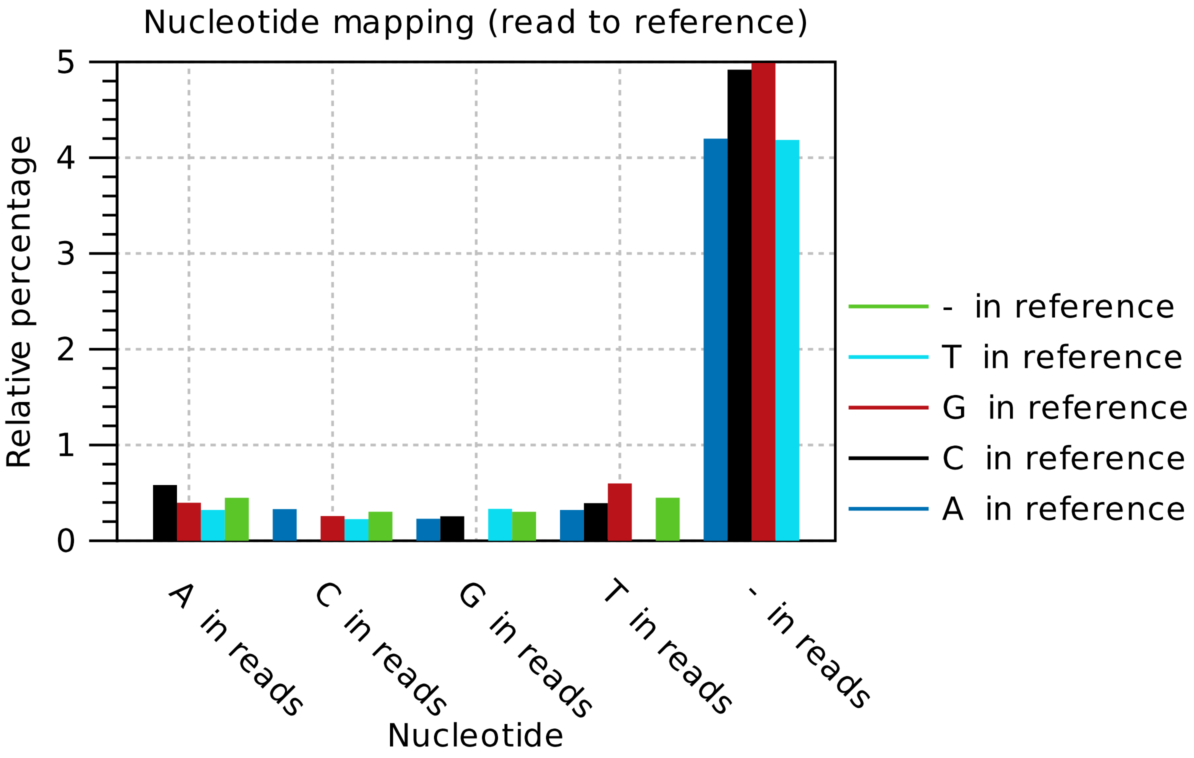

Bars depict off-diagonal element counts divided by counts in the row with header "total".

Error counts (read to reference)

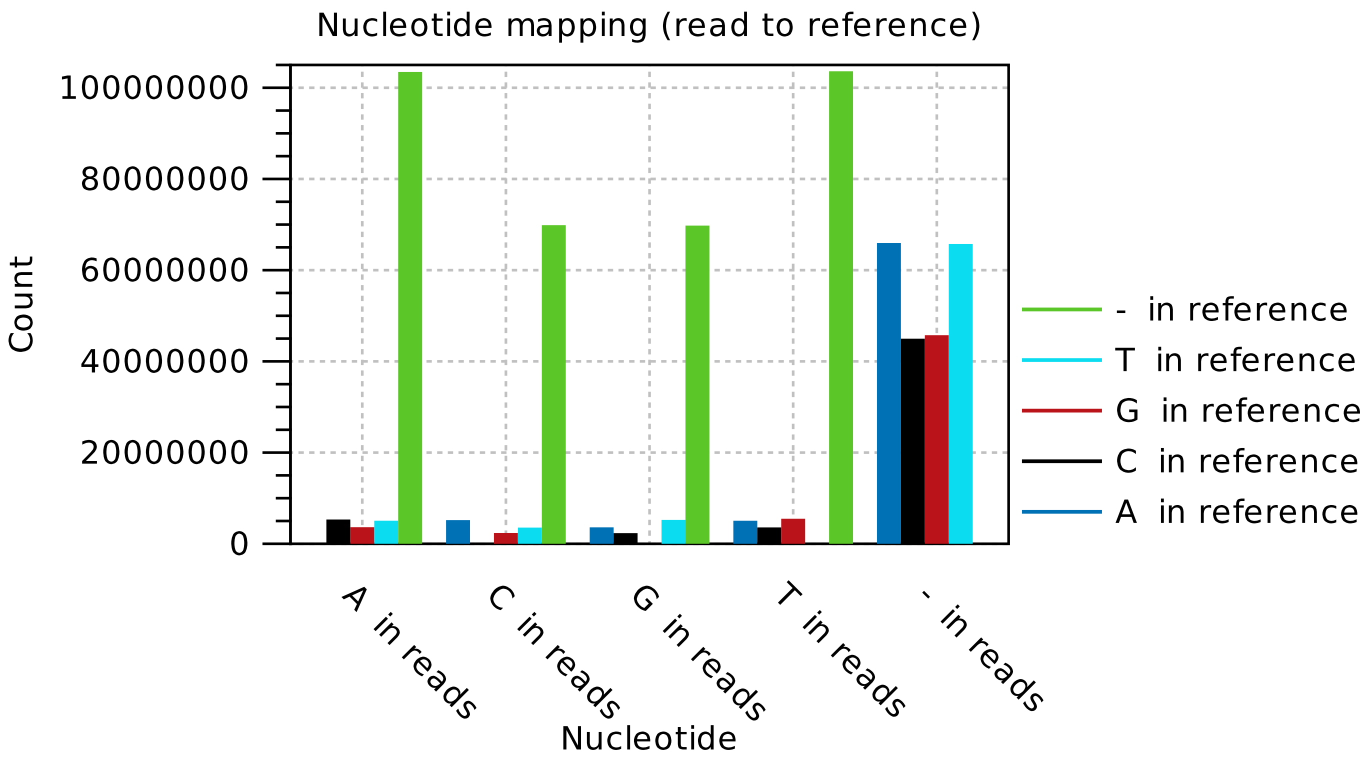

Bars depict off-diagonal element counts.

3.9 Quality for match distribution

There are no qualities.

3.10 Quality for mismatch distribution

There are no qualities.

3.11 Mismatch fraction per read position

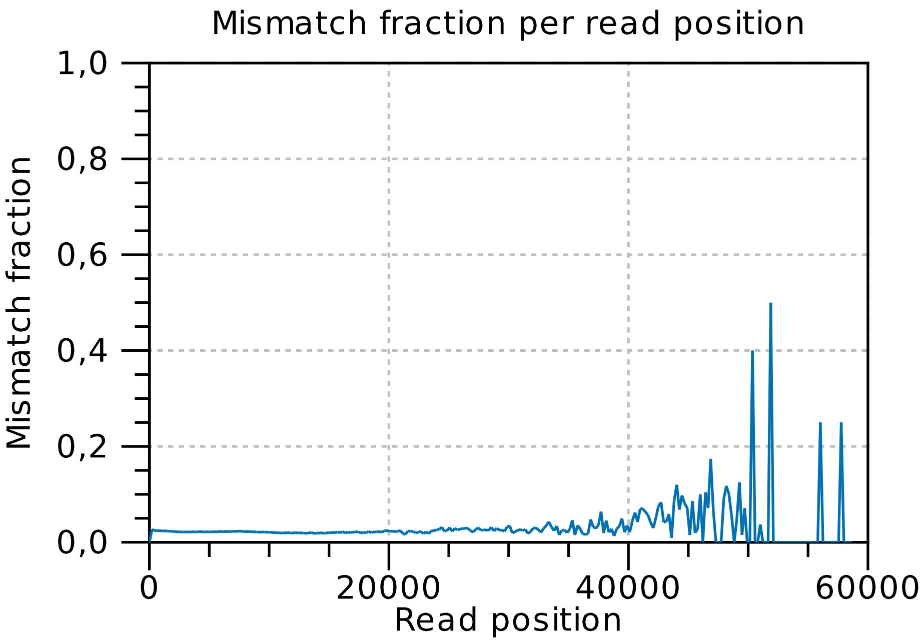

The plot displays the mismatch fraction per read position in the sequenced segments

3.12 Unaligned ends

Read count	1,488,261
% of all mapped reads	165.73
Positions covered	1,332,781
Positions covered in % of reference	1.11
Positions covered in % of bases covered	1.12

Reads with one unaligned end

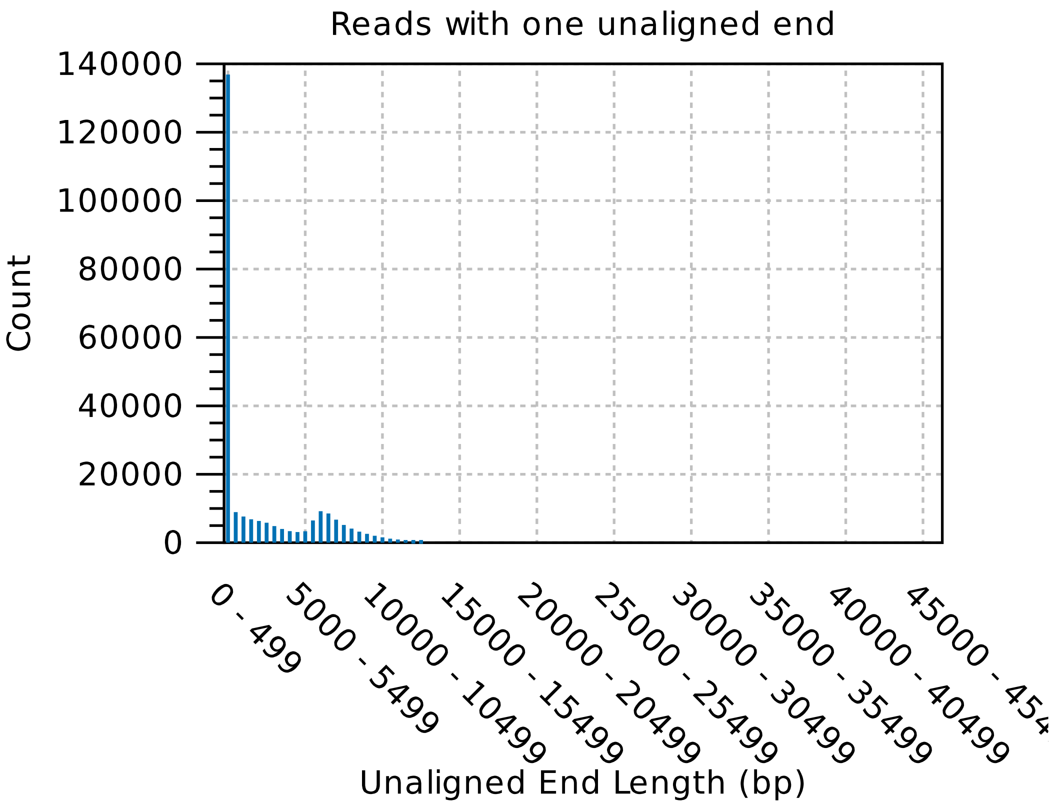

Reads with two unaligned ends

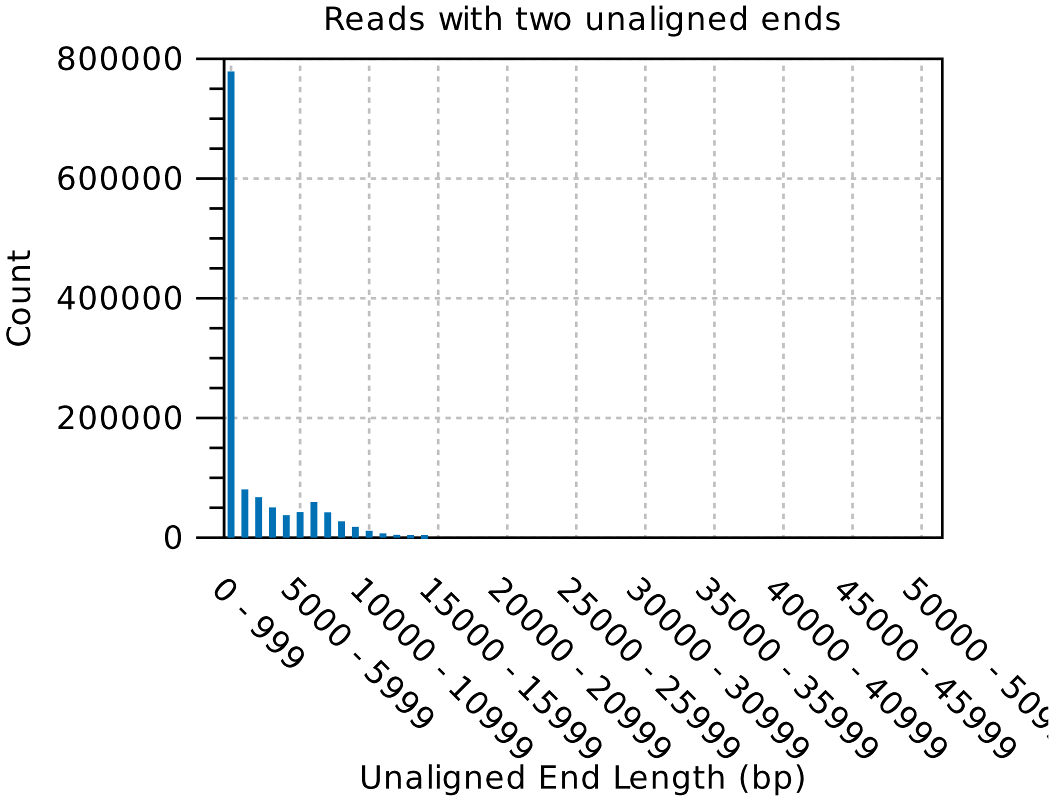

Supplement: Supplementary file 4 — Mapping Report for Ath-WGA BLASR-mapping. (PDF 416 kb) [file 12864_2018_5164_MOESM4_ESM.pdf]
