# Supplementary material for: Correcting palindromes in long reads after whole-genome amplification
Source: BMC Genomics. 2018 Nov 6;19:798. doi: 10.1186/s12864-018-5164-1 (PMC6218980; doi:10.1186/s12864-018-5164-1)
Supplement: Supplementary file 5 — Cumulative lengths of the PacBio-only and hybrid assemblies of Ath-Ctrl, Ath-WGA and Ath-Clean. (DOCX 114 kb) [file 12864_2018_5164_MOESM5_ESM.docx]

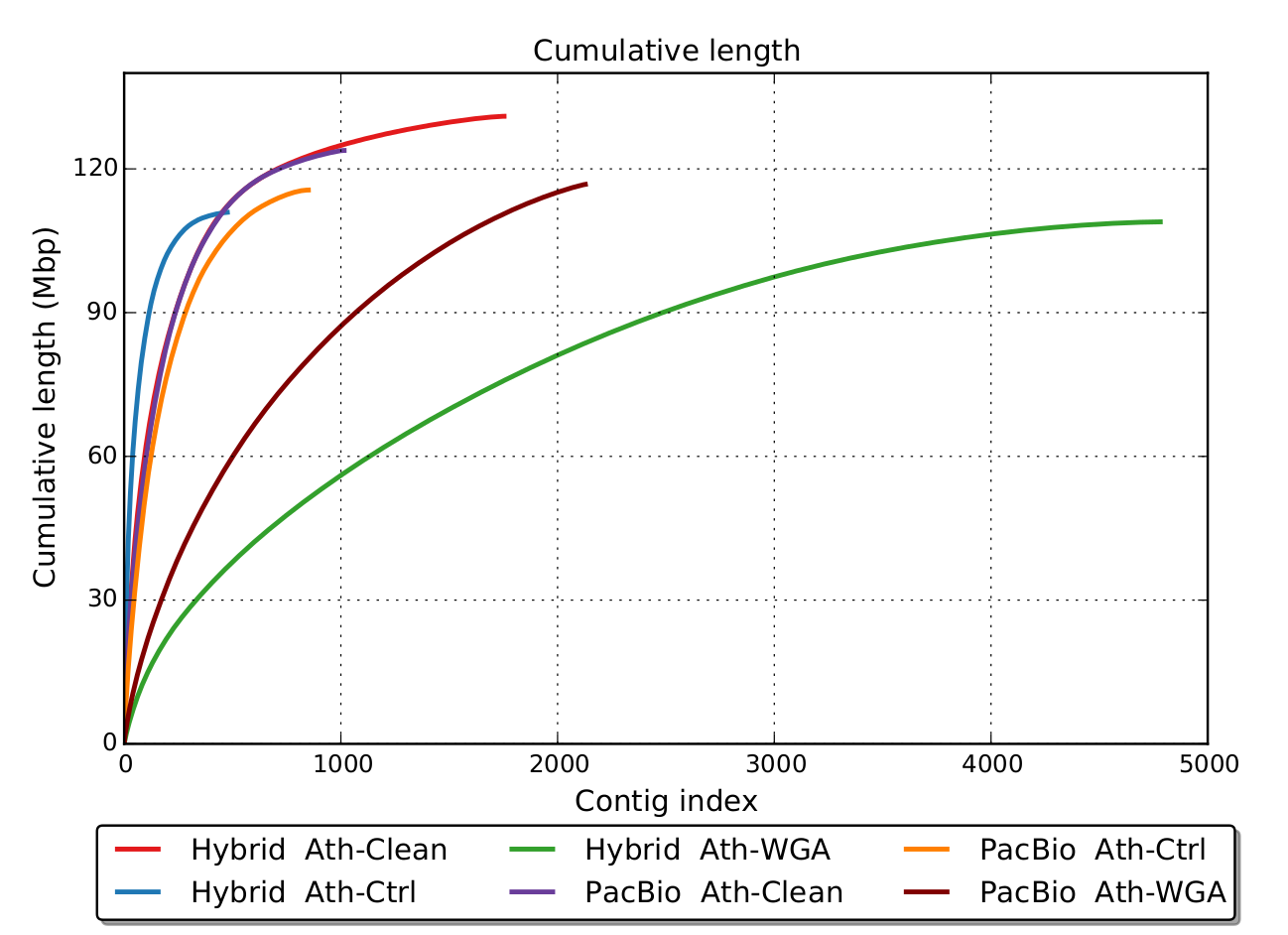


**Suppl. Figure 1.** Cumulative lengths of the PacBio-only and hybrid assemblies of Ath-Ctrl, Ath-WGA and Ath-Clean.
