# Supplementary material for: Correcting palindromes in long reads after whole-genome amplification
Source: BMC Genomics. 2018 Nov 6;19:798. doi: 10.1186/s12864-018-5164-1 (PMC6218980; doi:10.1186/s12864-018-5164-1)
Supplement: Supplementary file 6 — Cumulative length distribution of gorilla Y chromosome assemblies. (DOCX 264 kb) [file 12864_2018_5164_MOESM6_ESM.docx]

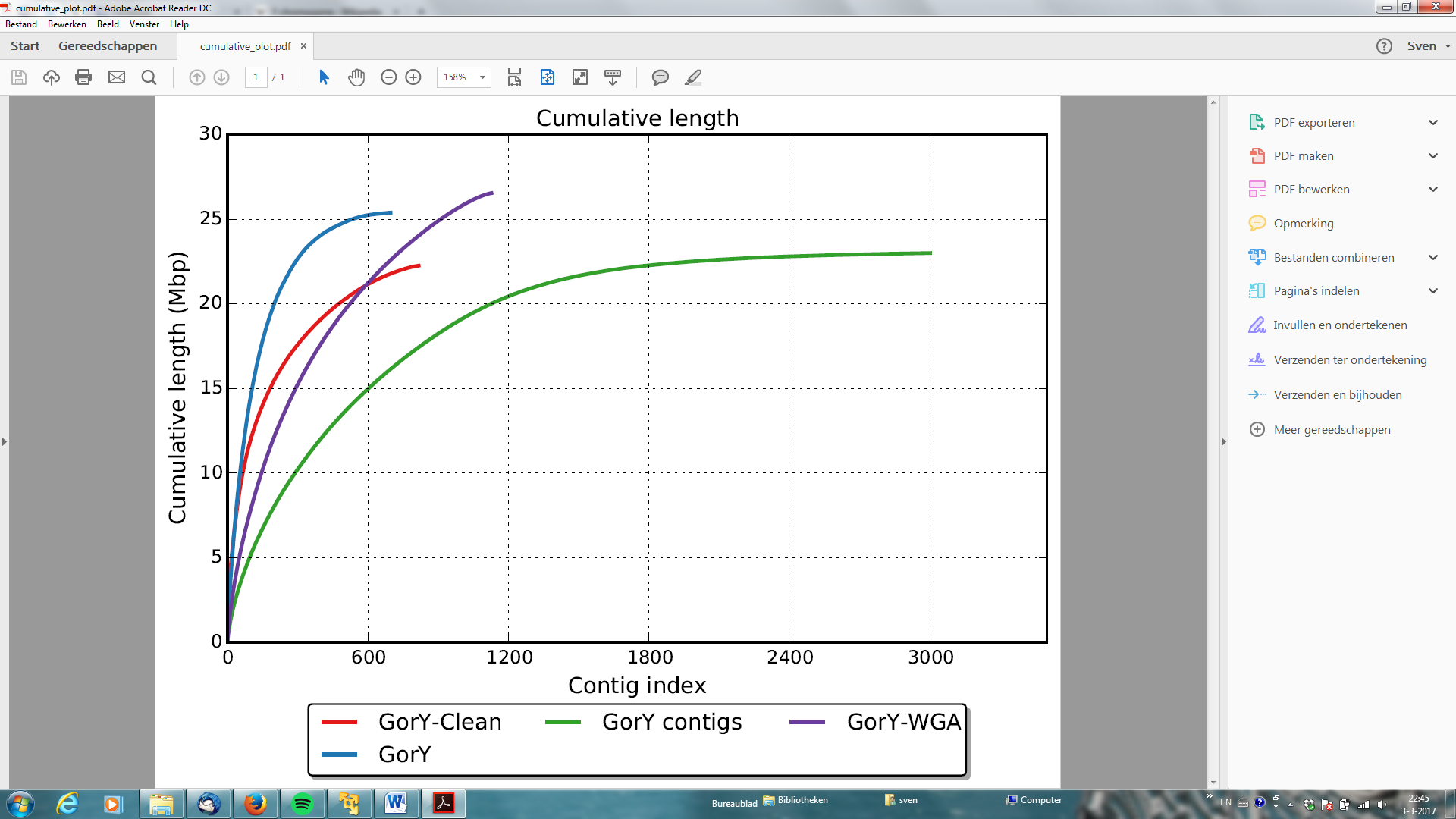


**Suppl. Figure 1:** Cumulative length distribution of gorilla Y chromosome assemblies: GorY is the previously published gorilla Y chromosome scaffolds; GorY contigs indicate the underlying contigs of the scaffolded assembly; GorY-WGA is the *de novo* assembly based on the raw PacBio reads; GorY-Clean is the *de novo* assembled based on the with Pacasus cleaned PacBio reads.
