# Supplementary material for: Correcting palindromes in long reads after whole-genome amplification
Source: BMC Genomics. 2018 Nov 6;19:798. doi: 10.1186/s12864-018-5164-1 (PMC6218980; doi:10.1186/s12864-018-5164-1)
Supplement: Supplementary file 7 — Mummerplots of HumY against the different GorY assemblies. (DOCX 166 kb) [file 12864_2018_5164_MOESM7_ESM.docx]

| 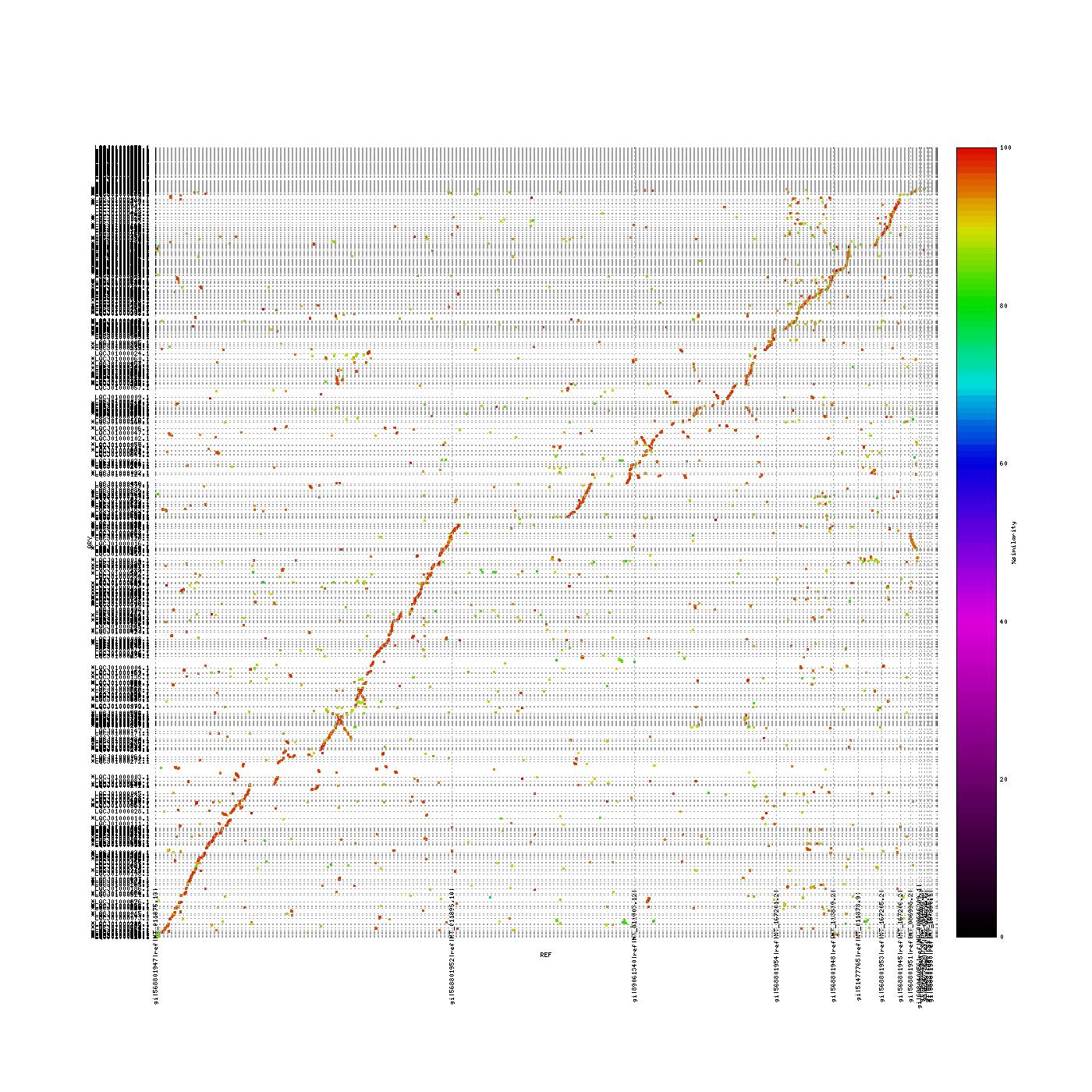  A | 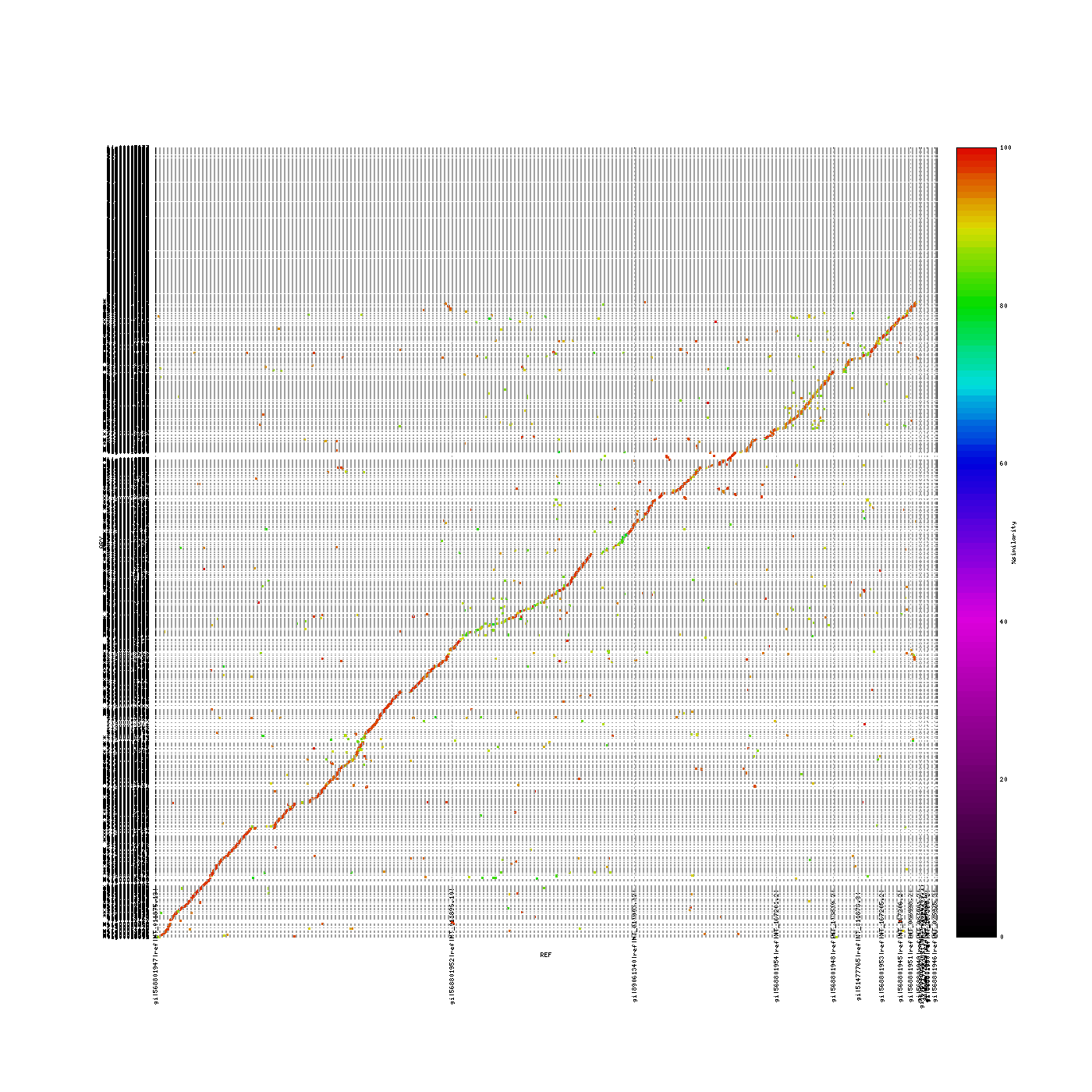B |
| --- | --- |
| 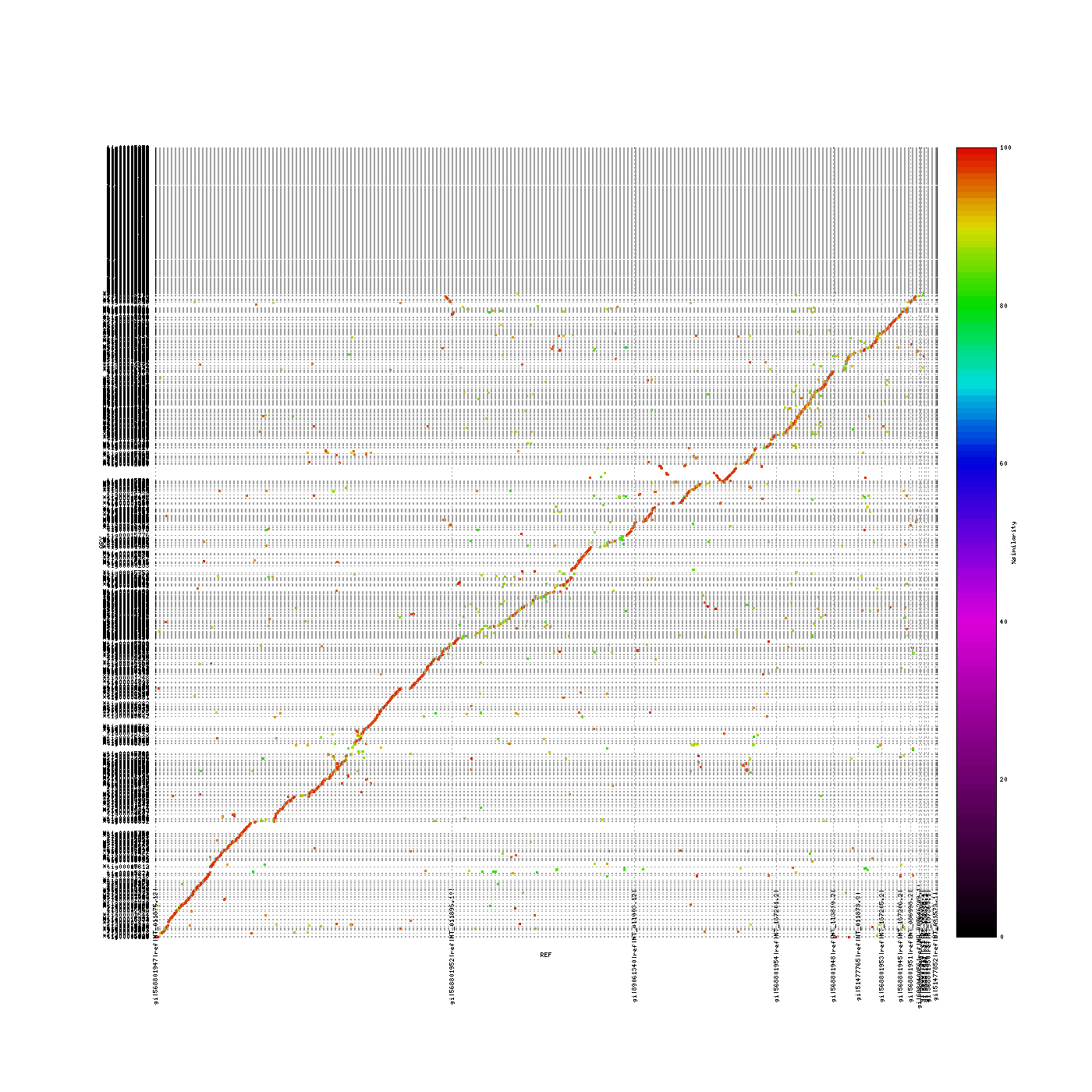C | 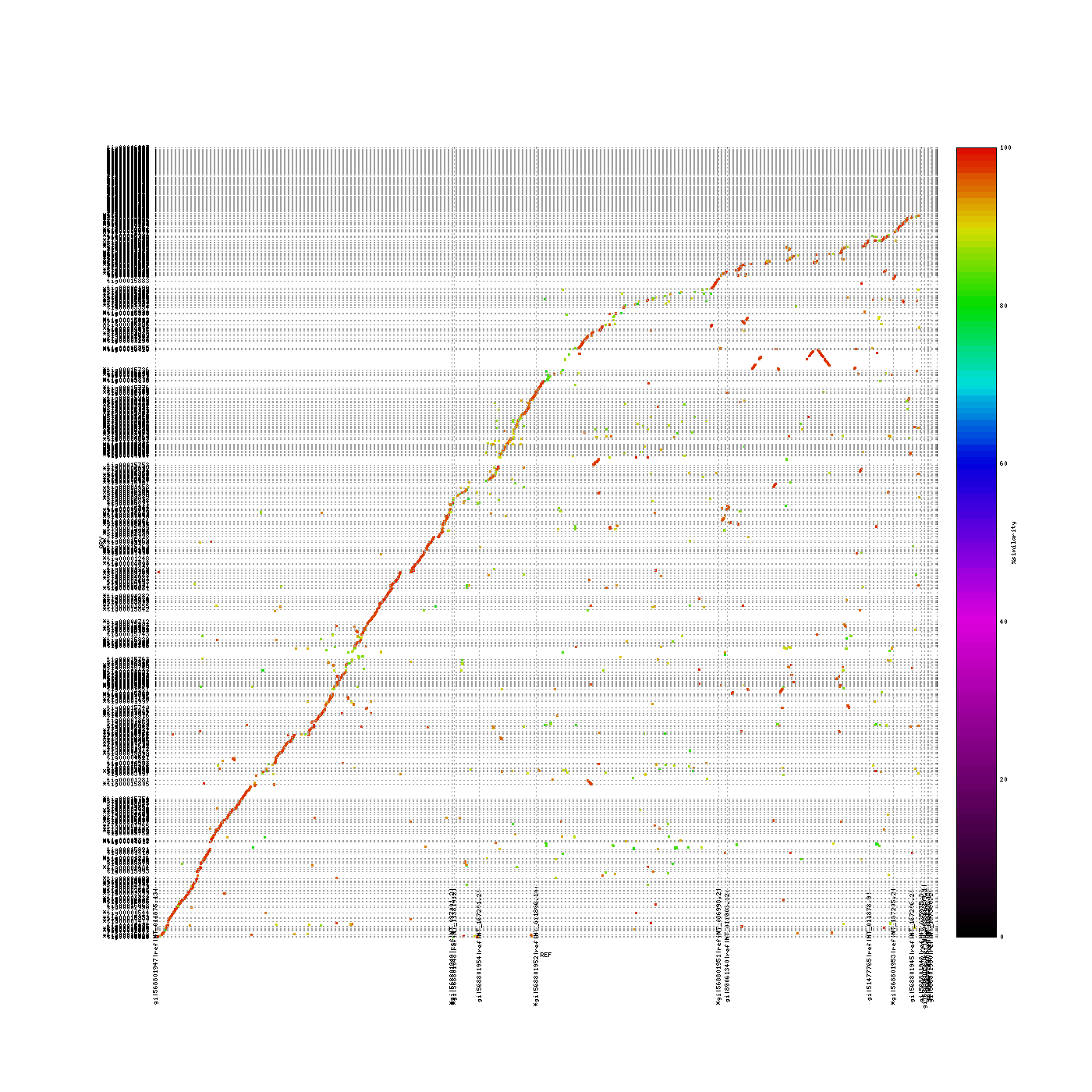D |

**Suppl. Figure 1:** Mummerplots of HumY (x-axis) against the published GorY
chromosome (A), the new GorY-WGA (B) and the new GorY-Clean assembly (C). In (D) contigs of GorY-Clean with over 35 times coverage with PacBio reads are shown.
