# Supplementary material for: Correcting palindromes in long reads after whole-genome amplification
Source: BMC Genomics. 2018 Nov 6;19:798. doi: 10.1186/s12864-018-5164-1 (PMC6218980; doi:10.1186/s12864-018-5164-1)
Supplement: Supplementary file 8 — Coverage and identity of the X-degenerate gene transcripts in GorY and GorY-Clean assemblies. (DOCX 25 kb) [file 12864_2018_5164_MOESM8_ESM.docx]

| Name | GorY Coverage | GorY Identity | GorY-Clean Coverage | GorY-Clean Identity |
| --- | --- | --- | --- | --- |
| AMELY | 63 | 100 | 100 | 99.4 |
| DBY(DDX3Y) | 100 | 94.4 | 100 | 94.7 |
| EIF1AY | 88.3 | 97 | 65 | 98.8 |
| NLGN4Y | 91.5 (2 scaffolds) | 100 | 100 (2 contigs) | 100 |
| PRKY | 43.7 (2 scaffolds) | 99 | 56.1 (2 contigs) | 96.8 |
| SMCY(KDM5D) | 69.7 | 99.4 | 100 | 99.4 |
| SRY | 100 | 81.3 | 100 | 81.5 |
| TBL1Y | 78.4 (2 scaffolds) | 99.8 | 95.6 (2 contigs) | 99.8 |
| TMSB4Y | 99.1 | 98.2 | 100 | 98.1 |
| USP9Y | 52.1 (2 scaffolds) | 97.9 | 55.6 (2 contigs) | 98.0 |
| UTY | 37.6 (2 scaffolds) | 99 | 46.7 (2 contigs) | 99.5 |
| ZFY | 75.2 | 96.1 | 99.8 | 97.0 |
| **Mean** | **74.88** | **96.84** | **84.9** | **96.91** |

**Suppl. Table 1**: Coverage and identity of the X-degenerate gene transcripts in GorY and GorY-Clean assemblies.
