# Supplementary material for: Correcting palindromes in long reads after whole-genome amplification
Source: BMC Genomics. 2018 Nov 6;19:798. doi: 10.1186/s12864-018-5164-1 (PMC6218980; doi:10.1186/s12864-018-5164-1)
Supplement: Supplementary file 9 — Comparison of published GorY assembly to GorY-Clean in X-degenerate genes. (DOCX 34 kb) [file 12864_2018_5164_MOESM9_ESM.docx]

### Supplementary Note.

## Comparison of published GorY assembly to GorY-Clean in X-degenerate genes.

We investigated how X-degenerate genes (XDGs) were represented in the previously published GorY hybrid assembly [1] and in the newly generated GorY-Clean assembly. XDGs are unique to the Y chromosome and are present in single copies, making them a good basis for analyzing the quality of an assembly. The mRNA sequences of 12 XDGs [2] were used as the basis for our comparison. The GMAP [3] (version 2017-03-17; kmer size 15) aligner was used to align these 12 transcripts to GorY and GorY-Clean. Based on the alignment, contigs containing XDGs were identified. For each XDG, we used dot plots (generated using lastz [4] version 1.04.00; parameters --notransition --step=20 --nogapped --format=rdotplot ) to compare the aligning GorY scaffolds and GorY-Clean contigs to each other. Suppl. Figures 11-16 show dot plots for a representative selection of XDGs. We counted the approximate number of inverted duplications, by visually inspecting the dot plots (Additional file 23). The numbers tend to be underestimates, since the length of the contigs/scaffolds is highly variable and there may be inverted duplicates which are not visible at the resolution of the dot plot.

For the contigs aligning to TMSB4Y and DBY, only the gene region has sequence similarity between the two assemblies (Additional files 21-22). The remaining parts of the contigs do not share much similarity. For EIF1AY, the contigs are completely dissimilar (dot plot not shown). These dissimilarities could be explained as either the contigs are disjoint except for the gene part (only the gene is common in both the sequences, the contig constitutes mostly the upstream of the gene and scaffold downstream of the gene or vice versa) or there are misassembles in one or both of the assemblies.

For each XDG transcript, we measured the coverage (i.e. percentage) of each transcript that mapped to each respective assembly, as well as the percent identity of the alignment (Additional file 8). More often than not, XDGs are better represented in GorY-Clean than in GorY. The mRNAs of nine XDGs have more coverage in the GorY-Clean assembly. The mRNA from one XDG (EIF1AY) has higher coverage in the GorY assembly, and the remaining two XDGs have 100% coverage in both assemblies.

We also aligned the contigs/scaffolds from our two respective assemblies to the complete sequences (including both exons and introns) of human XDG genes (obtained from GRCh38 from the UCSC Genome Browser). Addition files 10-16 show the resulting dot plots. Since the sequence diversity between Human and Gorilla genome is low, we expect most of the inversion and duplication events to be missassemblies. An approximate count of inversions and duplications was obtained by a visual inspection of dot plots (Additional file 16). We found that contigs from the GorY-Clean assembly have fewer inversions and duplications in comparison to those of the GorY assembly.

## References

1. Tomaszkiewicz M, Rangavittal S, Cechova M, Sanchez RC, Fescemyer HW, Harris R, et al. A time- and cost-effective strategy to sequence mammalian Y Chromosomes: an application to the de novo assembly of gorilla Y. Genome Res. Cold Spring Harbor Laboratory Press; 2016;26:530–40.

2. Cortez D, Marin R, Toledo-Flores D, Froidevaux L, Liechti A, Waters PD, et al. Origins and functional evolution of Y chromosomes across mammals. Nature. Nature Publishing Group, a division of Macmillan Publishers Limited. All Rights Reserved.; 2014;508:488–93.

3. Wu TD, Watanabe CK. GMAP: a genomic mapping and alignment program for mRNA and EST sequences. Bioinformatics. 2005;21:1859–75.

4. Harris RS. Improved pairwise alignment of genomic DNA. The Pennsylvania State University; 2007.
