# Supplementary material for: Correcting palindromes in long reads after whole-genome amplification
Source: BMC Genomics. 2018 Nov 6;19:798. doi: 10.1186/s12864-018-5164-1 (PMC6218980; doi:10.1186/s12864-018-5164-1)
Supplement: Supplementary file 13 — Dotplots mapping Human PRKY gene to the GorY scaffolds and GorY-Clean contigs. (DOCX 228 kb) [file 12864_2018_5164_MOESM13_ESM.docx]

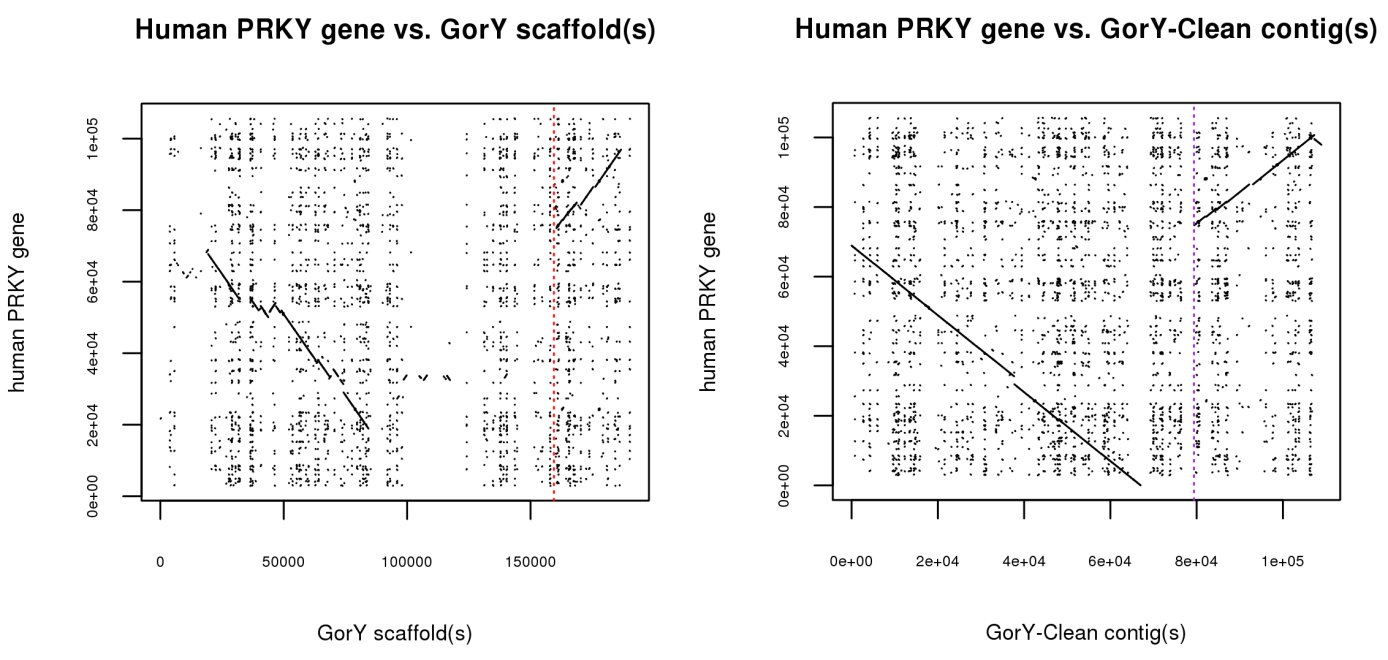


**Suppl. Figure 1:** Dotplots mapping Human PRKY gene (y-axis) to the GorY scaffolds (2 scaffolds on x-axis) on the left and GorY-Clean contigs (2 contigs on x-axis) on the right. We have vertical dotted lines (red for scaffolds, purple for contigs) to separate the two contigs/scaffolds that contain the gene.
