# Supplementary material for: Correcting palindromes in long reads after whole-genome amplification
Source: BMC Genomics. 2018 Nov 6;19:798. doi: 10.1186/s12864-018-5164-1 (PMC6218980; doi:10.1186/s12864-018-5164-1)
Supplement: Supplementary file 15 — Dotplots mapping Human ZFY gene to the GorY scaffold and GorY-Clean contig. (DOCX 46 kb) [file 12864_2018_5164_MOESM15_ESM.docx]

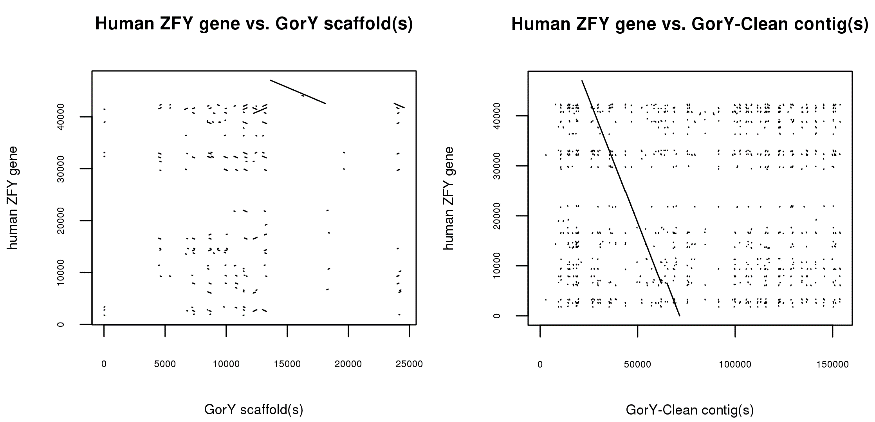


**Suppl. Figure 1:** Dotplots mapping Human ZFY gene (y-axis) to the GorY scaffold (x-axis) on the left and GorY-Clean contig (x-axis) on the right.
