# Supplementary material for: Correcting palindromes in long reads after whole-genome amplification
Source: BMC Genomics. 2018 Nov 6;19:798. doi: 10.1186/s12864-018-5164-1 (PMC6218980; doi:10.1186/s12864-018-5164-1)
Supplement: Supplementary file 16 — The number of duplication or inversion events that were observed on contigs/scaffolds when aligned to human X-degenerate genes. (DOCX 26 kb) [file 12864_2018_5164_MOESM16_ESM.docx]

| Gene | GorY events | GorY-Clean events | Gap present? |
| --- | --- | --- | --- |
| AMELY | 4 | 1 | No |
| DBY(DDX3Y) | 1(Noise) | 0(Noise) | Yes (GorY only) |
| EIF1AY | 1(Noise) | 0 (Noise) | Yes (GorY-Clean only) |
| NLGN4Y | 3 (Noise) | Many (Noise) | Yes (Both) |
| PRKY | >6 (Noise) | 1(Noise) | Yes (Both) |
| SMCY(KDM5D) | 1 (Noise) | 5(Noise) | Yes (GorY only) |
| SRY | 0 | 0 | No |
| TBL1Y | 2(Noise) | >6 (Noise) | Yes (Both) |
| TMSB4Y | 6 | 3 | No |
| USP9Y | 0(Noise) | 1(Noise) | Yes (Both) |
| UTY | 4(Noise) | 1(Noise) | Yes (Both) |
| ZFY | Many (Noise) | 0(Noise) | Yes (GorY only) |
| **Total** | **>28** | **>18** |  |

**Suppl. Table 1:** The number of duplication or inversion events that were observed on contigs/scaffolds when aligned to human X-degenerate genes. The “Gap present?” column indicates the presence of significant gaps in the alignment, which would imply that a part of the human XDG sequence is missing from the assembly. “Noise” indicates the presence of numerous small alignments which appear as black dots in the dot plot. The word “Many” is used when small regions with multiple repetitions represent the alignments, instead of one full stretch.
