# Supplementary material for: Correcting palindromes in long reads after whole-genome amplification
Source: BMC Genomics. 2018 Nov 6;19:798. doi: 10.1186/s12864-018-5164-1 (PMC6218980; doi:10.1186/s12864-018-5164-1)
Supplement: Supplementary file 22 — Dotplots mapping DBY gene containing GorY scaffold to GorY-Clean contig. (DOCX 70 kb) [file 12864_2018_5164_MOESM22_ESM.docx]

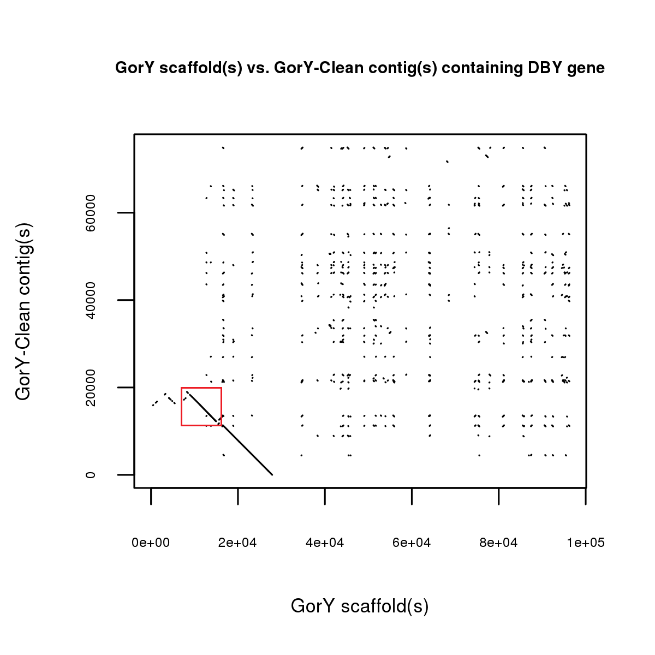


**Suppl. Figure 1**: Dotplots mapping DBY gene containing GorY scaffold (x-axis) to GorY-Clean contig(y-axis). The red box is where the gene is present in the alignment.
