# Supplementary material for: Correcting palindromes in long reads after whole-genome amplification
Source: BMC Genomics. 2018 Nov 6;19:798. doi: 10.1186/s12864-018-5164-1 (PMC6218980; doi:10.1186/s12864-018-5164-1)
Supplement: Supplementary file 23 — The number of inverted duplication events visually observed on XDG-containing contigs/scaffolds when they are aligned to one another in a dotplot. (DOCX 26 kb) [file 12864_2018_5164_MOESM23_ESM.docx]

| Gene | Inverted duplications on GorY scaffolds | Inverted duplications on GorY-Clean Contigs |
| --- | --- | --- |
| AMELY | 0 | 0 |
| DBY(DDX3Y) | 2 | NV(Non-Visible) |
| EIF1AY | NV | NV |
| NLGN4Y | >2 | NV |
| PRKY | >3 | NV |
| SMCY(KDM5D) | >4 | NV |
| SRY | 1 | NV |
| TBL1Y | >7 | NV |
| TMSB4Y | NV | NV |
| USP9Y | NV | NV |
| UTY | NV | NV |
| ZFY | NV | NV |
| **Total** | **>>19** | **0** |

**Suppl. Table 1**: The number of inverted duplication events visually observed on XDG-containing contigs/scaffolds when they are aligned to one another in a dotplot. When no inverted duplications were visible, but there were noisy regions showing multiple smaller alignments, we used the Non-Visible (NV) label.
